# Using host species traits to understand the *Wolbachia* infection distribution across terrestrial beetles

**DOI:** 10.1038/s41598-018-38155-5

**Published:** 2019-01-29

**Authors:** Łukasz Kajtoch, Michał Kolasa, Daniel Kubisz, Jerzy M. Gutowski, Radosław Ścibior, Miłosz A. Mazur, Milada Holecová

**Affiliations:** 1 Institute of Systematics and Evolution of Animals Polish Academy of Sciences, Kraków, Poland; 2 Department of Natural Forests, Forest Research Institute, Białowieża, Poland; 3 Department of Zoology, Animal Ecology and Wildlife Management, University of Life Sciences in Lublin, Lublin, Poland; 4 Institute of Biology, University of Opole, Opole, Poland; 5 Department of Zoology, Faculty of Natural Sciences, Comenius University, Bratislava, Slovakia

## Abstract

Knowledge of *Wolbachia* prevalence with respect to its hosts is restricted mainly to taxonomic/phylogenetic context. In contrast, relations between infection and most host’s ecological and biological traits are poorly understood. This study aimed to elaborate on relations between bacteria and its beetle hosts in taxonomic and the ecological contexts. In particular, the goal is to verify which ecological and biological traits of beetles could cause them to be prone to be infected. Verification of *Wolbachia* infection status across 297 beetle taxa showed that approximately 27% of taxa are infected by supergroups A and B. Only minor support for coevolution between bacteria and its beetle hosts was observed in some genera of beetles, but in general coevolution between beetles and *Wolbachia* was rejected. Some traits of beetles were found to be unrelated to *Wolbachia* prevalence (type of range and thermal preferences); some traits were related with ambiguous effects (habitats, distribution, mobility and body size); some were substantially related (reproduction mode and trophy). The aforementioned summary does not show obvious patterns of *Wolbachia* prevalence and diversity in relation to host taxonomy, biology, and ecology. As both *Wolbachia* and Coleoptera are diverse groups, this lack of clear patterns is probably a reflection of nature, which is characterised by highly diversified and probably unstable relations.

## Introduction

The intracellular α-proteobacterium *Wolbachia* is considered the most abundant endosymbiont of invertebrates. It has been reported as being found in arthropods and filarial nematodes around the world^1,2^. Taxonomy and phylogenetic relations of this bacterium has been topic of intense debate ever since Hertig’s description of *Wolbachia pipientis*^3^. The greatest problems with its classification were caused by the huge diversity of strains described on the basis of selected gene sequencing. This has resulted in the identification of sixteen phylogenetic supergroups, ten of which are found in arthropods, five in nematodes, and one in both groups^4^. Recently, it has been proposed that the different *Wolbachia* supergroups belong to several “*Candidatus* Wolbachia” species^5^; however, this approach has been criticized^6^.

The increased interest in *Wolbachia* has been motivated by the diversity of its phenotypic effects. In arthropods, it can manipulate the host reproduction through male-killing^7^, cytoplasmic incompatibility^8^, parthenogenesis induction^9^, and feminization of genetic males^10^. These effects on host reproduction and development could result in diversification of populations and consequently lead to speciation. The latter could be connected with coevolution of bacteria and its hosts; support for such phenomenon, however, is rare^11^. In contrast, recent studies have shown that *Wolbachia* is not only transmitted vertically (basically matrilineally), but could also spread horizontally using different vectors such as parasites, predators, or common food resources, e.g. host plants^12–16^. This process is probably common and explains the general lack of congruence between *Wolbachia* and host phylogenies, with some exceptions for low-taxonomic levels (e.g. within some genera). Moreover, evidence of horizontal transmission opens numerous possibilities for ecological interactions between bacteria and hosts belonging to various taxonomic units and trophic assemblages.

*Wolbachia* infects numerous species of insects. It is estimated that more than 65% of the species could carry members of the *Wolbachia* clade^17^. This value, however, differs greatly across examined groups. The current state of knowledge about *Wolbachia* infection among some taxonomic groups of nematodes and arthropods has been summarized in several studies (e.g., for: filarial nematodes (Filarioidea)^1^; crustaceans (Crustacea)^18^; spiders (Araneae)^14^; springtails (Collembola)^19^; heteropteran bugs (Heteroptera)^20^; wasps (Hymenoptera: Apocrita)^21^; and butterflies (Lepidoptera)^22^). Surprisingly, knowledge about infection in beetles (Coleoptera) is quite random, as has been recently summarized by Kajtoch & Kotásková^23^. Beetles are the most species-rich and diversified group of organisms in the world, including approximately 386,000 known species^24^. They can be found in most terrestrial and freshwater habitats. Members of Coleoptera belong to all major trophic guilds known in animals. Thus far, only some groups of beetles have been examined with respect to *Wolbachia* infection, but usually on a limited coverage of species (e.g. weevils, Curculionidae and apionids, Apionidae^25,26^; leaf beetles, Chrysomelidae^27,28^; jewel beetles, Buprestidae^29^, and minute moss beetles, Hydraenidae^29^). There are no studies that show prevalence of *Wolbachia* across various families of beetles, with the exception of preliminary works on insects from Panama and North America, where some random species of beetles were included resulting in bacteria detection in 10.5% of beetles from Panama and 13.5% of beetles from North America^30,31^. More importantly, there are also almost no studies that try to solve which biological or ecological features of the hosts could have made them prone or resistant to infection. The only exception is a preliminary work on some European weevils^25^, which was based only on a limited coverage of species and sampling. Systematic review of *Wolbachia* occurrence among beetles has shown that the approximate infection rate in this group is 38%. However, this value varies greatly in different families and genera, and it could be overestimated due to sampling biases (studies focused on groups with members known to be infected). This review rejected cospeciation between *Wolbachia* and Coleoptera, with only some exceptions observed for freshwater Hydraenidae^29^. Finally, this review has shown that, while bacteria effects on hosts are known for some taxa, almost no study has examined the effects of host biological and ecological traits on occurrence and diversity of *Wolbachia*. This is a large gap in knowledge, restricted not only to Coleoptera, but also to insects and all arthropods in general.

This study, by verifying *Wolbachia* infection status across approximately 300 beetle taxa, aimed to solve several questions related to bacteria relations with its beetle hosts. As background for this study, genetic marker data were used for understanding relationships between endosymbiotic bacteria and their beetle hosts in the phylogenetic context. This part aimed to verify whether there is coevolution between *Wolbachia* and its hosts from the Coleoptera (H1). This part was assessed on two levels: for several exemplary genera (H1a) and for superfamilies (H1b). As the presence or the lack of coevolution between beetles and *Wolbachia* does not preclude other associations between bacteria and hosts, we intended to verify the following hypotheses. First, we tested the assumption that *Wolbachia* infection is prevalent in particular taxonomic groups (or that some taxonomic groups of beetles are prone to infection more than others) (H2). Secondly, the presence and distribution of *Wolbachia* strains were analysed in the context of host (beetle) ecological affinity to verify the hypotheses that *Wolbachia* infection is associated with hosts representing particular ecological niches (described by some biological strategies and characteristics and ecological traits of hosts) (H3). This set of hypotheses should allow for the identification of some biological and ecological traits that make (single or together) hosts prone to *Wolbachia* infection. Focus was placed on verification of additional hypotheses centred around trophic affinity of hosts, i.e., i) *Wolbachia* infection is prevalent in beetles belonging to a particular trophic guild (H4a) and ii) *Wolbachia* strains are more related within members of a particular trophic guild (H4b). H4a assume unequal prevalence of *Wolbachia* in predators, herbivores, cambio- and xylophages, saprophages, mycetophages. H4b assume that the presence and distribution of similar (related) *Wolbachia* strains is correlated with host trophic affinity (e.g. that strains within herbivores are more related than strains found in herbivores and predators).

## Results

297 species were selected for molecular examination. The majority of these species were analysed using two to five or more specimens; only some (mainly rare taxa) could be examined using a single representative. These beetles belonged to 37 families and 204 genera. For details about sampling design see Methods section.

### General patterns of *Wolbachia* infection across beetle taxonomy

**Figure 1:**
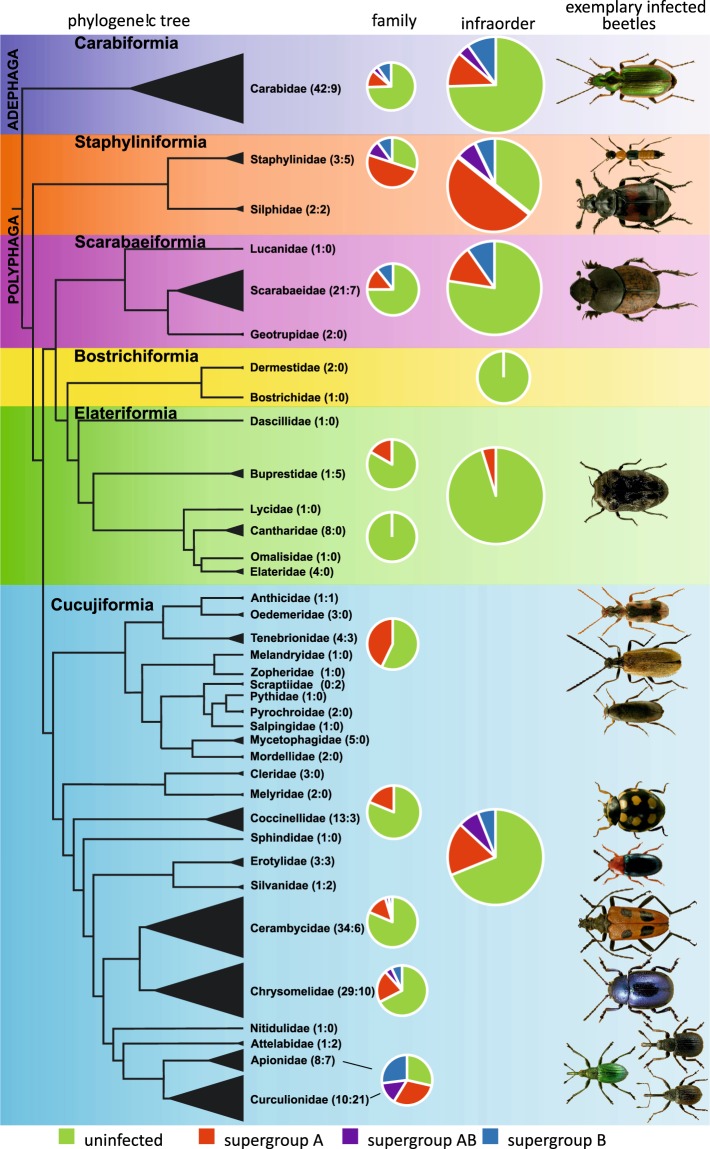
Approximate prevalence of *Wolbachia* infection in selected families and infraorders of beetles. Presented are shares of uninfected species (green), infected species by only supergroup (**A**) (red), only supergroup (**B**) (blue) and by both supergroups (violet). *Wolbachia* prevalence is presented on the background of simplified phylogenetic tree of beetle families considered in the study (reconstructed on the basis of mitochondrial trees topologies from Bocak *et al*.^71^ and McKenna *et al*.^72^). Exemplary infected beetle hosts presented to the right (photographs are reprinted from ICONOGRAPHIA COLEOPTERORUM POLONIAE under a CC BY license, with permission (© Copyright by Prof. Lech Borowiec, Wrocław 2007–2018, Department of Biodiversity and Evolutionary Taxonomy, University of Wrocław, Poland)).

Among the 297 examined beetle species, 81 included *Wolbachia*, which gives an infection rate of 27.3%. For 31 taxa all genes of Multilocus sequence typing (MLST) system^32^ were successfully amplified and sequenced and for remaining 50 taxa only some genes could be successfully amplified and sequenced (for some, amplification succeeded but generated sequences were of poor quality despite several trials). Similar patterns of *Wolbachia* gene amplification and sequencing have been reported in other studies^33^, including studies on beetles^34^. These are usually caused by mismatches in priming sites of standard primers designed for this bacterium due to its great polymorphism^32^. The majority of species were found to be infected in all examined specimens, but one third showed infection only in some specimens. Infection rates in Polyphaga were greater (29%) than in Adephaga (19%) (Supplementary Table S1, Fig. 1). Regarding infraorders, the greatest infection rate was found in Staphyliniformia (54%), followed by Cucujiformia (31%), Scarabaeiformia (23%), Carabiformia (19%), and Elateriformia (5%). No infection was found in Bostrichiformia, but only three species from this infraorder were examined. Infection rates in particular superfamilies were highest in Curculionoidea (59%) and Staphylinoidea (54%), followed by Cucujoidea (25%), Tenebrionoidea (23%), Scarabaeoidea (23%), Chrysomeloidea (20%), Caraboidea (19%), and Buprestoidea (17%). No infection was found in examined members of Bostrichoidea, Cleroidea, Dascilloidea, and Elateroidea (all these were examined either in a single species or a few species). On the level of family, if considering only those families with more than five examined species, the most infected were: Curculionidae (68%), Staphylinidae (63%), Erotylidae (50%), Apionidae (47%), and Tenebrionidae (43%). Lower infection rates were found in Chrysomelidae (26%), Carabidae (19%), Coccinellidae (19%), and Buprestidae (17%); and no infection was found in, e.g. Cantharidae and Mycetophagidae (see details in Supplementary Table S1, Fig. 12).

All infected beetles harboured either strains belonging to supergroup A or B. Supergroup A was found in 64 species (21.5% prevalence); supergroup B in 34 species (11.4% prevalence). Supergroup A was more abundant in Polyphaga (2-times) and nearly the same frequency as supergroup B in Adephaga (1.1-times) (Supplementary Table S1, Fig. 1). Also, in the levels of infraorders and superfamilies, supergroup A prevailed over supergroup B. Supergroup A was prevalent in most families, especially in Cerambycidae (3.5-times) and Staphylinidae (3-times), whereas supergroup B was more abundant only in Apionidae (1.4-times). In some families the share of supergroup A and B was the same (Anthicidae) or very similar (Carabidae, Curculionidae, Scarabaeidae) (Supplementary Table S1).

Infection rates were significantly different only on the level of families (Table 1, Supplementary Fig. S1A). Also, the share of both supergroups differed significantly only between families. On the levels of superfamilies and suborders, these differences were nearly significant (Table 1, Supplementary Fig. S1A).

**Table 1:** Summary of Kruskal-Wallis analysis of variance (ANOVA) calculated for *Wolbachia* (infected vs. uninfected beetle hosts and for hosts infected by supergroup A vs. supergroup B) in respect to major levels of beetles taxonomy.

groups	uninfected *vs* infected			supergroups A *vs* B		
ANOVA	H-test	df	p-value	H-test	df	p-value
**Taxonomy**						
family	**65**.**34**	**36**	**0**.**002**	**23**.**68**	**14**	**0**.**050**
superfamily	8.34	11	0.682	19.29	11	0.056
infraorder	0.38	5	0.996	10.31	5	0.067
suborder	0.07	1	0.793	3.71	1	0.054

In bold: significant tests (p < 0.05).

### *Wolbachia* diversity and co-evolution

Among the 81 infected beetle species, 64 (79%) were found to harbour *Wolbachia* supergroup A and 34 (42%) were found to harbour supergroup B, including 16 species (20%) with strains belonging to both supergroups. The majority of beetle hosts (individuals) were infected by a single strain, but 11 were infected by two or more strains.

**Figure 2:**
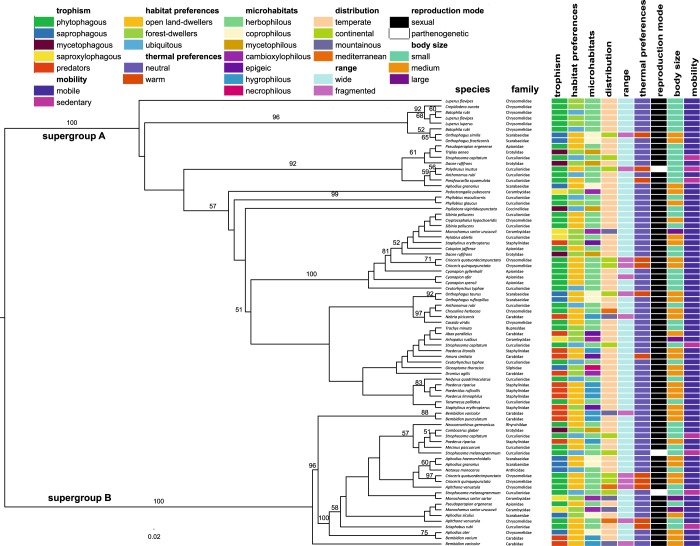
*Wolbachia* phylogenetic tree (reconstructed on the basis of *ftsZ* sequences obtained from infected beetle hosts) with reference to selected ecological and biological traits of the hosts.

Phylogenetic trees of the cell division protein gene (*ftsZ*) showed that *Wolbachia* is highly diversified in beetle hosts (Fig. 2, Supplementary Fig. S2). Both datasets DS1 and DS2 (see Material and Methods for details) confirmed that strains belonging to supergroup A were prevalent over those from supergroup B in beetle hosts. Moreover, the diversity of strains from supergroup A found in beetles (in this study as well as if including hosts from other studies, summarized in Kajtoch & Kotásková^23^) is much greater than those belonging to supergroup B.

Unique *Wolbachia* strains were found in the majority of beetles. In contrast, similar or the same strains were found only rarely, in some closely related species (Fig. 2, Supplementary Fig. S2). Most of these exemplary similarities have already been described in previous works on particular genera (e.g. *Crioceris* leaf beetles^35^, *Cyanapion* apionids^36^; *Polydrusus* and *Eusomus* weevils^37,38^; S*trophosoma* weevils^39^; *Monochamus* longhorn beetles^40^; *Bembidion* ground beetles and *Paederus/Paederidus* rove beetles^41^). Analyses in Procrustes Approach to Cophylogeny (PACo) confirmed coevolution of *Wolbachia* with only *Monochamus* longhorn beetles (P = 0.0364) and *Bembidion* ground beetles (P = 0.0299) (Supplementary Fig. S3). For other groups, cospeciation events were rejected (*Crioceris* leaf beetles, P = 0.7998; *Cyanapion* apionids, P = 0.3599; *Aphodius* dung beetles, P = 0.9685; *Paederus/Paederidus* rove beetles, P = 0.1051) (Supplementary Fig. S3). There were no significant differences in *Wolbachia* strain genetic distances within and between superfamilies of beetle hosts (Supplementary Table S2, U = 43.00, Z = −1.592, P = 0.111).

### General patterns of *Wolbachia* infection across beetle traits

Regarding trophic guilds, *Wolbachia* was most abundant within phytophagous species (38.6%), followed by saprophagous (34.3%), mycetophagous (23.5%), and less abundant in cambioxylophagous (18.5%) and predators (17.6%) (Supplementary Table S1). *Wolbachia* was most abundant among open land-dwellers (32.8%) and less numerous in forest-dwellers (23.0%) and ubiquitous species (22.2%). Considering microhabitats, infection was most prevalent in necrophilous species (40.0%), hygrophilous (36.0%), coprophilous (35.0%), and herbophilous (33.6%); less infected species were found in mycetophilous (26.7%), cambioxylophilous (16.4%), and epigeic (13.2%). The lowest infection rate was found in Mediterranean species (11.8%). Similar rates were found within continental (32.0%), temperate (27.8%), and mountainous (27.3%) species. *Wolbachia* had the same prevalence in species with fragmented ranges (27.1%) and widespread ranges (27.7%). There are also no obvious differences with respect to thermal preferences, as 30.6% species of neutral temperature preferences were infected and 26.7% species of warm preferences were infected. In contrast, a great difference was found between bisexual species (26.3% infected) and parthenogenetic species (80.0% infected, but only five species in this group could be examined). There were also differences in infection rate with respect to body size of the hosts: the most frequently infected hosts were found to be small beetles (40.0%), next medium-sized (21.7%), and finally large taxa (13.0%). Flightless beetles were much more often infected (43.5%) than mobile taxa (25.8%) (Supplementary Table S1).

Across species traits of beetles in almost all states, the more abundant was supergroup A (Supplementary Table S1). Differences were particularly noticeable, for example, in cambioxylophagous (10-times more supergroup A strains than these from supergroup B), mycetophagous (3.0-times), forest-dwellers (4.6-times), epigeic species (4.0-times), necrophilous (all infected by supergroup A), widespread species (2.8-times), or medium size species (2.8-times). The same share of supergroup A and B was found in Mediterranean taxa and large species, and nearly the same share in phytophagous, open land-dwellers, coprophilous, and hygrophilous species (Supplementary Table S1).

According to univariate models, differences between uninfected and infected beetles were best explained by trophism, microhabitats, reproduction mode, and body size (Table 2, Supplementary Fig. S1B). These traits have significant Wald statistics (with exception of microhabitats), lowest Akaike Information Criterion (AIC), and highest Nagelkerke pseudo R2 values (Table 2). Any univariate models comparing infection by supergroups A and B have significant Wald statistics; all have similar AIC and R2 values (Table 2, Supplementary Fig. S1B, Supplementary Fig. S1B).

**Table 2:** Performance of univariate models representing analysed traits of *Wolbachia* hosts (beetles).

trait	d.f.	Wald	p-value	AIC	R2
**unifected** ***vs*** **infected**					
Intercept	1	57.3	0.000	350.7	—
trophism	4	13.5	0.009	344.7	0.046
habitat preferences	2	3.7	0.154	350.9	0.013
microhabitats	6	11.7	0.068	349.5	0.043
distribution	3	2.2	0.534	354.0	0.009
range	1	0.2	0.628	352.5	0.001
thermal preferences	1	0.0	0.936	352.7	0.000
reproduction mode	1	4.6	0.032	346.5	0.021
body size	2	12.7	0.002	341.6	0.043
mobility	1	3.2	0.073	349.6	0.010
**supergroup A** ***vs*** **supergroup B**					
Intercept	1	9.6	0.002	126.4	—
trophism	4	4.0	0.406	129.0	0.054
habitat preferences	2	4.6	0.101	125.2	0.052
microhabitats	6	4.4	0.617	130.3	0.080
distribution	3	0.4	0.947	132.0	0.004
range	1	0.0	0.951	128.4	0.000
thermal preferences	1	0.4	0.515	127.9	0.005
reproduction mode	1	0.1	0.773	128.3	0.001
body size	2	2.2	0.339	128.2	0.022
mobility	1	0.0	0.862	128.4	0.000

**Figure 3:**
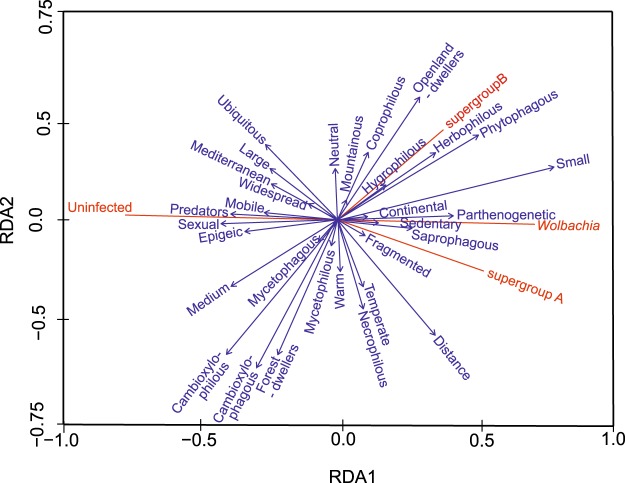
Relationships between the *Wolbachia* infection status in examined beetle hosts and explanatory variables (ecological and biological traits of the hosts) revealed by redundancy analysis (RDA).

Redundancy analysis (RDA) showed that general infection by *Wolbachia* in beetles is associated mostly with parthenogenesis, limited mobility, continental distribution, and small body size, whereas uninfected species are mostly bisexual, mobile, predatorial, and epigeic (Fig. 3). Specifically, infection by supergroup A was associated with fragmented range but not with large body size and Mediterranean distribution. Supergroup B was mostly associated with hydrophilous and herbophilous/phytophagous species but not with cambioxylophagous/philous species (Fig. 3).

Multivariate analyses showed that occurrence of infected beetles is best explained by a model that includes trophism and reproduction mode. Slightly “worse” models also included range/climate and genetic distance (Table 3). Summarized Akaike weights were the highest for reproduction mode, distance, and trophism (Supplementary Table S3). Differences in prevalence of supergroups were best explained by a model that included habitat and other similarly fitted models including trophism and genetic distance (Table 3). Habitat and distance also had highest Akaike weights (Supplementary Table S3).

**Table 3:** Set of competing generalized linear models with binomial error distribution and logit-link function explaining the *Wolbachia* infection status of examined beetle hosts or explaining differences in infection between two supergoups (A and B) of infected hosts on the basis of predictors described in the ‘Methods’ section.

No	Model	k	AIC	Δ	w
**infected** ***vs*** **uninfected**					
	INTERCEPT	0	350.7	8.0	0.001
**1**	**Dist + Trophism + Reprod**	**3**	**342**.**7**	**0**.**0**	**0**.**032**
**2**	**Dist + Range/Climate + Trophism + Reprod**	**4**	**342**.**7**	**0**.**0**	**0**.**032**
**3**	***Trophism + Reprod***	**2**	**342**.**9**	**0**.**2**	**0**.**029**
4	Range/Climate + Trophism + Reprod	3	343.4	0.7	0.023
5	Dist + Habitat + Reprod	3	343.5	0.8	0.022
6	Dist + Reprod	2	343.6	0.9	0.021
7	Dist + Range/Climate + Habitat + Reprod	4	343.8	1.1	0.019
8	Dist + Range/Climate + Habitat + Trophism + Reprod	5	343.9	1.2	0.018
9	Trophism + Body + Reprod	3	343.9	1.2	0.017
10	Dist + Trophism + Body + Reprod	4	344.1	1.4	0.016
11	Dist + Trophism	2	344.1	1.4	0.016
12	Dist + Body + Reprod	3	344.1	1.4	0.016
13	Dist + Range/Climate + Trophism + Reprod + Mobility	5	344.1	1.4	0.016
14	Dist + Range/Climate + Trophism + Body + Reprod	5	344.2	1.5	0.015
15	Range/Climate + Trophism + Body + Reprod	4	344.4	1.7	0.014
16	Dist + Trophism + Reprod + Mobility	4	344.4	1.7	0.013
17	Dist + Habitat + Trophism + Reprod	4	344.5	1.8	0.013
18	Trophism + Reprod + Mobility	3	344.5	1.8	0.013
19	Range/Climate + Trophism + Reprod + Mobility	4	344.6	1.9	0.012
20	Dist + Habitat + Body + Reprod	4	344.6	1.9	0.012
21	Dist + Body + Reprod + Mobility	4	344.6	1.9	0.012
**supergroup A** ***vs*** **supergroup B**					
	INTERCEPT		126.4	3.4	0.011
**1**	**Dist + Trophism**	**2**	**123**.**0**	**0**.**0**	**0**.**062**
**2**	**Dist + Habitat**	**2**	**123**.**1**	**0**.**1**	**0**.**059**
**3**	***Habitat***	**1**	**123**.**7**	**0**.**6**	**0**.**045**
4	Dist + Habitat + Trophism	3	123.9	0.9	0.039
5	Dist + Range/Climate + Habitat	3	124.3	1.3	0.032
6	Dist + Habitat + Body	3	124.5	1.5	0.030
7	Dist + Habitat + Mobility	3	124.5	1.5	0.029
8	Dist + Trophism + Mobility	3	124.6	1.6	0.028
9	Range/Climate + Habitat	2	124.7	1.7	0.026
10	Dist + Habitat + Reprod	3	124.9	1.9	0.024
11	Dist + Range/Climate + Trophism	3	124.9	1.9	0.024
12	Dist + Body + Trophism	3	125.0	2.0	0.023
13	Dist + Trophism + Reprod	3	125.0	2.0	0.023

Only best performed models (with Δ < 2.0) are presented. The number of predictors (k), the Akaike information criterion score (AIC), the difference between the given model and the most parsimonious model (Δ) and Akaike weight (w) are listed for each model. The models with highest (w) are marked in bold; and the best fitted model according to recommendation of Arnold (2010) are marked in italic bold.

There were no significant differences of *Wolbachia* strains genetic distances within and between trophic guilds of beetle hosts (Supplementary Table S2, U = 19.00, Z = −0.674, P = 0.501).

## Discussion

Surprisingly, among numerous studies on *Wolbachia* infection prevalence and interactions with its hosts^1,2^ no study treats with significance host ecological and biological traits. This study brings the first comprehensive analyses showing which host traits might or might not cause some species prone to harbour *Wolbachia* infection. This analysis was executed on the example of beetles – the most diverse order of insects and arthropods, members of which exhibit the majority of ecological and biological traits known for animals. There is extensive knowledge about *Wolbachia* infection in this group (summary in Kajtoch & Kotásková^23^); however, previous data omitted aspects related with general impact of ecological and biological traits on bacteria occurrence. This study substantially improve current state of knowledge about *Wolbachia* infection in Coleoptera as it extend number of inspected species of about 60% (from c. 530 to c. 830 species) and number of infected species of about 40% (from 204 to 271 species)^23^.

Estimated prevalence of *Wolbachia* among beetles in this study – approximately 27% – is lower than most calculations for various groups of insects, which are estimated in a wide range between 20% and 70%^17,20,22^. It is also lower than an estimate based on systematic review across literature about *Wolbachia* infection in beetles (approximately 38%^23^). However, most other estimates were based on non-random sampling, i.e. they were calculated on studies of particular taxonomic group of insects, which were usually selected on the basis of previous knowledge about *Wolbachia* occurrence. Consequently, these studies were focused on groups with a relatively high share of infected taxa. This especially concerned beetles for which the majority of studies were done for weevils (Curculionidae). This group was found to have an exceptionally high *Wolbachia* prevalence (approximately 40%, according to Lachowska *et al*.^25^ and 68% for Curculionidae or 47% for Apionidae in this study). A much lower infection rate in Coleoptera was suggested by geographically-focused studies in which *Wolbachia* was found in only 10.5% of beetles from Panama and 13.5% of beetles from North America^30,31^. These values, which were probably underestimated due to less effective *Wolbachia* detection in early studies, showed that in totally random sampling *Wolbachia* found to be less frequent than in taxonomy-focused studies (e.g. for some particular families).

This study supported a statement that *Wolbachia* is not associated with its hosts’ phylogeny. Among six genera tested in details, only in two cases significant support was found for cospeciation events. *Monochamus* longhorn beetles probably gave false positive results, as only two out of six examined taxa were infected with *Wolbachia* and these two were closest relatives (presumed subspecies)^40^. Only in the case of *Bembidion* ground beetles analyses demonstrate cospeciation between various hosts and bacterial strains (more details in Kolasa *et al*.,^41^). This lack of congruence between beetles and *Wolbachia* phylogeny is frequently reported in studies with only minor exceptions concerning aquatic species like Hydraenidae^29^. Aquatic insects are generally more prone to be infected with *Wolbachia* than terrestrial insects^42,43^. Also, a comparison of genetic distances among *Wolbachia* strains found in beetles show that there are no significant differences between strains found within members of the same superfamily and these from different superfamilies. That general lack of coevolution between beetle hosts and *Wolbachia* should be interpreted in light of the latest conception that *Wolbachia* is not tightly linked with its hosts, but exists as invasion dynamics resulting from horizontal transfers and extinctions^44^. These gains and loses of bacteria explain its global distribution and suggest that *Wolbachia* is in an epidemiological equilibrium^44^. Horizontal transmission of *Wolbachia* has become a major topic of numerous studies and has resulted in increasing evidence that *Wolbachia* can spread not only vertically (maternally) but frequently change its hosts using direct or indirect contacts (such as predators, parasitoids, host plants, and just common habitat^28,35,37,45^). The role of horizontal transfer for spread and diversity of endosymbiotic bacteria is probably unsatisfactory studied and deserves more attention, especially in the light of the recent review by Chrostek *et al*.^46^. Horizontal transfer has been reported in 13% of examined beetle species, but was exceptionally frequent among leaf-eaters such as Curculionidae and Chrysomelidae^23^. Horizontal transmission could also mimic cospeciation if occurring between closely related species sharing the same habitat^35,37^. In such cases, similar *Wolbachia* strains could be present in related species, not because of inheritance of bacteria from common ancestor, but because of bacteria transfer between species, e.g. feeding on the same host plant.

If coevolution is not responsible for *Wolbachia* prevalence among its hosts, here in the example of beetles, what other factors could be responsible? Horizontal transmission is probably an underestimated phenomenon^46^. It can explain widespread occurrence of this bacterium, including presence of similar strains in unrelated hosts. But horizontal transmission could either be random or depend on some ecological and/or biological traits of infected and uninfected species. Surprisingly, there are almost no studies that consider if some traits could make species to be more prone or resistant to *Wolbachia* infection. There are numerous studies which deal with some aspects of hosts’ biological relations with *Wolbachia*, particularly those related with the impact of bacteria on host reproduction. This is why *Wolbachia* is mainly known from its effects including cytoplasmic incompatibility, thelytokous parthenogenesis, feminization of genetic males, male-killing, increased mating success of infected males via sperm competition, and the host’s complete dependence on bacteria for egg production (for reviews see^9,47^). Indeed, among the ecological/biological traits of beetles, reproduction mode was among those that had the greatest impact on *Wolbachia* prevalence. This effect could somehow be biased by unequal sampling in this study, as there are only approximately 2% of parthenogenetic species (and almost all of them were infected). This sampling simply reflects reality because parthenogenesis among European beetles is known almost exclusively from several species of weevils (in general, only approximately 0.15% of beetle species worldwide from 20 families are parthenogenetic^48^). Reproduction is one of the traits for which it is the hardest to solve if the *Wolbachia* is abundant in parthenogens as it induced parthenogenesis, or because this bacteria benefits from spreading across parthenogenetic hosts^49^. This problem has been raised in several studies focused on parthenogenetic weevils (e.g.^34,37,39,50^).

Multiple analyses considering relations between infection and ecological or biological traits of infected and uninfected beetles showed that there is no general pattern that clearly explains this problem. Both infected and uninfected taxa of beetles share various combinations of traits related to their biology and ecology. This could suggest that there are no clear factors that cause some species to be *Wolbachia* infected or not. It seems congruent with the latest finding that *Wolbachia* exists in equilibrium among arthropods, i.e., it spreads frequently among species (mostly via horizontal transmission, rarely via inheritance from common ancestors), and is also lost in some taxa/populations^44^. However, some results suggest (Tables 2 and 3, Fig. 3, Supplementary Fig. S1B) that particular states of some traits are significantly more often present in infected than in uninfected beetles. It is interesting that all traits related with geography (if consider separately), such as their distribution, range and thermal preferences, showed almost no relation with infection status (Table 2, Fig. 3, Supplementary Fig. S1B). On the other hand, in multivariate analyses was the component “Range/Climate” (which was calculated on correlated geographic traits) included in one of models that explained *Wolbachia* occurrence (Table 3 and Supplementary Table S3). Apparently, distribution, range and thermal preferences separately have no visible effect on the occurrence of *Wolbachia*, but the resultant of these traits (here component “Range/Climate”) is more powerful in explanation of *Wolbachia* presence. This is not surprising, as this bacterium is known all over the world – everywhere where potential hosts occur^51^. Moreover, identification of the same or similar strains from hosts in distant parts of the world has proved that *Wolbachia* is widespread and that there are no barriers preventing its distribution as a bacterial taxa. Although there could be some limitations in geographic distribution if consider particular strains. Mobility of beetles showed ambiguous results. This trait was nearly significant in a comparison of infected and uninfected beetles, but was not important in multivariate analyses (Table 3 and Supplementary Table S3). *Wolbachia* was 1.8-times more often found in flightless species than those of high mobility. This could be explained in light of the finding reported above that geography has also ambiguous important meaning for infection when assuming that mobile species should be at least similarly infected as the less mobile if not more so (due to higher chances of meeting infected species). The high prevalence of *Wolbachia* among species with limited mobility could be associated with horizontal transfer in local scale, e.g. within the same habitat or even host plant in a particular area. This is especially noticeable in the case of steppic weevils and leaf beetles, in which many stenotopic and flightless species sharing similar strains^35,37^. Another trait that clearly differentiates infected and uninfected species is body size (Tables 2 and 3, Fig. 3, Supplementary Fig. S1B). Smaller species were 3-times more often infected than the largest ones and 2-times more often infected than medium-size taxa. This pattern is quite unexpected, as there was no other report suggesting such relation. It is hard to find reasonable explanations for this observation. It is rather not related to lower efficiency of PCR on templates from smaller specimens, which should contain less genetic material of bacteria as the smaller species found to be even more frequently infected than the bigger ones. We cannot rule out the possibility that detection of *Wolbachia* was depended on dilution effect of bacterial DNA localized specifically in a tissue (e.g. gonads)^52–54^. However, more simple explanation could be that observed effect was related to a generally lower number of large beetle taxa, which are present in central-east European coleopterofauna and could be collected for this study. Therefore, this hypothesis should be verified on species from tropics, where many more large species exist. Another, even more probable explanation is that small species tend to live in high densities, whereas large species have less numerous populations and some are also territorial. This difference in densities could explain the higher prevalence of bacteria in abundant species which are also often of small size. The most important finding of this study is proof that trophic affinity (as well as habitats and microhabitats, which are correlated with trophy) is probably the most important explanation for *Wolbachia* occurrence in beetles. The most frequently infected were found to be phytophagous species (38.6%), followed by saprophagous (34.3%). The lowest rates of infection among beetles were in mycetophagous (23.5%) and cambioxylophagous (18.5%), with the lowest rates of *Wolbachia* prevalence in predators (17.6%). This pattern is generally congruent with results of previous studies on beetles^23^ as the highest rates of infection among the species were reported from leaf-eating beetles such as weevils and leaf beetles^25,27,28,36,55^. Surprisingly, *Wolbachia* occurrence among saprophagous has been almost overlooked, as few studies have done with, for example, Tenebrionidae^56,57^. Also, there are no reports about *Wolbachia* occurrence among mycetophagous beetles, which indicates how large the “white spots” are in studies on *Wolbachia* in beetles. Regarding cambioxylophagous, almost all previous knowledge on this group was based on a single study on Buprestidae^29^ and single studies on Cerambycidae (only a few members of the genus *Monochamus* were tested^40,58^). The same concern for predatory species of terrestrial beetles exists, as there are only single studies on ground beetles^59^, rove beetles^60^ and ladybird beetles^61,62^. Interestingly, predatory beetles from aquatic environments (e.g. Dytiscidae, Hydraenidae) are more frequently infected^29^ than terrestrial beetles. This pattern could be associated with the higher densities of aquatic predatory beetles compared to terrestrial predatory beetles, as even riverine (not aquatic) ground and rove beetles have been found to have a high prevalence of bacteria^41^. The lowest prevalence of *Wolbachia* among predators is an unexpected result, as this group should have numerous occasions to gain infection via feeding on infected species. Especially that some possibility has been provided by studies that detected DNA from the bacterium in uninfected predators that fed on infected prey^63–65^. Direct foraging is probably not an efficient way for *Wolbachia* transmission. A possible explanation is that the predator-prey route cannot transfer *Wolbachia* because the symbionts may not be able to survive in the predators’ digestive tracts^18^.

It is interesting to not only find traits related to infection of *Wolbachia* but also to identify those traits that differentiate hosts infected by various supergroups of this bacteria. Beetles are known to be infected by three supergroups. Yet supergroup F has thus far only been found in three taxa: *Agrilus araxenus* and *Lamprodila mirifica* (both Buprestidae^29^) and *Rhinocyllus conicus* (Curculionidae^66^). Among the Coleoptera examined in this study, no species harboured supergroup F, a fact that additionally supports the rare presence of this supergroup among beetle hosts. Previous data has shown that in the four groups of beetles with the highest numbers of examined and infected species, the distributions of supergroups has varied: it was equal in Buprestidae^29^; A overdominated B in Hydraenidae and Chrysomelidae^28,29,67^ and B overdominated A in Curculionoidea (true weevils with apionids)^25,34,45^. This study shows that supergroup A overdominated B in a majority of families. The only exceptions was Apionidae, with prevailed over supergroup B, whereas in Carabidae and Scarabaeidae both supergroups infected a similar number of taxa. All these data suggest that diversity of *Wolbachia* across beetle families is not random, as supergroup A overdominates B with rare exceptions in some families.

## Conclusions

In summary, data above concerning *Wolbachia* prevalence among Coleoptera demonstrate that there is only minor support for coevolution between bacteria and the beetle hosts. Cospeciation only occurred in some genera, usually between closely related species (H1a confirmed in some cases). However, this effect could be mimicked in some cases by horizontal transmission of bacteria between taxa sharing the same environment. In general, coevolution between beetles and *Wolbachia* has not played a substantial role in the spread and diversification of both groups (H1b rejected). Moreover, significant differences in *Wolbachia* prevalence among particular taxonomic groups of beetles can be observed on the family level, but not on higher taxonomic ranks (H2 confirmed for families, rejected for superfamilies, infraorders and suborders).

Regarding the biological and ecological traits of beetles, some of them were found to be unrelated to *Wolbachia* prevalence (e.g. thermal preferences and range), with ambiguous effect on bacteria occurrence (such as mobility and body size) and some traits found to be substantially related with infection (reproduction mode and trophy). In summary, some states of particular ecological and biological traits of beetles could be used as good explanatory factors for *Wolbachia* prevalence. However, in general, there are no obvious combinations of these traits that clearly allow for the differentiation of uninfected and infected beetles (as well as those infected by various supergroups) (H3 rejected). Reproduction mode, a trait that explains infection, has been already described as related to *Wolbachia*. The best explanatory trait of *Wolbachia* prevalence has been found to be trophic affinity of beetles (H4a confirmed). However, the diversity of *Wolbachia* was unrelated with affinity to particular trophic guild by its hosts (H4b rejected). It needs to be stated that abovementioned conclusions are based on observational data (rather simple information about presence or absence of the bacteria in examined species) and are mostly correlative analyses, what does not prove a direct causal link. Causes and mechanisms of relations among hosts traits and the infection by *Wolbachia* (or any other endosymbiotic bacteria; see Duron *et al*.^68^) need to be investigated in future studies focused on some exemplary taxa and the best with use of more sophisticated methods (like next-generation sequencing technologies). There are simultaneous research to the study presented here, which elaborate infection by other endosymbiotic bacteria (namely *Rickettsia*, *Spiroplasma*, and *Cardinium*) on the same set of beetles (Kolasa *et al*., in press), as well as characterize the whole microbiome of selected beetle species on large number of specimens (Kolasa *et al*., unpublished).

This summary does not demonstrate obvious patterns of *Wolbachia* prevalence and diversity in relation to host taxonomy, biology, and ecology. As both *Wolbachia* and Coleoptera are diverse groups (there are numerous supergroups of bacteria raised to the level of species and thousands taxa of beetles that could be characterized by a wide spectrum of traits), such result should not be unexpected. This lack of clear patterns is most probably a reflection of nature, which is characterised by highly diversified and probably unstable relations (if one considers *Wolbachia* prevalence to be constant gains and losses, which explains its global distribution and suggests an epidemiological equilibrium between bacteria and its potential hosts).

## Materials and Methods

### Sampling design

Beetle species were collected during several field trips organized across central, eastern, and southeastern Europe from 2014 to 2017. The majority of species were collected from various sites in Poland, Slovakia, Romania, and Bulgaria; some taxa were also taken from Czechia, Germany, Austria, Hungary, Croatia, Greece, Ukraine, and Belarus (192 sites in total, Supplementary Table S4, Supplementary File 1). Special attention was given sampling in two highly diverse areas in central Europe: Białowieża Forest (mainly for forest-dwellers, particularly cambioxylophagous species) and the Carpathians (for species from most types of environments, particularly rivers, valleys, forests, and grasslands). Beetles were searched for in fields using numerous standard entomological techniques (using sweep-net, sieve, light-capturing, traps for scavengers, searching in dead wood, mushrooms, etc.) by experienced entomologists – specialists in various groups of beetles. Specimens were immediately preserved in 96% ethanol, then deposited in laboratories in −20°C. The specimens were morphologically identified by expert taxonomists using appropriate keys and handbooks. The nomenclature adopted in this study follows that of the Catalogue of Palaearctic Coleoptera (see Supplementary File 2 for references).

Finally, more than 400 species were collected and preserved, most of them from a few or several localities. These species were selected on the basis on three major features: i) to cover a wide spectrum of taxonomic groups of beetles known from central, eastern, and south-eastern Europe; ii) to cover all trophic guilds with a relatively large number of taxa, iii) to include in analyses two to three or more specimens per species, each from different localities if possible. Finally, after examining the available collection of beetles, 297 species were selected for molecular examination (Supplementary Table S5).

### Species traits

Several biological and ecological traits were identified from selected beetle species for further analyses. These traits were selected on the basis of general available knowledge about beetles biology and ecology, taken from various studies (see Supplementary File 2 for references) and based on expert knowledge of specialists involved in this study.

To avoid confusing nomenclature in the paper, groups of ecological/biological features are called “traits” (e.g. trophic guild) while different types of traits are called as “states” (e.g. predators). Beetles (based on the biology and ecology of imaginal stages) were assigned to states of nine selected traits: i) trophic guilds, ii) habitat types, iii) microhabitats, iv) distribution, v) ranges, vi) thermal preferences, vii) reproduction modes, viii) body size and ix) mobility. Details about these traits and characteristics of rules according to which species were assigned to particular states are presented in Table 4.

**Table 4:** Characterization of biological and ecological traits and their states used for beetle species assignment for purposes of this study.

Trait	State	Description	No of assigned species
trophic guild	predators	species which kill and feed on other animals	91
	herbivores	species which feed on living (green) plant tissues	101
	mycetophages	species which feed on living mushrooms	17
	saprophages	species which feed on dead organisms (except dead wood)	35
	cambioxylophages	species which feed on wood and phloem	53
habitat type	forest-dwellers	species which live only in forested habitats	112
	open land-dwellers	species which live only in grassland habitats	131
	ubiquitous	species which could live in all types of terrestrial habitats	54
microhabitats	coprophilous	species which inhabit animal feces	20
	epigeic	species which inhabit soil surface (free living)	38
	mycetophilous	species which inhabit mushrooms	15
	herbophilous	species which inhabit living plants	128
	hygrophilous	species which inhabit wet habitats (but not aquatic)	25
	necrophilous	species which inhabit carcases	5
	cambioxylophilous	species which inhabit inside trees	66
distribution	continental	species of xeric habitats in Pontic and Pannonian regions (mostly steppes)	25
	mediterranean	species of dry habitats in Mediterranean basin (the Balkans)	17
	mountainous	species of high mountain zones (the Sudetes, the Carpathians and the Balkan Mts.)	11
	temperate	species widespread in central Europe except above listed areas	244
range	fragmented	species with fragmented and isolated populations	36
	widespread	species with continuous ranges	261
thermal preferences	neutral	species which wide tolerance to temperatures	250
	warm	species which prefer only warm temperatures	47
reproduction mode	bisexual	species which reproduce only bisexually	292
	parthenogenetic	species which parthenogenetic reproduction (at least in central Europe)	5
body size	large	species larger than 20 mm length	23
	medium	species of 10–20 mm length	174
	small	species smaller than 10 mm length	100
mobility	mobile	species winged and capable to fly	274
	sedentary	species apterous (flightless)	23

### Laboratory works

Before DNA extraction, all specimens were cleaned using ethanol and distilled water in order to reduce the risk of external contamination. DNA was extracted from the whole insect body (for beetles up to approximately10 mm length; only from abdomen for larger specimens) using the Nucleospin Tissue kit (Macherey-Nagel), following the manufacturer’s instructions. The mitochondrial cytochrome oxidase subunit I gene (*cox1*) was amplified using polymerase chain reaction (PCR) with the following primers: LCO-1490/HCO-2198 or LepF1/LepR1 or, in case of failure of the previous two, C1-J-2183/TL2-N-3014^69,70^. The concentration of reagents used for the amplification of *cox1* marker and the cycling profile for PCR followed^36^. *Cox1* was amplified first as the control for PCR efficiency and second as the DNA marker for genetic distance calculations. We were not able to sequence the same *cox1* fragment for all species (this problem is known from barcoding projects on beetles, see e.g.^71^; therefore, some species have *cox1* fragments from the 3′ or 5′ site of this gene). Moreover, we downloaded *cox1* from GenBank for several species (see Supplementary Table S4). Different sets of *cox1* sequences presented no problems, as this study did not intend to do phylogenetic analyses on all the beetles (this topic has already been described in several papers, e.g.^71,72^). Homologous *cox1* were available for coevolutionary analyses (tree reconstructions and distance calculations) for selected genera (see below).

The presence of *Wolbachia* in particular beetles was first screened using two sets of primers amplifying *Wolbachia* surface protein (*wsp*) and cell division protein (*ftsZ*) (primers and PCR conditions follow https://pubmlst.org/Wolbachia/). Two controls were used in this step: negative (samples with distilled water instead of DNA isolates) and positive (DNA isolates from *Polydrusus inustus* weevil, which is known to be infected in its entire range^38^). Next, positive samples were amplified with other genes of Multilocus sequence typing (MLST) system^32^, that is: aspartyl/glutamyl-tRNA(Gln) amidotransferase, subunit B (*gatB*); cytochrome c oxidase, subunit I (*coxA*); conserved hypothetical protein (*hcpA*); and fructose-bisphosphate aldolase (*fbpA*). In case of multiple infection by different *Wolbachia* supergroups, specific primers were used according to MLST protocols (https://pubmlst.org/wolbachia/info/amp_seq_double.shtml). Cloning of PCR products prior to sequencing was not adopted due to a large number of examined species, and lack of obvious signs of the presence of multiple strains from the same supergroup within DNA isolates from particular individuals (based on examination of chromatograms). Although multiple infections are not so rare in insects (see e.g.^73–76^), the majority of such examples concern the presence of different strains within species or populations, but rarely within particular individuals. Moreover, even in this study, some multiple-infected beetles were overlooked, this should not have consequences for analyses and conclusions, as this study did not aim to examine the overall diversity of *Wolbachia* in particular hosts. After DNA purification (Exo-BAP Kit; EURx, Poland), the PCR fragments (all – *cox1* and *Wolbachia* genes) were sequenced using a BigDye Terminator v.3.1. Cycle Sequencing Kit (Applied Biosystems) and performed with an ABI 3100 Automated Capillary DNA Sequencer. All newly generated sequences (both from beetles and bacteria) were submitted to GenBank (see Supplementary Table S4) for accession numbers.

### Co-evolutionary analyses

On the level of genus, six genera were selected, as within these groups those species that were infected and uninfected by *Wolbachia* were identified; moreover, infected species harbour various bacteria stains. Moreover, selected genera belong to different beetle families and various trophic guilds. Among selected genera were *Cyanapion* apionid weevils (phytophagous, six species); *Crioceris* leaf beetles (phytophagous, five species); *Monochamus* longhorn beetles (cambioxylophagous, six species); *Aphodius* dung beetles (saprophagous, ten species); *Bembidion* ground beetles (predators, six species); and *Paederus*/*Paederidus* rove beetles (predators, six species). For this part of analysis, data newly generated in this study as well as sequences obtained from previous published works on beetle-*Wolbachia* associations were used^35,36,40,41^. For all these groups, phylogenetic trees were reconstructed for both the *cox1* of beetles and the selected MLST genes of *Wolbachia* from infected hosts. Maximum likelihood trees of *cox1* were reconstructed using IQ-TREE web server http://www.iqtree.org/77 under the following settings: auto selection of substation model; ultrafast bootstrap approximation (UFBoot)^78^ with 10,000 iterations; maximum correlation coefficient = 0.99; single branch test with use of the approximate Likelihood-Ratio Test (SH-aLRT)^79^; and other default options. Trees of MLST were inferred with ClonalFrame 1.2, software that infers clonal relationships from MLST data and incorporates recombination events^80^. Three independent runs were performed with 500,000 generations each and a burn-in of 20%. The convergence of runs was assessed using methods of Gelman and Rubin^81^ implemented in ClonalFrame. All post-burn-in trees were used to build a majority-rule consensus tree and infer posterior probabilities from clade frequencies. Finally, both trees were used for visualization of relations between phylogeny of the hosts (beetles) and bacteria (*Wolbachia*). To directly test for congruence between *Wolbachia* and beetle trees, the Procrustean Approach to Cophylogeny (PACo) software was used^82^. This method provides test statistics to assess whether phylogenetic positions of corresponding hosts (beetles) and symbionts (*Wolbachia*) are independent of one another. This is achieved via randomization of host-symbiont associations. This test requires distance matrices of hosts and symbionts, so genetic distances were calculated on the MLST dataset for *Wolbachia* strains and the *cox1* gene for beetles (for each genera separately) using the “dist.dna” function of the R package and the TN93 model^83^. PACo was performed within the R statistical environment^84^, using both types of distance matrices and 100,000 permutations each.

Diversity of *Wolbachia* strains was compared within and between superfamilies of examined beetles based on genetic distances (adopting Kimura-two parameters as the substitution model^85^ calculated for *wsp* sequences). *Wsp* was selected for this part of analyses because it is more polymorphic than any MLST gene and because in this study the largest dataset of strains from examined beetles was collected for this gene. The Mann-Whitney U test was used to assess the difference in distances within and between *Wolbachia* strains found in superfamilies of beetles. Additionally, differences in prevalence of *Wolbachia* in particular taxonomic levels of its beetle hosts were estimated using Kruskal-Wallis one-way analysis of variance (ANOVA) for each level separately.

### *Wolbachia* diversity

For a brief estimation of *Wolbachia* diversity, the maximum likelihood phylogenetic trees were reconstructed using IQ-TREE under conditions described above for two datasets: DS1, which includes all newly obtained *ftsZ* sequences of *Wolbachia* generated from beetles in this study, and DS2, which includes the aforementioned sequences plus all other *ftsZ* sequences of *Wolbachia* found in beetle hosts. Data was taken from the review of Kajtoch & Kotásková^23^. Among MLST genes, only the *ftsZ* sequences were chosen for these analyses because this gene has the largest database of sequences (e.g. NCBI GenBank, *Wolbachia* MLST) available from various hosts belonging to Coleoptera.

### *Wolbachia*-host traits statistics

Box plots visualized differences in *Wolbachia* prevalence in groups of hosts assigned to particular traits (also with respect to infection by two supergroups). Moreover, performance of univariate models (each including single trait) was assessed using Wald statistics, Akaike Information Criterion (AIC), and Nagelkerke pseudo R2. Redundancy analysis (RDA) was used for the visualization of relations among infection status (uninfected, infected, as well as infected by both supergroups) and ecological and biological traits of the beetles.

Next, correlations among traits were assessed (Spearman rank correlation). Numerous states of particular traits were found to be significantly correlated. This especially concerned trophic guilds and microhabitats (e.g. mycetophagous with mycetophilous, cambioxylophagous with cambioxylophilous, phytophagous with herbophilous, etc.). This also concerned states of habitat traits (forest-dwellers and open land-dwellers) and the majority of states from traits: range, distribution, and thermal preferences. To avoid using these correlated variables in multivariate analyses, some of them were omitted (microhabitats due to high correlation with trophic guilds). Other correlated variables were grouped into components using Principal Component Analysis (PCA) (Habitats, which include all states of this trait, PC1 = 56.3%; Range/Climate, which include most states from range, distribution, and thermal preferences traits, PC1 = 56.9%). Finally, uncorrelated traits, the aforementioned components of correlated states and *cox1* distances among beetle species (adopting Kimura-two parameters as the substitution model^85^) were used as explanatory variables in multivariate analyses. Generalized Linear Models (GLMs) with binomial distribution were constructed for two datasets: GLM1 – for all beetle species (297) with *Wolbachia* infection as the explained variable (infected *vs* uninfected); and GLM2 – only for infected beetles (81) with occurrence of supergroups as the explained variable (supergroup A *vs* supergroup B). Performance of multivariate models was estimated using AIC, delta (∆), and Akaike weight (*w)*. Following Arnold^86^, we treated parameters which did not improve the AIC of a model by more than 2.0 as uninformative.

Finally, diversity of *Wolbachia* strains was compared within and between trophic guilds of examined beetles based on genetic distances (adopting Kimura-two parameters as the substitution model^85^ calculated for *wsp* sequences (the same as above for superfamilies). Difference in distances within and between *Wolbachia* strains found in trophic guilds of beetles was assessed using the Mann-Whitney U test.

Statistical analyses were performed in the R statistical environment^84^ or with STASTISTICA v.11^87^ and PAST 3.20 software^88^.

### Ethical note

This study complied with European, Polish, Czechian, Slovakian, Austrian, Hungarian, Romanian, Bulgarian, Croatian, Greek, Ukrainian and Belarusian regulations regarding the collection of invertebrates (beetles). No of collected and examined species of beetles is protected under European or national laws (there are no regulations of sampling of not-protected beetles in countries where sampling was executed).

## Supplementary information

Supplementary tables, figures and files

Supplementary Dataset 1

Supplementary Dataset 2

Supplementary Dataset 3

Supplementary Dataset 4

Supplementary Dataset 5

## Data Availability

The authors declare that data supporting the findings of this study are available within the paper and the supplementary information files.
