# Supplementary material for: Using host species traits to understand the Wolbachia infection distribution across terrestrial beetles
Source: Sci Rep. 2019 Jan 29;9:847. doi: 10.1038/s41598-018-38155-5 (PMC6351630; doi:10.1038/s41598-018-38155-5)
Supplement: Supplementary file 1 — Supplementary tables, figures and files [file 41598_2018_38155_MOESM1_ESM.pdf]

Supplementary tables, figures and files to the article:

Using host species traits to understand the *Wolbachia* infection distribution across terrestrial beetles

Łukasz Kajtoch, Michał Kolasa, Daniel Kubisz, Jerzy M.Gutowski, Radosław Ścibior, Miłosz A. Mazur, Milada Holecová

SCIENTIFIC REPORTS

DOI:10.1038/s41598-018-38155-5

Supplementary Table S1. Summary of *Wolbachia* infection status with respect to taxonomy of examined beetle hosts (A) and with respect to ecological and biological traits of examined beetle hosts (B).

A – taxonomy

group	family																												
taxonomic units	Anthicidae	Apionidae	Bostrichidae	Buprestidae	Cantharidae	Carabidae	Cerambycidae	Chrysomelidae	Cleridae	Coccinellidae	Curculionidae	Dascillidae	Dasytidae	Dermestidae	Elateridae	Erotylidae	Geotrupidae	Lucanidae	Lycidae	Melandryidae	Mordellidae	Mycetophagidae	Nitidulidae	Oedemeridae	Omalisidae	Pyrochroidae	Pythidae	Rhynchitidae	Salpingidae
species	2	15	1	6	8	47	38	39	3	16	31	1	2	2	4	6	2	1	1	1	2	5	1	3	1	2	1	3	1
infected	1	7	0	1	0	9	6	10	0	3	20	0	0	0	0	3	0	0	0	0	0	0	0	0	0	0	0	1	0
A	0	0	0	1	0	4	5	7	0	3	10	0	0	0	0	2	0	0	0	0	0	0	0	0	0	0	0	0	0
B	0	3	0	0	0	3	0	1	0	0	5	0	0	0	0	1	0	0	0	0	0	0	0	0	0	0	0	1	0
AB	1	4	0	0	0	2	1	2	0	0	5	0	0	0	0	0	0	0	0	0	0	0	0	0	0	0	0	0	0

group	family								superfamily												infraorder						suborder	
taxonomic units	Scarabaeidae	Scraptiidae	Silphidae	Silvanidae	Sphindidae	Staphylinidae	Tenebrionidae	Zopheridae	Bostrichoidea	Buprestoidea	Caraboidea	Chrysomeloidea	Cleroidea	Cucujoidea	Curculionoidea	Dascilloidea	Elateroidea	Scarabaeoidea	Staphylinoidea	Tenebrionoidea	Cucujiformia	Bostrichiformia	Elateriformia	Carabiformia	Scarabaeiformia	Staphyliniformia	Polyphaga	Adephaga
species	28	2	4	1	1	8	7	1	3	6	47	78	5	24	49	1	14	31	13	26	182	3	21	47	31	13	250	47
infected	7	2	2	1	0	5	3	0	0	1	9	16	0	6	29	0	0	7	7	6	57	0	1	9	7	7	72	9
A	4	2	2	1	0	4	3	0	0	1	4	12	0	5	11	0	0	4	6	5	33	0	1	4	4	6	44	4
B	3	0	0	0	0	0	0	0	0	0	3	1	0	1	9	0	0	3	0	0	11	0	0	3	3	0	14	3
AB	0	0	0	0	0	1	0	0	0	0	2	3	0	0	9	0	0	0	1	1	13	0	0	2	0	1	14	2

B – ecological/biological traits																														
traits	trophic guild					habitat preferences			microhabitats							distribution				range		thermal prefe-rences		repro-duction		body size			mobi-lity	
states	phytophagous	mycetophagous	predators	saprophagous	cambioxylophagous	forest-dwellers	open land-dwellers	ubiquitous	coprophilous	epigeic	herbophilous	hygrophilous	mycetophilous	necrophilous	cambioxylophilous	continental	mediterranean	mountainous	temperate	widespread	fragmented	warm	neutral	parthenogenetic	sexual	large	medium	small	mobile	sedentary
species	101	17	91	35	53	112	131	54	20	38	128	25	15	5	66	25	17	11	244	251	46	35	262	5	292	23	174	100	274	23
infected	39	4	16	12	10	26	43	12	7	5	43	9	4	2	11	8	2	3	68	68	13	11	70	4	77	3	38	40	71	10
A	18	3	10	8	9	21	21	6	4	4	21	4	3	2	10	4	1	1	42	39	9	6	42	2	46	1	26	21	42	6
B	10	1	3	3	0	3	13	1	3	1	10	2	1	0	0	1	1	0	15	15	2	1	16	1	16	1	5	11	14	3
AB	11	0	3	1	1	2	9	5	0	0	12	3	0	0	1	3	0	2	11	14	2	4	12	1	15	1	7	8	15	1

Supplementary Table S2. Basic statistics describing intra- and inter-group genetic distances calculated for *Wolbachia* strains found in superfamilies and trophic guilds of examined beetles.

variable	N	Average	Min	Max	SD
superfamilies					
intra	7	9.6	5.7	16.1	3.8
inter	21	11.6	7.3	16.1	2.5
trophic guilds					
intra	5	9.3	6.5	11.4	2.4
inter	10	10.5	8.3	13.0	1.4

Supplementary Table S3. Sums of Akaike Information Criterion weights obtained from Generalized Linear Models (presented in Table 5) for selected predictors explaining either infection status of examined beetle hosts (*Wolbachia* infected vs uninfected) or infection by two supergroups (A vs B).

Variable	infected *vs* uninfected	supergroups A *vs* B
Distance	**0.530**	**0.548**
Trophism	**0.511**	0.319
Range/Climate	0.420	0.309
Habitat	0.404	**0.506**
Body	0.460	0.305
Reproduction mode	**0.571**	0.276
Mobility	0.435	0.313

Supplementary Table S4. Characteristics of sampling of beetle species for Wolbachia screening with accession numbers to GenBank (cox1 - cytochrome oxidase subunit I gene generated from beetle DNA and Wolbachia housekeeping genes: wsp - Wolbachia surface protein, gatB - aspartyl/glutamyl-tRNA(Gln) amidotransferase, subunit B; coxA - cytochrome c oxidase, subunit I; hcpA - conserved hypothetical protein; ftsZ - cell division protein, and fructose-bisphosphate aldolase). Italicized cox1 numbers downloaded from GenBank from other studies (italicized and underlined – single sequences of congeneric taxa for species which missed cox1, used for distances calculation). Distribution of sampling sites is presented on supplementary file 1.

**Species**	**Country**	**Locality**	**Year**	**Voucher**	***coxI***	***wsp***	***gatB***	***coxA***	***hcpA***	***fbpA***	***ftsZ***
Abax parallelepipedus	Poland	Chełm	2015	W00218a	MH020426						
Abax parallelepipedus	Poland	Chełm	2015	W00218b	MH020427						
Abax parallelus	Poland	Szyk	2014	W00232a	MH020429	MG987893	MG987944	MG987643	MG987739	MG987691	MG987977
Adalia bipunctata	Poland	Białowieża Forest	2016	W00131a	MH115505						
Adalia bipunctata	Poland	Koryciny	2015	W00131b	MH115506						
Agapanthia villosoviridescens	Poland	Białowieża Forest	2016	W00055a	*KU919520*						
Agapanthia villosoviridescens	Poland	Białowieża Forest	2016	W00055b	+						
Agapanthia villosoviridescens	Poland	Białowieża Forest	2016	W00055c							
Agonum marginatum	Slovakia	Andrejovka	2017	W00211a	MH020395						
Agonum marginatum	Poland	Lipnica Wielka	2017	W00211b		MG987900			MG987723		
Agonum marginatum	Poland	Tolkmicko	2017	W00211c							
Agrilus derasofasciatus	Poland	Białowieża Forest	2016	W00234a	MH115496						
Agrilus suvorovi	Poland	Białowieża Forest	2016	W00075a	MH115551						
Agrilus suvorovi	Poland	Borowiec Radom	2016	W00075b							
Agrypnus murinus	Poland	Białowieża Forest	2016	W00244a	MH115536						
Alosterna tabacicolor	Poland	Rogalin	2016	W00081a	MH020351						
Alosterna tabacicolor	Poland	Białowieża Forest	2016	W00081b							
Alosterna tabacicolor	Poland	Grzybowa	2017	W00081c	MH020352						
Altica oleracea	Bulgaria	Anevo	2015	W00018a	MH020485					+	
Altica oleracea	Bulgaria	Paweł Banja	2015	W00018b	MH020486						
Altica oleracea	Bulgaria	Karlovo	2015	W00018c	MH020487						
Amara similata	Poland	Chełm	2015	W00227a	MH020396	MG987834	MG987946	MG987641	MG987749	MG987699	MG987979
Amphimallon solstitialis	Poland	Kędzierzyn-Koźle	2017	W00065a	MH020490						
Amphimallon solstitialis	Poland	Suchy Bór	2017	W00065b	MH020491						
Anaspis brunnipes	Poland	Szymiszów	2017	W00167a	MH020278	MG987889			+		
Anaspis brunnipes	Poland	Szymiszów	2017	W00167b	MH020280	MG987890					
Anaspis brunnipes	Poland	Szymiszów	2017	W00167c	MH020279						
Anaspis frontalis	Poland	Kędzierzyn-Koźle	2017	W00166a	MH020275	MG987880			+		
Anaspis frontalis	Poland	Kędzierzyn-Koźle	2017	W00166b	MH020276						
Anaspis frontalis	Poland	Kędzierzyn-Koźle	2017	W00166c	MH020277	MG987881			+		
Anaspis frontalis	Poland	Dursztyn	2014	W00166d		MG987882			+		
Anastrangalia reyi	Poland	Białowieża Forest	2016	W00261a	MH020347	MG987848	MG987955	MG987634	+	+	MG988052
Anastrangalia reyi	Poland	Białowieża Forest	2016	W00261b	MH020348	MG987849	MG987956	MG987646		+	+
Anastrangalia sanguinolenta	Poland	Białowieża Forest	2016	W00262a	MH020349						
Anastrangalia sanguinolenta	Poland	Białowieża Forest	2016	W00262b	MH020350						
Anatis ocellata	Poland	Porytowe Wzgórze	2015	W00225a	MH020519						
Anomala dubia	Poland	Knyszyn Forest	2016	W00062a	MH115533						
Anomala dubia	Poland	Białowieża Forest	2016	W00062b	+						
Anomala dubia	Poland	Łysaków	2016	W00062c	+						
Anoplodera sexguttata	Poland	Białowieża Forest	2016	W00082a	MH020345						
Anoplodera sexguttata	Poland	Poznachowice	2017	W00082b	MH020346						
Anoplodera sexguttata	Poland	Kępie	2017	W00082c							
Anoplotrupes stercorosus	Poland	Grajów	2017	W00067a	MH020522						
Anoplotrupes stercorosus	Bulgaria	Troian	2015	W00067b	MH020523						
Anoplotrupes stercorosus	Poland	Białowieża Forest	2014	W00067d	MH020524						
Anostirus castaneus	Poland	Białowieża Forest	2014	W00138a	MH115530						
Anostirus castaneus	Poland	Łapsze Niżne	2014	W00138b	+						
Anthaxia nitidula	Poland	Sromowce Niżne	2014	W00073a	MH115561						
Anthaxia nitidula	Austria	Porrau	2015	W00073b	+						
Anthaxia nitidula	Poland	Grzybowa	2016	W00073c	+						
Anthaxia quadripunctata	Poland	Chrusty	2015	W00074a	*KM452657*						
Anthaxia quadripunctata	Poland	Białowieża Forest	2014	W00074b							
Anthonomus rubi	Ukraine	Zvenyhorod	2010	W00016a	MH020261	MG987847	MG987937	+	MG987761	MG987704	MG988013
Anthonomus rubi	Hungary	Lénárddaróc	2012	W00016b	+						
Anthonomus rubi	Poland	Ślesin	2010	W00016c	+				MG987770		MG987983
Aphodius ater	Romania	Fenes	2016	W00122a	MH020313	MG987780					MG988043
Aphodius ater	Poland	Lipnica Wielka	2017	W00122b	MH020314	MG987781					MG988044
Aphodius depressus	Romania	Fenes	2016	W00084a	MH020525						
Aphodius depressus	Romania	Fenes	2016	W00084b	MH020526						
Aphodius depressus	Poland	Wielopole	2015	W00084c	MH020527						
Aphodius depressus	Bulgaria	Troian	2015	W00084d	+						
Aphodius depressus	Bulgaria	Troian	2015	W00084e	+						
Aphodius erraticus	Poland	Wielopole	2015	W00123a	+						
Aphodius erraticus	Romania	Islaz	2016	W00123b							
Aphodius erraticus	Poland	Wielopole	2015	W00123c							
Aphodius fossor	Bulgaria	Balkanec	2015	W00124a	MH020305						
Aphodius fossor	Poland	Wielopole	2015	W00124b	MH020306						
Aphodius granarius	Bulgaria	Kalofer	2015	W00118a		+					MG988014
Aphodius granarius	Romania	Fenes	2016	W00118b	MH020315	+					MG988040
Aphodius granarius	Bulgaria	Kalofer	2015	W00118c		+					MG988041
Aphodius haemorrhoidalis	Poland	Sromowce Niżne	2014	W00119a	MH020316	MG987859					
Aphodius haemorrhoidalis	Poland	Wielopole	2015	W00119b		MG987860					
Aphodius haemorrhoidalis	Poland	Niebrzegów	2015	W00119c		MG987861					
Aphodius luridus	Romania	Fenes	2016	W00120a	MH115495						
Aphodius luridus	Poland	Sromowce Niżne	2014	W00120b	+						
Aphodius luridus	Poland	Wielopole	2015	W00120c							
Aphodius pedellus	Bulgaria	Troian	2015	W00090a	MH020528						
Aphodius pedellus	Poland	Wielopole	2015	W00090b	MH020529						
Aphodius pedellus	Poland	Niebrzegów	2015	W00090c	MH020530						
Aphodius pusillus	Romania	Fenes	2016	W00087a	MH020307						
Aphodius pusillus	Poland	Sromowce Niżne	2014	W00087c							
Aphodius pusillus	Bulgaria	Kalofer	2015	W00087h							
Aphodius scrutator	Bulgaria	Kalofer	2015	W00125a	MH020364						
Aphodius scrutator	Romania	Tanacu	2016	W00125b	MH020365						
Aphodius sphacelatus	Bulgaria	Troian	2015	W00089b	MH020308						
Aphodius sphacelatus	Bulgaria	Balkanec	2015	W00089d							
Aphodius sphacelatus	Poland	Lipnica Wielka	2015	W00089g							

Aphodius sticticus	Bulgaria	Kalofer	2015	W00126a	MH020310	MG987862	MG987911		MG987721	MG987675	MG988033
Aphodius sticticus	Bulgaria	Rocafer	2015	W00126b	MH020311	MG987863	MG987912	MG987660	MG987722	MG987676	MG988032
Aphodius sticticus	Romania	Fenes	2016	W00126c	MH020312						
Aphthona venustula	Poland	Trzebinia-Bożniowa Mt.	2015	W00035a	MH020465	MG987870	MG987918		MG987731	MG987677	MG988042
Aphthona venustula	Bulgaria	Balkanec	2015	W00035b							
Aphthona venustula	Bulgaria	Karvare	2015	W00035c		MG987871	MG987919	MG987659	MG987732	MG987678	MG988031
Arhopalus rusticus	Poland	Odolion ad Ciechocinek	2016	W00273a	MH020361	MG987891		MG987637	MG987740	+	MG987982
Arhopalus rusticus	Poland	Białowieża Forest	2014	W00273b	MH020362	+		MG987636		+	
Aromia moschata	Poland	Borowa	2015	W00224a	MH020476						
Athous subfuscus	Poland	Białowieża Forest	2014	W00280a	MH115535						
Attagenus pellio	Poland	Białowieża Forest	2014	W00279a	MH115500						
Batophila rubi	Poland	Wdżar Mt.	2014	W00036a	MH115564	MG987841	+		+	+	+
Batophila rubi	Poland	Dursztyn	2014	W00036b		MG987842			+		
Batophila rubi	Bulgaria	Balkanec	2015	W00036c		MG987843	+	+	+	+	+
Bembidion articulatum	Poland	Tarnawa	2015	W00148a	MH020421						
Bembidion articulatum	Poland	Rdzawa	2015	W00148b	MH020422						
Bembidion articulatum	Slovakia	Rokytov	2017	W00148f							
Bembidion decorum	Poland	Nowa Biała	2014	W00149c	MF170685						
Bembidion decorum	Slovakia	Rokytov	2017	W00149d	MF170684						
Bembidion decorum	Bulgaria	Paweł Banja	2015	W00149f	MF170683						
Bembidion modestum	Romania	Moldova	2016	W00257a	MF170689						
Bembidion modestum	Czechia	Kopytov	2016	W00257b	MF170688						
Bembidion modestum	Poland	Fałkowice	2017	W00257c	MF170686						
Bembidion punctulatum	Poland	Domosławice	2017	W00256a	MF170693		MF170749	MF170763	MF170777	MF170805	MF170791
Bembidion punctulatum	Romania	Moldova	2016	W00256b	MF170695		MF170751	MF170765	MF170779	MF170807	MF170793
Bembidion punctulatum	Czechia	Kopytov	2016	W00256c	MF170694		MF170750	MF170764	MF170778	MF170806	MF170792
Bembidion punctulatum	Poland	Fałkowice	2017	W00256d	MF170691		MF170748	MF170762	MF170776	MF170804	MF170790
Bembidion varicolor	Poland	Domosławice	2017	W00150a	MF170679	+	MF170743	MF170757	MF170771	MF170799	MF170785
Bembidion varicolor	Romania	Moldova	2016	W00150b	MF170681	+	MF170746	MF170760	MF170774	MF170802	MF170788
Bembidion varicolor	Romania	Moldova	2016	W00150c			MF170747	MF170761	MF170775	MF170803	MF170789
Bembidion varicolor	Poland	Fałkowice	2017	W00150d	MF170678		MF170742	MF170756	MF170770	MF170798	MF170784
Bembidion varicolor	Slovakia	Andrejovka	2017	W00150e	MF170680		MF170744	MF170758	MF170772	MF170800	MF170786
Bembidion varicolor	Slovakia	Topľa river valley	2017	W00150f			MF170745	MF170759	MF170773	MF170801	MF170787
Bembidion varium	Poland	Lipnica Wielka	2017	W00258a	MH020423	MG987855	MG987913	MG987662	MG987728	MG987679	MG988047
Bembidion varium	Romania	Moldova	2016	W00258b	MH020424	MG987856	MG987915	MG987663	MG987730	MG987680	MG988045
Bembidion varium	Bulgaria	Paweł Banja	2015	W00258c	MH020425	MG987907	MG987914	MG987664	MG987729	+	MG988046
Bitoma crenata	Poland	Szyk	2014	W00134a	MH020302						
Bitoma crenata	Poland	Szyk	2014	W00134b	MH020303						
Bitoma crenata	Poland	Łążek	2017	W00134c							
Blitopertha lineolata	Bulgaria	Anevo	2015	W00116a	MH115532						
Blitopertha lineolata	Bulgaria	Anevo	2015	W00116b	+						
Blitopertha lineolata	Bulgaria	Paweł Banja	2015	W00116c							
Bostrichus capucinus	Austria	Füllersdorf	2016	W00072a	MH115570						
Bostrichus capucinus	Poland	Grajów	2017	W00072b							
Brachyleptura maculicornis	Poland	Rogalin	2016	W00083a	*KU255691*						
Brachyleptura maculicornis	Poland	Chrusty	2015	W00083b							
Broscus cephalotes	Poland	Nowe Sady	2014	W00222a	MH020419						
Broscus cephalotes	Poland	Odolion ad Ciechocinek	2016	W00222b	MH020420						
Bruchidius cf. varius	Poland	Kędzierzyn-Koźle	2014	W00037a	MH020271						
Bruchidius cf. varius	Poland	Kędzierzyn-Koźle	2014	W00037b							
Bruchidius cf. varius	Poland	Paweł Banja	2015	W00037c							
Bruchus affinis	Poland	Sromowce Wyżne	2014	W00019a	MH020262						
Bruchus affinis	Poland	Radzionków	2014	W00019b	MH020263						
Bruchus affinis	Poland	Ligota Dolna	2015	W00019c							
Bruchus atomarius	Poland	Wdżar Mt.	2014	W00020a	MH319816						
Bruchus atomarius	Poland	Zdzieszowice	2015	W00020b	+						
Bruchus atomarius	Poland	Jarmuta Mt.	2014	W00020c	+						
Bruchus brachialis	Poland	Nowa Wieś	2014	W00038a	MH020270						
Bruchus brachialis	Poland	Kędzierzyn-Koźle	2014	W00038b							
Bruchus brachialis	Poland	Kędzierzyn-Koźle	2014	W00038c							
Bruchus loti	Poland	Sromowce Wyżne	2014	W00021a	MH020267						
Bruchus loti	Poland	Ligota Dolna	2015	W00021b	MH020268						
Bruchus loti	Bulgaria	Balkanec	2015	W00021c	MH020269						
Bruchus viciae	Bulgaria	Anevo	2015	W00022a	MH020264						
Bruchus viciae	Bulgaria	Twyrdica	2015	W00022b	MH020265						
Bruchus viciae	Bulgaria	Paweł Banja	2015	W00022c	MH020266						
Caccobius schreberi	Romania	Tanacu	2016	W00094a							
Caccobius schreberi	Poland	Wielopole	2015	W00094b	MH020304						
Calathus fuscipes	Romania	Moldova	2016	W00213a	MH020379						
Calathus fuscipes	Poland	Żerosławice	2016	W00213b	MH020380						
Calathus fuscipes	Bulgaria	Karlovo	2015	W00213c	MH020381						
Calvia quatuordecimpunctata	Austria	Porrau	2015	W00181a							
Calvia quatuordecimpunctata	Poland	Białowieża Forest	2014	W00181b	MH115503						
Cantharis livida	Slovakia	Turydica	2016	W00047a	MH115526						
Cantharis livida	Romania	Brezoi	2015	W00047b	+						
Cantharis nigricans	Poland	Jarmuta Mt.	2014	W00043a	MH115525						
Cantharis nigricans	Poland	Kędzierzyn-Koźle	2014	W00043b							
Cantharis nigricans	Poland	Krempachy	2014	W00043c							
Cantharis rustica	Poland	Dursztyn	2014	W00046a	MH115524						
Cantharis rustica	Romania	Brezoi	2015	W00046b	MH115523						
Carabus cancellatus	Poland	Sułów	2017	W00145a	MH020417						
Carabus cancellatus	Poland	Zamość	2016	W00145b	MH020418						
Carabus coriaceus	Poland	Chełm	2015	W00207a	MH020374						
Carabus coriaceus	Poland	Chełm	2015	W00207b							
Carabus coriaceus	Poland	Buczków	2014	W00207c	MH020375						
Carabus coriaceus	Poland	Białowieża Forest	2014	W00207d							
Carabus glabratus	Slovakia	Bratislava	2016	W00209a	MH020371						
Carabus glabratus	Poland	Wałsza river valley	2017	W00209b	MH020372	MG987874					
Carabus glabratus	Poland	Białowieża Forest	2014	W00209c							
Carabus granulatus	Poland	Zamość	2016	W00144a	MH020376						
Carabus granulatus	Poland	Graziowa	2017	W00144b	MH020377						
Carabus granulatus	Poland	Białowieża Forest	2014	W00144c							
Carabus granulatus	Poland	Lublin	2015	W00144d							

Carabus hortensis	Poland	Białowieża Forest	2014	W00142a	+						
Carabus hortensis	Poland	Odolion ad Ciechocinek	2016	W00142b	MH020368						
Carabus hortensis	Poland	Lublin	2015	W00142c	MH020370						
Carabus hortensis	Slovakia	Bratislava	2016	W00142d	MH020369						
Carabus nemoralis	Poland	Lublin	2015	W00143a	MH020373						
Carabus nemoralis	Poland	Chełm	2015	W00143b	+						
Carabus nemoralis	Poland	Białowieża Forest	2014	W00143c							
Carabus nemoralis	Slovakia	Bratislava	2016	W00143d							
Carabus violaceus	Poland	Buczków	2014	W00210a							
Carabus violaceus	Slovakia	Bratislava	2016	W00210b	MH020378						
Cassida viridis	Poland	Łapsze Niżne	2014	W00023a	MH115476						
Cassida viridis	Poland	Zdzieszowice	2015	W00023b	MH115478	MG987895	+	+	+	+	+
Cassida viridis	Bulgaria	Cziflik	2015	W00023c	MH115477	MG987896	MG987936		MG987736	MG987696	MG987984
Catapion jaffense	Poland	Białowieża Forest	2015	W00305a	MH218851	MG987830	MG987921	MG987667		MG987703	MG988002
Catapion jaffense	Poland	Przemyśl	2014	W00305b	MH218852						
Catapion koestlini	Poland	Białowieża Forest	2015	W00345a	+						
Catapion koestlini	Poland	Białowieża Forest	2015	W00345b	+						
Catapion meieri	Poland	Jaroszów	2014	W00307a	MH218855	MG987865	MG987920		MG987724	MG987673	
Catapion meieri	Poland	Przemyśl	2014	W00307b	MH218856						
Catapion pubescens	Poland	Jawor	2016	W00306a	MH218849						
Catapion pubescens	Poland	Winnik	2015	W00306b	MH218850						
Catapion seniculus	Poland	Mielnik	2014	W00304a	MH218853	MG987869	MG987917	MG987661	MG987725		
Catapion seniculus	Poland	Chełmowa Mt.	2014	W00304b	MH218854						
Centricnemus leucogrammus	Ukraine	Kasova Mt.	2014	W00327a	+						
Centricnemus leucogrammus	Czechia	Pouzdrany	2014	W00327b	+						
Centricnemus leucogrammus	Poland	Racławice	2015	W00327c	KP306881						
Cetonia aurata	Poland	Makowa	2014	W00070b	MH020442						
Cetonia aurata	Bulgaria	Anevo	2015	W00070d							
Cetonia aurata	Romania	Didesti	2015	W00070e							
Cetonia aurata	Austria	Hundsheim	2015	W00070i							
Ceutorhynchus obstrictus	Poland	Gruczno	2010	W00001c	MH218861	+	+	+	+	+	+
Ceutorhynchus obstrictus	Poland	Ślesin	2010	W00001d	MH218862	+	+	+	+	+	+
Ceutorhynchus obstrictus	Poland	Ślesin	2010	W00001e	MH218863	+	+	+	+	+	+
Ceutorhynchus obstrictus	Slovakia	Čenkov	2012	W00001f	MH218864	+	+	+	+	+	+
Ceutorhynchus obstrictus	Hungary	Váckisújfalu	2012	W00001g	MH218865	+	+	+	+	+	+
Ceutorhynchus obstrictus	Hungary	Váckisújfalu	2012	W00001h	MH218866	+	+	+	+	+	+
Ceutorhynchus typhae	Poland	Skotniki	2015	W00292a	MH115487						
Ceutorhynchus typhae	Bulgaria	Anevo	2015	W00292b	MH115486	MG987816	MG987927	MG987631	MG987737	MG987694	MG987976
Ceutorhynchus typhae	Poland	Kędzierzyn-Koźle	2014	W00292c	+	MG987815	MG987962	MG987627	MG987738	MG987690	MG987988
Cheilotoma musciformis	Ukraine	Kasova Mt.	2014	W00317a	JQ015256						
Cheilotoma musciformis	Slovakia	Devinska Kobyla	2014	W00317b	JQ015253						
Cheilotoma musciformis	Poland	Morsko	2015	W00317c	JQ015254						
Chilocorus renipustulatus	Poland	Szyk	2014	W00183a							
Chilocorus renipustulatus	Poland	Białowieża Forest	2015	W00183b	MH020498						
Chlaenius nitidulus	Poland	Paziowa Mt.	2015	W00221a	MH020404						
Chlaenius tibialis	Poland	Podrzecze	2016	W00220a	MH020401						
Chlaenius tibialis	Poland	Lipnica Wielka	2017	W00220b	MH020402						
Chlaenius tibialis	Poland	Długopole	2016	W00220c	MH020403						
Chrysanthia geniculata	Poland	Sztum	2014	W00111a							
Chrysanthia geniculata	Poland	Sztum	2014	W00111b	MH020339						
Chrysolina herbacea	Poland	Dursztyn	2015	W00024a	MH115557	MG987807		+			+
Chrysolina herbacea	Bulgaria	Karware	2015	W00024b		MG987808	MG987924	MG987635	MG987746	MG987697	MG987985
Chrysolina herbacea	Bulgaria	Cziflik	2015	W00024c	MH115558	MG987809	MG987925	+	MG987747	MG987698	MG987986
Chrysolina polita	Poland	Dursztyn	2014	W00025a	MH115554						
Chrysolina polita	Poland	Krempachy	2014	W00025b	MH115556						
Chrysolina polita	Poland	Jarmuta Mt.	2014	W00025c	MH115555						
Chrysolina varians	Poland	Krempachy	2014	W00039a	MH115553						
Chrysolina varians	Poland	Wdżar Mt.	2014	W00039b							
Chrysolina varians	Poland	Krempachy	2014	W00039c							
Cicindela hybrida	Slovakia	Andrejovka	2017	W00214a							
Cicindela hybrida	Slovakia	Rokytov	2017	W00214b	MH115497						
Cicindela hybrida	Poland	Domosławice	2017	W00214c	MH115498						
Clerus mutillarius	Austria	Füllersdorf	2015	W00127a	MH020286						
Clerus mutillarius	Austria	Füllersdorf	2015	W00127b	MH020287						
Clerus mutillarius	Austria	Füllersdorf	2015	W00127c							
Clivina collaris	Poland	Iskań	2014	W00231a	MH020432						
Coccinella quinquepunctata	Poland	Białowieża Forest	2015	W00130a	MH020503						
Coccinella quinquepunctata	Poland	Białowieża Forest	2015	W00130b	MH020504						
Coccinella quinquepunctata	Poland	Urszulin	2016	W00130c	+						
Coccinella quinquepunctata	Slovakia	Studienka	2016	W00130d							
Coccinella septempunctata	Poland	Urszulin	2016	W00135a	MH020505						
Coccinella septempunctata	Poland	Urszulin	2016	W00135b	MH020506						
Coccinella septempunctata	Poland	Rogalin	2016	W00135c							
Coccinella septempunctata	Slovakia	Sampor	2017	W00135d							
Coccinula quatuordecimpustulata	Slovakia	Sampor	2017	W00170a	MH020510	MG987898					
Coccinula quatuordecimpustulata	Slovakia	Pernek	2016	W00170b		MG987899					
Coccinula quatuordecimpustulata	Poland	Urszulin	2016	W00170c	MH020511						
Combocerus glaber	Bulgaria	Kalofer	2015	W00165a	MH115491	MG987894	MG987910		MG987727	MG987672	MG988037
Coraebus elatus	Belarus	Dikoe Marshes	2013	W00332a	JQ303264						
Coraebus elatus	Poland	Kielce	2013	W00332b	JQ303271						
Coraebus elatus	Slovakia	Čenkov	2013	W00332c	JQ303279						
Coraebus elatus	Ukraine	Ustia	2013	W00332c	JQ303266						
Crepidodera aurata	Poland	Krempachy	2014	W00026a	MH020468						
Crepidodera aurata	Poland	Szymiszów	2014	W00026b	MH020469	MG987814		MG987632	MG987767	MG987714	MG988016
Crepidodera aurata	Bulgaria	Balkanec	2015	W00026c	MH020470						
Crioceris asparagi	Poland	Pińczów	2015	W00319a	KY007631						
Crioceris asparagi	Poland	Grabowo	2015	W00319b	JF775780						
Crioceris asparagi	Slovakia	Devin	2014	W00319c	JF775779						
Crioceris quatuordecimpunctata	Poland	Grabowo	2015	W00049a	JQ015218		KY316086	KY316064	JQ015250	KY316671	JQ015239
Crioceris quatuordecimpunctata	Slovakia	Devin	2014	W00049b	JQ015216						
Crioceris quatuordecimpunctata	Ukraine	Lomachyntsi	2014	W00049c	JF775788	+	KY316086				
Crioceris quatuordecimpunctata	Poland	Skowronno	2015	W00049d	JQ015217		KY316086	KY316065	JQ015246	KY316672	JQ015238
Crioceris quinquepunctata	Czechia	Pouzdřany	2014	W00337a	KC123334		KY316088	KY316066	KC123341	KY316973	KY317680

Crioceris quinquepunctata	Ukraine	Lomachyntsi	2014	W00337b	KC123331		KY316088	KY316067	KC123344	KY317074	JQ015240
Crioceris quinquepunctata	Ukraine	Lomachyntsi	2014	W00337c	KC123330		KY316088				
Crioceris quinquepunctata	Czechia	Pouzdřany	2014	W00337d	KC123333		KY316088				
Cryptocephalus bameuli	Ukraine	Ustia	2013	W00321a	KJ765907						
Cryptocephalus bameuli	Czechia	Sedlec	2015	W00321b	KJ765904						
Cryptocephalus bameuli	Poland	Tunel	2016	W00321c	KJ765899						
Cryptocephalus bipunctatus	Poland	Radzionków	2014	W00027a	MH271193						
Cryptocephalus bipunctatus	Poland	Ligota Dolna	2015	W00027b							
Cryptocephalus bipunctatus	Bulgaria	Paweł Banja	2015	W00027c	MH271194						
Cryptocephalus flavipes	Poland	Pieniny Mts	2015	W00322a	KJ765879						
Cryptocephalus flavipes	Poland	Pieniny Mts	2015	W00322b	KJ765880						
Cryptocephalus flavipes	Poland	Pieniny Mts	2015	W00322c	KJ765881						
Cryptocephalus hypochoeridis	Poland	Pińczów	2015	W00028a	KJ765925	MG987825	MG987942	+	+		MG987970
Cryptocephalus hypochoeridis	Poland	Morsko	2015	W00028b		MG987826	MG987943	MG987650	+		MG987968
Cryptocephalus moraei	Poland	Szymiszów	2014	W00029a	KJ765927						
Cryptocephalus moraei	Poland	Sromowce Wyżne	2014	W00029b							
Cryptocephalus moraei	Bulgaria	Paweł Banja	2015	W00029c	MH271195						
Cryptocephalus quadripustulatus	Poland	Morsko	2014	W00323a	+						
Cryptocephalus sericeus	Poland	Szymiszów	2014	W00030a							
Cryptocephalus sericeus	Poland	Krempachy	2014	W00030b							
Cryptocephalus sericeus	Poland	Ligota Dolna	2015	W00030c							
Cryptocephalus signatifrons	Poland	Janowiec	2014	W00324a							
Cryptocephalus violaceus	Poland	Chotel Czerwony	2015	W00326a	KP306801						
Cryptocephalus violaceus	Ukraine	Ustia	2013	W00326b	KJ765872						
Cyanapion columbinum	Poland	Pińczów	2015	W00346a	KY617917						
Cyanapion columbinum	Poland	Jaroszów	2015	W00346b	KY617916						
Cyanapion columbinum	Poland	Przemyśl	2015	W00346c	KY617915						
Cyanapion afer	Poland	Przemyśl	2015	W00335a	KY617908		KY618005	KY618022	KY618039	KY618054	KY618072
Cyanapion afer	Poland	Wojcieszów	2015	W00335b	KY617907		KY618003	KY618020	KY618037	KY618053	KY618070
Cyanapion afer	Poland	Wrocław	2015	W00335c	KY617905		KY618002	KY618019	KY618036	KY618053	KY618069
Cyanapion gnarum	Poland	Białowieża Forest	2015	W00333a	KY617899						
Cyanapion gnarum	Poland	Góra Winnik Mt.	2015	W00333b	KY617898						
Cyanapion gnarum	Poland	Wojcieszów	2015	W00333c	KY617902						
Cyanapion gyllenhalii	Poland	Korzecko	2015	W00334a	KY617914		KY618008	KY618028	KY618042	KY618068	KY618075
Cyanapion gyllenhalii	Poland	Wojcieszów	2015	W00334b	KY617913		KY618010	KY618027	KY618044	KY618060	KY618077
Cyanapion gyllenhalii	Poland	Wrocław	2015	W00334c	KY617911		KY618011	KY618025	KY618045	KY618061	KY618078
Cyanapion platalea	Poland	Biebrza river valley	2015	W00338a	KY617895						
Cyanapion platalea	Poland	Chęciny	2015	W00338b	KY617897						
Cyanapion platalea	Poland	Skowronno	2015	W00338c	KY617893						
Cyanapion spencii	Poland	Białowieża Forest	2015	W00336a	KY617924		KY618014	KY618031	KY618048	KY618064	KY618081
Cyanapion spencii	Poland	Pińczów	2015	W00336b	KY617923		KY618013	KY618032	KY618047	KY618063	KY618080
Cyanapion spencii	Poland	Stary Załom	2015	W00336c	KY617921		KY618012	KY618029	KY618046	KY618062	KY618079
Dacne bipustulata	Poland	Kolonowskie	2016	W00344a	MH020481						
Dacne rufifrons	Bulgaria	Paweł Banja	2015	W00164a	MH020482	MG987783	MG987947	MG987629	MG987762	MG987711	MG988010
Dacne rufifrons	Bulgaria	Paweł Banja	2015	W00164b	MH020484	MG987784	MG987948	MG987630	MG987763	MG987712	MG987969
Dacne rufifrons	Bulgaria	Paweł Banja	2015	W00164c	MH020483	MG987785	MG987949		MG987764	MG987713	MG988011
Dalopius marginatus	Poland	Białowieża Forest	2015	W00281a	+						
Dascillus cervinus	Poland	Białowieża Forest	2016	W00242a	*DQ221982*						
Dascillus cervinus	Poland	Białowieża Forest	2016	W00242b	+						
Dasytes plumbeus	Poland	Białowieża Forest	2016	W00109a	MH020322						
Dasytes plumbeus	Poland	Białowieża Forest	2016	W00109b	MH020323						
Dasytes plumbeus	Poland	Białowieża Forest	2016	W00109c							
Deporaus betulae	Poland	Białowieża Forest	2016	W00272a	MH115545						
Deporaus betulae	Poland	Białowieża Forest	2016	W00272b	MH115546						
Dermestes lardarius	Poland	Białowieża Forest	2016	W00243a	MH115494						
Dermestes lardarius	Poland	Białowieża Forest	2016	W00243b							
Derocrepis rufipes	Poland	Krempachy	2014	W00040a	MH020467						
Derocrepis rufipes	Poland	Cisowa Skała	2014	W00040b							
Derocrepis rufipes	Poland	Krempachy	2014	W00040c							
Diaperis boleti	Poland	Kępie	2017	W00189a	MH020449						
Diaperis boleti	Poland	Kępie	2017	W00189b							
Dinoptera collaris	Poland	Jarmuta Mt.	2014	W00100a	MH115482						
Dinoptera collaris	Poland	Grzybowa	2017	W00100b	MH115573						
Dinoptera collaris	Poland	Białowieża Forest	2016	W00100c							
Dolichosoma lineare	Poland	Tymianki ad Ciechanowiec	2016	W00246a	MH020324						
Dolichosoma lineare	Poland	Miszek	2016	W00246b	MH020325						
Dorcus parallelipipedus	Poland	Rogalin	2016	W00107a	MH115537						
Dorcus parallelipipedus	Poland	Rogalin	2016	W00107b	MH115538						
Dorcus parallelipipedus	Poland	Rusków	2016	W00107c							
Dromius agilis	Poland	Lublin	2015	W00230a	MH020407	MG987835	MG987952	MG987633	MG987769	MG987692	MG987975
Dryocoetes autographus	Poland	Łapsze Niżne	2014	W00113a	MH115483						
Dryocoetes autographus	Poland	Łapsze Niżne	2014	W00113b	MH115484						
Dryocoetes autographus	Poland	Łapsze Niżne	2014	W00113c							
Drypta dentata	Poland	Okale	2015	W00212a	MH115499						
Dyschirius digitatus	Poland	Iskań	2017	W00204a	MH020473						
Dyschirius digitatus	Poland	Posada Rybotycka	2017	W00204b	MH020474						
Elaphropus quadrisignatus	Poland	Nowa Biała	2014	W00206a	MH020391						
Elaphropus quadrisignatus	Poland	Iskań	2017	W00206b	MH020392						
Elaphrus aureus	Poland	Tolkmicko	2017	W00259a	*KU918463*						
Elaphrus riparius	Poland	Niebrzegów	2015	W00141a	MH020433						
Elaphrus riparius	Poland	Iskań	2017	W00141b	MH020434						
Elaphrus riparius	Slovakia	Andrejovka	2017	W00141e							
Etorofus pubescens	Poland	Białowieża Forest	2016	W00266a	MH020330	MG987851	MG987953	MG987639	MG987752	MG987693	MG988007
Etorofus pubescens	Poland	Białowieża Forest	2016	W00266b	MH020331	MG987850	MG987954	MG987640	MG987753	+	MG988008
Euoniticellus fulvus	Poland	Wielopole	2015	W00088c	MH020543						
Euoniticellus fulvus	Romania	Islaz	2016	W00088d	MH020544						
Euoniticellus fulvus	Bulgaria	Anevo	2016	W00088e	MH020545						
Eusomus ovulum	Poland	Pińczów	2015	W00339a	KP306855	GU111693	KU341558	KU341554	GU111709	KU341556	KU341557
Eusomus ovulum	Bulgaria	Anevo	2016	W00339b	KU341544	GU111693	KU341558	KU341554	GU111709	KU341556	KU341557
Eusomus ovulum	Ukraine	Ustia	2013	W00339c	KU341542	GU111693	KU341558	KU341554	GU111709	KU341556	KU341557
Eusomus ovulum	Austria	Füllersdorf	2016	W00339d	KU341529	GU111693	KU341558	KU341554	GU111709	KU341556	KU341557
Eusomus ovulum	Hungary	Lénárddaróc	2014	W00339e	KU341532	GU111693	KU341558	KU341554	GU111709	KU341556	KU341557
Eutrichapion melancholicum	Poland	Wojtkówka	2016	W00308a	MH218859						

Eutrichapion melancholicum	Poland	Przemyśl	2014	W00308b	MH218860						
Eutrichapion viciae	Poland	Korzecko	2015	W00309a	MH218857						
Eutrichapion viciae	Poland	Korzecko	2015	W00309b	MH218858						
Exochomus quadripustulatus	Slovakia	Sampor	2017	W00169a	MH115507						
Exochomus quadripustulatus	Slovakia	Studienka	2016	W00169b	MH115508						
Exochomus quadripustulatus	Slovakia	Pernek	2016	W00169c							
Exochomus quadripustulatus	Poland	Miszek	2016	W00169d	+						
Galeruca tanaceti	Bulgaria	Karlovo	2015	W00041a	MH020463						
Galeruca tanaceti	Bulgaria	Karlovo	2015	W00041b							
Galeruca tanaceti	Bulgaria	Paweł Banja	2015	W00041c							
Galerucella tenella	Poland	Wdżar Mt.	2014	W00042a	MH020460						
Galerucella tenella	Poland	Łapsze Niżne	2014	W00042b							
Galerucella tenella	Poland	Dursztyn	2014	W00042c							
Gnaptor spinimanus	Bulgaria	Karnare	2015	W00068a	<u>*KX388742*</u>						
Gnaptor spinimanus	Romania	Didesti	2016	W00068b							
Gnaptor spinimanus	Romania	Tanacu	2016	W00068c							
Gnaptor spinimanus	Bulgaria	Anevo	2015	W00068d							
Harpalus affinis	Poland	Mogielica Mt.	2015	W00216a							
Harpalus affinis	Romania	Păscoaia	2016	W00216b							
Harpalus rufipes	Poland	Chełm	2015	W00199a	MH020398						
Harpalus rufipes	Poland	Sułów	2017	W00199b	MH020399						
Harpalus rufipes	Poland	Okale	2015	W00199c	+						
Hippodamia variegata	Slovakia	Horná Mičiná	2016	W00175a	MH115504						
Hippodamia variegata	Slovakia	Horná Mičiná	2016	W00175b							
Hylobius abietis	Bulgaria	Paweł Banja	2015	W00294a	MH115471						
Hylobius abietis	Bulgaria	Elena	2015	W00294b		MG987817	MG987928	MG987647	MG987745		MG987971
Hylobius abietis	Romania	Făgăraş Mts.	2015	W00294c	+						
Hylotrupes bajulus	Poland	Białowieża Forest	2016	W00264a	MH020455						
Hylotrupes bajulus	Poland	Białowieża Forest	2016	W00264b	MH020456						
Ips typographus	Poland	Białowieża Forest	2016	W00114a	MH115481						
Ips typographus	Poland	Białowieża Forest	2016	W00114b	+						
Ips typographus	Slovakia	Cisty	2017	W00114c							
Kateretes pedicularius	Poland	Kolonowskie	2015	W00058a	MH020488						
Kateretes pedicularius	Poland	Nowa Wieś Królówka	2016	W00058b	MH020489						
Kateretes pedicularius	Poland	Nowa Wieś Królówka	2016	W00058c							
Lagria hirta	Slovakia	Kremnicke Vrchy	2016	W00156a	MH115560	MG987875					
Lagria hirta	Poland	Nałęczów	2015	W00156b							
Lagria hirta	Poland	Białowieża Forest	2016	W00156c		MG987876					
Litargus connexus	Poland	Kolonowskie	2015	W00288a	MH020363						
Lixus filiformis	Bulgaria	Stara Planina Mts.	2015	W00293a							
Lixus filiformis	Bulgaria	Anevo	2015	W00293b	MH115475						
Lixus filiformis	Bulgaria	Anevo	2015	W00293c							
Lochmaea caprea	Poland	Niedzica	2014	W00051a	+						
Lochmaea caprea	Romania	Tau Bistrica	2015	W00051b							
Lochmaea caprea	Croatia	Pomena	2016	W00051c							
Longitarsus exsoletus	Poland	Szymiszów	2014	W00031a	MH020464						
Longitarsus exsoletus	Poland	Krempachy	2014	W00031b							
Longitarsus exsoletus	Poland	Cisowa Skała	2014	W00031c							
Longitarsus nasturtii	Poland	Krempachy	2014	W00032a							
Longitarsus nasturtii	Poland	Ligota Dolna	2015	W00032b							
Longitarsus nasturtii	Poland	Łapsze Niżne	2014	W00032c	MH271196						
Loricera pilicornis	Poland	Lublin	2015	W00202a	MH020437						
Loricera pilicornis	Poland	Lublin	2015	W00202b	MH020438						
Loricera pilicornis	Poland	Niebrzegów	2015	W00202c	MH020439						
Luperus flavipes	Poland	Cisowa Skała	2014	W00033a	MH020461	MG987836	MG987957	MG987651	MG987757	MG987707	MG988020
Luperus flavipes	Poland	Szymiszów	2014	W00033b		MG987837	MG987958	MG987652	MG987758	MG987708	MG988021
Luperus flavipes	Poland	Krempachy	2014	W00033c		MG987840			MG987768		MG988015
Luperus luperus	Poland	Wdżar Mt.	2014	W00034a	MH020462	MG987838	MG987959		MG987759	MG987709	MG988018
Luperus luperus	Poland	Krempachy	2014	W00034b		MG987839					
Luperus luperus	Poland	Ligota Dolna	2015	W00034c		+	MG987960		MG987760	MG987710	MG988019
Lygistopterus sanguineus	Poland	Rogalin	2016	W00137a	MH115527						
Lygistopterus sanguineus	Poland	Białowieża Forest	2016	W00137b	MH115528						
Lygistopterus sanguineus	Poland	Knyszyn Forest	2016	W00137c							
Mecinus pascuorum	Poland	Murownia	2017	W00302a	+			MG987666	MG987733		MG988039
Mecinus pascuorum	Poland	Murownia	2017	W00302b	+						
Mecinus pascuorum	Bulgaria	Anevo	2015	W00302c							
Melolontha melolontha	Romania	Brezoi	2015	W00060a	MH115534						
Melolontha melolontha	Romania	Brezoi	2015	W00060b							
Melolontha melolontha	Romania	Brezoi	2015	W00060c	+						
Metacantharis discoidea	Poland	Dursztyn	2014	W00290a	+						
Metacantharis discoidea	Poland	Dursztyn	2014	W00290b	+						
Metacantharis discoidea	Poland	Dursztyn	2014	W00290c							
Monochamus galloprovincialis	Poland	Kampinos	2015	W00316a	MH271199						
Monochamus galloprovincialis	Poland	Sieraków	2016	W00316b	MH271200						
Monochamus sartor sartor	Poland	Komańcza	2015	W00314a	MF371185	MG987822	MF405511	MF405524	MF405536	MF405530	MF405543
Monochamus sartor sartor	Poland	Baligród	2015	W00314b	MF371196		MF405510	MF405525	MF405537	MF405531	MF405544
Monochamus sartor sartor	Poland	Pieniny Mts	2015	W00314c	MF371201		MF405512	MF405526	MF405538	MF405532	MF405545
Monochamus sartor urussovii	Poland	Białowieża Forest	2016	W00313a	MF327411	MG987827	MF405508	MF405521	MF405533	MF405527	MF405540
Monochamus sartor urussovii	Poland	Knyszyn Forest	2015	W00313b	MF327421		MF405509	MF405522	MF405534	MF405528	MF405541
Monochamus sartor urussovii	Poland	Augustów Forest	2016	W00313c	MF327418		MF405507	MF405523	MF405535	MF405529	MF405542
Monochamus sutor	Poland	Borecka Forest	2015	W00315a	MH271201						
Monochamus sutor	Poland	Borecka Forest	2015	W00315b	MH271202						
Mordella brachyura	Poland	Chrusty	2015	W00108a	MH115567						
Mordella brachyura	Poland	Chrusty	2015	W00108b	+						
Mordella brachyura	Poland	Chrusty	2015	W00108c	+						
Mordella holomelaena	Poland	Niepołomice Forest	2016	W00110a	MH115566						
Mordella holomelaena	Poland	Niepołomice Forest	2016	W00110b	+						
Mordella holomelaena	Poland	Niepołomice Forest	2016	W00110c							
Musaria affinis	Poland	Ispina	2017	W00208a	MH020471						
Musaria affinis	Poland	Widniówka	2017	W00208b							
Musaria affinis	Poland	Widniówka	2017	W00208c	MH020472						
Mycetophagus ater	Poland	Kępie	2017	W00186a	MH020297						
Mycetophagus multipunctatus	Poland	Kępie	2017	W00185a	MH020296						

Mycetophagus piceus	Poland	Kolonowskie	2015	W00287a	MH020301						
Mycetophagus quadripustulatus	Poland	Kępie	2017	W00187a	MH020298						
Mycetophagus quadripustulatus	Poland	Kępie	2017	W00187b	MH020299						
Myrrha octodecimguttata	Slovakia	Sampor	2017	W00171a	MH020499	MG987906		MG987669			
Myrrha octodecimguttata	Slovakia	Studienka	2016	W00171b	MH020501						
Myrrha octodecimguttata	Slovakia	Sampor	2017	W00171c	MH020500						
Neatus picipes	Poland	Wyliny Ruś	2015	W00285a							
Nebria picicornis	Poland	Długopole	2016	W00217a	MH020411	MG987833	MG987938	MG987648	MG987741	MG987700	MG987987
Neocoenorhinus germanicus	Poland	Kajasówka	2013	W00012a	MH115542	MG987872	MG987909	+	MG987719	MG987670	MG988029
Neocoenorhinus germanicus	Poland	Skotniki	2014	W00012b	MH115544						
Neocoenorhinus germanicus	Hungary	Lénárddaróc	2014	W00012c	MH115543						
Nedyus quadrimaculatus	Poland	Dursztyn	2014	W00291a	MH115488	MG987788	MG987929	MG987644	MG987742	MG987684	MG987989
Nedyus quadrimaculatus	Poland	Kędzierzyn-Koźle	2014	W00291b	+	MG987790	MG987930	MG987645	MG987743	MG987685	MG987990
Nedyus quadrimaculatus	Bulgaria	Anevo	2015	W00291c		MG987789					MG987991
Nicrophorus vespilloides	Poland	Białowieża Forest	2016	W00159a	MH115513	MG987883					
Nicrophorus vespilloides	Poland	Białowieża Forest	2016	W00159b	MH115515	MG987884					
Nicrophorus vespilloides	Poland	Żmiąca	2016	W00159c	MH115514	MG987885					
Nivellia sanguinosa	Poland	Jarmuta Mt.	2014	W00101a	MH020294						
Nivellia sanguinosa	Poland	Kluszkowce	2015	W00101b	MH020295						
Nivellia sanguinosa	Poland	Grzybowa	2017	W00101c							
Notiophilus biguttatus	Poland	Szyk	2014	W00200a	MH020435						
Notiophilus biguttatus	Poland	Żmiąca	2016	W00200b	MH020436						
Notoxus monoceros	Poland	Urszulin	2016	W00157a	MH020272	MG987877		MG987668	+		MG988048
Notoxus monoceros	Poland	Urszulin	2016	W00157b	MH020273	MG987878		MG987649	+		
Notoxus monoceros	Poland	Urszulin	2016	W00157c	MH020274	MG987879		+	+		
Obrium brunneum	Poland	Jarmuta Mt.	2014	W00099a	MH115575						
Ocypus olens	Poland	Odolion ad Ciechocinek	2016	W00252a	MH115529						
Oedemera femorata	Poland	Knyszyn Forest	2016	W00057a	MH020333						
Oedemera femorata	Poland	Białowieża Forest	2016	W00057b	MH020334						
Oedemera femorata	Poland	Białowieża Forest	2016	W00057c	MH020335						
Oedemera flavipes	Poland	Sztum	2014	W00063a	MH020336						
Oedemera flavipes	Greece	Akropol	2016	W00063b	MH020337						
Oedemera flavipes	Greece	Akropol	2016	W00063c	MH020338						
Oenopia conglobata	Poland	Urszulin	2016	W00184a	MH020502						
Oiceoptoma thoracica	Poland	Jarmuta Mt.	2014	W00155a	*KU918637.1*	MG987908				MG987702	MG988001
Omalisus fontisbellaquaei	Poland	Cisowa Skała	2014	W00139a							
Omalisus fontisbellaquaei	Poland	Cisowa Skała	2014	W00139b	MH115572						
Omalisus fontisbellaquaei	Poland	Niedzica	2014	W00139c	+						
Omonadus floralis	Poland	Białowieża Forest	2016	W00278a	*KM449287*						
Omophron limbatum	Poland	Sortowice	2015	W00215a	MH020430						
Omophron limbatum	Czechia	Kopytov	2016	W00215b	MH020431						
Onthophagus fracticornis	Poland	Sromowce Niżne	2014	W00085a							
Onthophagus fracticornis	Bulgaria	Balkanec	2015	W00085e	MH020366	MG987843					MG988024
Onthophagus fracticornis	Poland	Mogielica Mt.	2015	W00085j		MG987844					
Onthophagus ovatus	Poland	Sromowce Niżne	2014	W00121a	+						
Onthophagus ovatus	Bulgaria	Turia	2015	W00121b	MH020367						
Onthophagus ovatus	Austria	Füllersdorf	2016	W00121c							
Onthophagus ruficapillus	Romania	Tanacu	2016	W00086c	MH020535	MG987799					MG988025
Onthophagus ruficapillus	Romania	Tanacu	2016	W00086d	+	MG987800					
Onthophagus ruficapillus	Bulgaria	Kalofer	2015	W00086g	+	MG987801					MG988026
Onthophagus ruficapillus	Bulgaria	Kalofer	2015	W00086h	+	MG987802					
Onthophagus ruficapillus	Romania	Didesti	2016	W00086i	+	MG987804					MG988027
Onthophagus ruficapillus	Bulgaria	Anevo	2015	W00086j	+	MG987803					MG988028
Onthophagus similis	Bulgaria	Kalofer	2015	W00092a	MH020540	MG987791					
Onthophagus similis	Austria	Füllersdorf	2016	W00092b	MH020542						MG988023
Onthophagus similis	Poland	Wielopole	2015	W00092c							
Onthophagus similis	Romania	Fenes	2016	W00092d	MH020541						
Onthophagus taurus	Bulgaria	Kalofer	2015	W00093a	MH020531	MG987792					MG987994
Onthophagus taurus	Bulgaria	Kalofer	2015	W00093b	MH020533	+					MG987999
Onthophagus taurus	Bulgaria	Kalofer	2015	W00093d	+	MG987797					
Onthophagus taurus	Poland	Wielopole	2015	W00093e	+	MG987793					MG987995
Onthophagus taurus	Poland	Wielopole	2015	W00093g	+	MG987794					MG987998
Onthophagus taurus	Poland	Wielopole	2015	W00093h	+	MG987798					MG988000
Onthophagus taurus	Poland	Wielopole	2015	W00093i	+	MG987795					MG987996
Onthophagus taurus	Poland	Wielopole	2015	W00093j	+	MG987796					MG987997
Ophonus laticollis	Poland	Chełm	2015	W00228a	MH020397						
Otiorhynchus perdix	Romania	Făgăraș Mts.	2015	W00298a	MH115571						
Otiorhynchus perdix	Romania	Tau Bistrica	2015	W00298b							
Otiorhynchus perdix	Romania	Cascada Capra	2016	W00298c	+						
Otiorhynchus riessi	Romania	Cascada Capra	2016	W00297a	*KR481561*						
Otiorhynchus riessi	Romania	Făgăraș Mts.	2015	W00297b	+						
Otiorhynchus riessi	Romania	Făgăraș Mts.	2015	W00297c							
Oulema gallaeciana	Poland	Groszowice	2015	W00053a	MH020546						
Oulema gallaeciana	Romania	Didesti	2016	W00053b							
Oulema gallaeciana	Bulgaria	Anevo	2015	W00053c	MH020547						
Oxymirus cursor	Poland	Białowieża Forest	2016	W00283a	MH020332						
Oxythyrea funesta	Poland	Koryciny	2015	W00106a	MH020444						
Oxythyrea funesta	Poland	Ligota Dolna	2015	W00106b	MH020445						
Oxythyrea funesta	Poland	Kolonowskie	2015	W00106c							
Pachyta quadrimaculata	Poland	Białowieża Forest	2016	W00265a	MH020284				MG987772		MG988050
Pachyta quadrimaculata	Poland	Białowieża Forest	2017	W00265b	MH020285				MG987773		MG988051
Pachytodes cerambyciformis	Poland	Białowieża Forest	2017	W00080a							
Pachytodes cerambyciformis	Poland	Grajów	2017	W00080b	MH115502						
Paederidus rubrothoracicus	Poland	Domosławice	2017	W00254a	MF170709						
Paederidus rubrothoracicus	Romania	Moldova	2016	W00254b	MF170711						
Paederidus rubrothoracicus	Poland	Fałkowice	2017	W00254c	MF170708						
Paederidus rubrothoracicus	Ukraine	Pidmostychi	2016	W00254d	MF170710						
Paederidus ruficollis	Poland	Fałkowice	2017	W00255b	MF170701		MF170754	MF170768	MF170782	MF170810	MF170796
Paederidus ruficollis	Poland	Domosławice	2017	W00255c	MF170702		MF170755	MF170769	MF170783	MF170811	MF170797
Paederus caligatus	Czechia	Kopytov	2016	W00180a	MH115549						
Paederus limnophilus	Poland	Domosławice	2017	W00253a	MF170698		MF170753	MF170767	MF170781	MF170809	MF170795
Paederus limnophilus	Romania	Moldova	2016	W00253b	MF170700						
Paederus limnophilus	Poland	Fałkowice	2017	W00253c	MF170697		MF170752	MF170766	MF170780	MF170808	MF170794

Paederus limnophilus	Ukraine	Pidmostychi	2016	W00253d	MF170699						
Paederus littoralis	Poland	Tarnawa	2015	W00173a	MH115550	MG987806	MG987951	+	MG987744	MG987695	MG987980
Paederus riparius	Poland	Podrzecze	2016	W00174a		MG987782	MG987963	+	MG987765	MG987701	MG987992
Paederus riparius	Poland	Fałkowice	2015	W00174b		MG987864	MG987916	+	MG987720	MG987674	MG988038
Paederus riparius	Poland	Rybotycze	2015	W00174c	MH115548						
Paophilus afflatus	Poland	Racławice	2014	W00329a	KP306866	GU111695			GU111699		
Paradromius linearis	Poland	Okńów	2015	W00147a	MH020405						
Paradromius linearis	Poland	Witoszyn	2017	W00147b	MH020406						
Parafoucartia squamulata	Poland	Racławice	2014	W00331a	KP306882	GU111694			GU111697	GQ167647	
Paranchus albipes	Poland	Rybotycze	2015	W00205a	MH020393						
Perileptus areolatus	Poland	Rdzawa	2015	W00203a	*KM452499*						
Perileptus areolatus	Poland	Graziowa	2017	W00203b	+						
Phosphuga atrata	Poland	Brańszczyk	2016	W00250a	*KJ963173*						
Phosphuga atrata	Poland	Białowieża Forest	2016	W00250b	+						
Phyllobius brevis	Poland	Gruczno	2010	W00010a	MH020253						
Phyllobius brevis	Poland	Płutowo	2010	W00010b	MH020254						
Phyllobius brevis	Poland	Ślesin	2010	W00010c	MH020255						
Phyllobius glaucus	Romania	Făgăraş Mts.	2015	W00296a	MH020256	MG987778	MG987939	MG987628	MG987775	MG987686	MG988005
Phyllobius glaucus	Romania	Gros	2016	W00296b	MH020257	MG987779	MG987940		MG987776	MG987687	MG988006
Phyllobius glaucus	Poland	Jarmuta Mt.	2014	W00296c							
Phyllobius maculicornis	Poland	Kowadza	2013	W00017a	MH020258						
Phyllobius maculicornis	Poland	Gruczno	2010	W00017b	MH020259	MG987777	MG987941	MG987626	MG987774	MG987688	MG988004
Phyllobius maculicornis	Poland	Ślesin	2010	W00017c	MH020260						
Phyllopertha horticola	Poland	Dursztyn	2014	W00061a	MH115531						
Phyllopertha horticola	Poland	Wdżar Mt.	2014	W00061b							
Phyllopertha horticola	Poland	Koryciny	2015	W00061c							
Phyllotreta christinae	Poland	Wdżar Mt.	2014	W00054a							
Phyllotreta christinae	Bulgaria	Cziflik	2015	W00054b	MH020552						
Phymatodes testaceus	Poland	Białowieża Forest	2016	W00079a	MH020479						
Phymatodes testaceus	Poland	Rogalin	2016	W00079b	MH020480						
Pidonia lurida	Poland	Jarmuta Mt.	2014	W00097a	MH020291						
Pidonia lurida	Poland	Szyk	2014	W00097b	MH020292						
Pidonia lurida	Poland	Szyk	2014	W00097c	MH020293						
Pityogenes chalcographus	Slovakia	Harmanec	2016	W00168a	MH115485	MG987897			+		
Pityogenes chalcographus	Slovakia	Harmanec	2016	W00168b		MG987888			+		
Pityogenes chalcographus	Slovakia	Harmanec	2016	W00168c							
Plagionotus detritus	Poland	Odolion ad Ciechocinek	2016	W00236a							
Plagionotus detritus	Poland	Białowieża Forest	2016	W00236b	MH115547						
Platynus assimilis	Poland	Tarnawa	2015	W00146a	MH020383						
Platynus assimilis	Poland	Chrusin	2015	W00146b	MH020384						
Platynus assimilis	Poland	Chrusty	2015	W00146c	MH020385						
Poecilus versicolor	Poland	Wielopole	2015	W00233a	MH020400						
Polydrusus inustus	Ukraine	Chartova Gora	2014	W00340a	+	HQ242777			JF304466	JF304464	
Polydrusus inustus	Poland	Kraków	2015	W00340b	KP306886	GU111692			GU111702	GQ167637	
Polydrusus mollis	Bulgaria	Paweł Banja	2015	W00295a							
Polydrusus mollis	Bulgaria	Cziflik	2015	W00295b	+						
Polydrusus mollis	Bulgaria	Cziflik	2015	W00295c	+						
Prionus coriarius	Poland	Ciechanówek	2016	W00115a	MH020282						
Prionus coriarius	Poland	Białowieża Forest	2016	W00115b	MH020283						
Prionus coriarius	Poland	Białowieża Forest	2016	W00115c							
Propylea quatuordecimpunctata	Poland	Białowieża Forest	2016	W00132a	MH020514						
Propylea quatuordecimpunctata	Poland	Białowieża Forest	2016	W00132b	MH020515						
Propylea quatuordecimpunctata	Poland	Urszulin	2016	W00132c							
Pseudomechoris aethiops	Slovakia	Velke vrchy	2011	W00014a	MH115539						
Pseudomechoris aethiops	Poland	Pińczów	2011	W00014b	MH115541						
Pseudomechoris aethiops	Poland	Szaniec	2010	W00014c	MH115540						
Pseudoperapion ergenense	Ukraine	Bilche Zolote	2010	W00015a	MH115569	MG987852	+	+	+	+	+
Pseudoperapion ergenense	Poland	Kąty II	2013	W00015b		MG987853	MG987964	MG987658	+		MG988009
Pseudoperapion ergenense	Ukraine	Ustia	2010	W00015c	MH115568	MG987854	+	MG987665	MG987726	MG987671	MG988030
Pseudovadonia livida	Poland	Białowieża Forest	2016	W00284a	MH020448						
Psyllobora vigintiduopunctata	Poland	Brańszczyk	2016	W00275a	MH020492						
Psyllobora vigintiduopunctata	Poland	Białowieża Forest	2016	W00275b	MH020493		MG987945	MG987654	MG987771		MG988003
Pterostichus melanarius	Poland	Chełm	2015	W00151a	MH020386						
Pterostichus melanarius	Poland	Sułów	2017	W00151b							
Pterostichus melanarius	Poland	Chrusty	2015	W00151c	MH020387						
Pterostichus niger	Poland	Pogwizdów	2016	W00153a	MH020408						
Pterostichus niger	Poland	Chełm	2015	W00153b	MH020409						
Pterostichus niger	Poland	Ispina	2017	W00153c							
Pterostichus niger	Slovakia	Kavaklika	2016	W00153d	MH020410						
Pterostichus oblongopunctatus	Poland	Chełm	2015	W00152a	MH020388						
Pterostichus oblongopunctatus	Poland	Lublin	2015	W00152b	MH020389						
Pterostichus oblongopunctatus	Poland	Kępie	2017	W00152c							
Pterostichus oblongopunctatus	Slovakia	Bratislava	2016	W00152d	MH020390						
Pyrochroa coccinea	Poland	Białowieża Forest	2016	W00260a	MH020355						
Pyrochroa coccinea	Poland	Białowieża Forest	2017	W00260b	MH020356						
Pyrrhidium sanguineum	Austria	Porrau	2015	W00098a							
Pyrrhidium sanguineum	Austria	Füllersdorf	2016	W00098b	MH271197						
Pyrrhidium sanguineum	Austria	Füllersdorf	2016	W00098c	MH271198						
Pytho depressus	Poland	Białowieża Forest	2017	W00249a	MH020446						
Pytho depressus	Poland	Białowieża Forest	2017	W00249b	MH020447						
Rhagium inquisitor	Poland	Białowieża Forest	2016	W00267a	MH020319						
Rhagium inquisitor	Poland	Białowieża Forest	2017	W00267b	MH020320						
Rhagium mordax	Poland	Białowieża Forest	2016	W00268a							
Rhagium mordax	Poland	Białowieża Forest	2016	W00268b	MH020321						
Rhagium sycophanta	Austria	Füllersdorf	2016	W00078a	MH020317						
Rhagium sycophanta	Poland	Rogalin	2016	W00078b	MH020318						
Rhagonycha fulva	Poland	Białowieża Forest	2016	W00235a	MH115520						
Rhagonycha fulva	Poland	Miszek	2016	W00235b	MH115521						
Rhagonycha lignosa	Bulgaria	Hotel Sima	2015	W00045a	MH115519						
Rhagonycha lignosa	Bulgaria	Balkanec	2015	W00045b	MH115522						
Rhagonycha limbata	Poland	Jarmuta Mt.	2014	W00044a	+						
Rhagonycha limbata	Poland	Dursztyn	2014	W00044b	+						
Rhagonycha limbata	Poland	Groszowice	2015	W00044c	MH020466						

Rhinusa tetra	Poland	Morsko	2014	W00301a	+						
Rhinusa tetra	Poland	Kędzierzyn-Koźle	2014	W00301b	+	MG987857					
Rhinusa tetra	Bulgaria	Anevo	2015	W00301c	*LN889539*	MG987858					
Rhyzobius chrysomeloides	Slovakia	Studienka	2016	W00177a	MH115510						
Rhyzobius chrysomeloides	Slovakia	Studienka	2016	W00177b	+						
Rusticoclytus rusticus	Poland	Łążek	2017	W00104b	+						
Rusticoclytus rusticus	Poland	Łążek	2017	W00104c							
Rusticoclytus rusticus	Poland	Białowieża Forest	2016	W00239a	MH020454						
Rutpela maculata	Poland	Tarnawa	2015	W00341a							
Salpingus ruficollis	Poland	Białowieża Forest	2016	W00282a	MH020475						
Saperda scalaris	Poland	Białowieża Forest	2016	W00237a	KM286392						
Saperda scalaris	Poland	Białowieża Forest	2016	W00237b							
Schizotus pectinicornis	Poland	Łęg Zdzieszowski	2017	W00140a	MH020357						
Schizotus pectinicornis	Poland	Białowieża Forest	2016	W00140b	MH020360						
Schizotus pectinicornis	Poland	Białowieża Forest	2016	W00140c							
Sciaphobus rubi	Poland	Racławice	2014	W00330a	*KU913336*	GU111684			GU111700	GQ167639	
Scymnus nigrinus	Slovakia	Studienka	2016	W00178a	MH115509						
Serropalpus barbatus	Poland	Białowieża Forest	2016	W00077a							
Serropalpus barbatus	Poland	Kolonowskie	2015	W00077b	MH020281						
Sibinia pellucens	Ukraine	Bilche Zolote	2010	W00013a	MH115479						
Sibinia pellucens	Poland	Osłowo	2012	W00013b	+	MG987823	MG987922		MG987734	MG987681	MG987967
Sibinia pellucens	Ukraine	Chartova Gora	2010	W00013c	MH115480	MG987824	MG987923	MG987657	MG987735	MG987682	MG987972
Silis nitidula	Poland	Łęg Zdzieszowski	2017	W00289a	MH115516						
Silis nitidula	Poland	Łęg Zdzieszowski	2017	W00289b	MH115518						
Silis nitidula	Poland	Łęg Zdzieszowski	2017	W00289c	MH115517						
Sitona suturalis	Bulgaria	Stara Planina Mts.	2015	W00300a	MH115468						
Sitona suturalis	Poland	Kędzierzyn-Koźle	2014	W00300b							
Sitona suturalis	Romania	Balkanec	2016	W00300c	MH115467	MG987873					
Sitona sulcifrons	Poland	Krempachy	2014	W00299a	MH115469						
Sitona sulcifrons	Poland	Krempachy	2014	W00299b							
Sitona sulcifrons	Bulgaria	Stara Planina Mts.	2015	W00299c	MH115470						
Smaragdina affinis	Slovakia	Devinska Kobyla	2014	W00318a	KP306809						
Spermophagus sericeus	Bulgaria	Karnare	2015	W00052a							
Spermophagus sericeus	Bulgaria	Paweł Banja	2015	W00052b	MH020553						
Spermophagus sericeus	Romania	Didesti	2016	W00052c							
Sphindus dubius	Poland	Kępie	2017	W00190a	MH020450						
Sphindus dubius	Poland	Kępie	2017	W00190b	MH020451						
Spondylis buprestoides	Poland	Odolion ad Ciechocinek	2016	W00270a	MH115501						
Spondylis buprestoides	Poland	Odolion ad Ciechocinek	2016	W00270b							
Staphylinus erythropterus	Poland	Białowieża Forest	2016	W00276a	*KM449869*	MG987904	MG987934	MG987656	MG987750	+	MG987973
Staphylinus erythropterus	Poland	Białowieża Forest	2016	W00276b	+	MG987905	MG987935	MG987653	MG987751	MG987715	MG987993
Stenolophus teutonus	Poland	Rdzawa	2015	W00229a	MH020382						
Stenomax aeneus	Poland	Sułków	2017	W00158a	MH020550	MG987886				+	
Stenomax aeneus	Poland	Sułków	2017	W00158b	+						
Stenomax aeneus	Poland	Białowieża Forest	2016	W00158c	MH020551	MG987887					
Stenurella melanura	Poland	Tymianki ad Ciechocinek	2016	W00271a	MH020458						
Stenurella melanura	Poland	Białowieża Forest	2016	W00271b	MH020459						
Stenurella nigra	Poland	Kępie	2017	W00095a							
Stenurella nigra	Poland	Chrusty	2015	W00095b	MH020344						
Stenurella nigra	Poland	Chrusty	2015	W00095c							
Stenurella nigra	Poland	Białowieża Forest	2016	W00095d							
Stictoleptura maculicornis	Poland	Biebrza river valley	2016	W00238a	MH020520						
Stictoleptura maculicornis	Poland	Białowieża Forest	2016	W00238b	MH020521						
Strangalia attenuata	Poland	Koryciny	2015	W00103a	MH020328						
Strangalia attenuata	Poland	Białowieża Forest	2016	W00103b	MH020329						
Strophosoma capitatum	Czechia	Albrechtice	2014	W00342a	KY347837	KY347847	MF170865	MF170870	MF170875	MF170883	MF170880
Strophosoma capitatum	Germany	Brelinger	2016	W00342b	KY347836						
Strophosoma capitatum	Poland	Tarnawa	2015	W00342c	KY347838	KY347845	MF170867	MF170872	MF170877	MF170887	MF170882
Strophosoma capitatum	Poland	Tyniec	2016	W00342d	KY514492	KY347848	MF170866	MF170871	MF170876	MF170886	MF170881
Strophosoma faber	Poland	Przemyśl	2014	W00328a	KP306863						
Strophosoma melanogrammum	Bulgaria	Gintsi	2015	W00343a	KY347834	KY347844	MF170863	MF170868	MF170873	MF170883	MF170878
Strophosoma melanogrammum	Czechia	Pražmo	2014	W00343b	KY347831	KY347846	MF170864	MF170869	MF170874	MF170884	MF170879
Strophosoma melanogrammum	Germany	Brelinger	2016	W00343c	KY347830	KY347844	MF170863	MF170868	MF170873	MF170883	MF170878
Strophosoma melanogrammum	Romania	Calatele	2016	W00343d	KY347832	KY347844	MF170863	MF170868	MF170873	MF170883	MF170878
Strophosoma melanogrammum	Slovakia	Temeš	2016	W00343e	KY347833	KY347845	MF170863	MF170868	MF170873	MF170883	MF170878
Synapion ebeninum	Poland	Korzecko	2015	W00311a	KY617927						
Synapion ebeninum	Poland	Pińczów	2014	W00311b	KY617928						
Synuchus vivalis	Poland	Chełm	2015	W00226a	MH020413						
Tachyta nana	Austria	Porrau	2015	W00201a	MH020414						
Tachyta nana	Poland	Żmiąca	2016	W00201b	MH020416						
Tachyta nana	Poland	Żmiąca	2016	W00201c	MH020415						
Tanymecus palliatus	Ukraine	Bilche Zolote	2010	W00011a	MH115472	MG987901	+	+	+	+	MG987966
Tanymecus palliatus	Ukraine	Chernivtsi	2010	W00011b	MH115473	MG987902	+	MG987625	+		MG988053
Tanymecus palliatus	Poland	Gródek	2014	W00011c	MH115474	MG987903	+	+	+		MG988049
Tenebrio molitor	Poland	Kraków	2015	W00059a	MF155962						
Tenebrio molitor	Poland	Białowieża Forest	2012	W00059b							
Tenebrio molitor	Poland	Białowieża Forest	2012	W00059c							
Tetropium fuscum	Poland	Białowieża Forest	2016	W00274a	MH115562	+		MG987642		+	
Tetropium fuscum	Poland	Białowieża Forest	2016	W00274b	MH115563						
Thanasimus formicarius	Poland	Białowieża Forest	2016	W00277a	MH020290						
Thanasimus formicarius	Poland	Odolion ad Ciechocinek	2016	W00277b	+						
Thanatophilus sinuatus	Poland	Białowieża Forest	2016	W00251a	MH115512						
Thanatophilus sinuatus	Poland	Białowieża Forest	2016	W00251b	MH115511						
Trachys minuta	Poland	Urszulin	2016	W00056a	MH115552	MG987786	MG987932	+	MG987754	MG987716	MG987974
Trachys minuta	Poland	Urszulin	2016	W00056b	+	MG987805	MG987931	+	MG987755	MG987718	MG987978
Trachys minuta	Poland	Knyszyn Forest	2016	W00056c	+	MG987787	+	+	MG987756	MG987717	MG987981
Trichodes apiarius	Poland	Alwernia	2015	W00117a	MH020326						
Trichodes apiarius	Poland	Koryciny	2015	W00117b	MH020327						
Trichodes apiarius	Poland	Koryciny	2015	W00117c							
Triplax aenea	Slovakia	Sampor	2017	W00163a	MH115490	MG987832	MG987926	MG987638	MG987748	MG987683	MG988012
Triplax russica	Poland	Kolonowskie	2015	W00162a	MH115489						
Tritoma bipustulata	Poland	Kolonowskie	2015	W00160a	MH115492						
Tritoma bipustulata	Poland	Kolonowskie	2015	W00160b	MH115493						

Tropinota hirta	Poland	Tyniec	2016	W00105a	+	
Tropinota hirta	Bulgaria	Anevo	2015	W00105b	MH020443	
Tropinota hirta	Bulgaria	Anevo	2015	W00105c		
Trypocopris vernalis	Poland	Białowieża Forest	2015	W00069a	+	
Trypocopris vernalis	Bulgaria	Karnare	2015	W00069b		
Trypocopris vernalis	Austria	Edelstal	2015	W00069c		
Trypodendron lineatum	Poland	Łapsze Niżne	2014	W00112a	MH115574	
Trypodendron lineatum	Poland	Łapsze Niżne	2014	W00112b	+	
Trypodendron lineatum	Poland	Łapsze Niżne	2014	W00112c		
Tytthaspis sedecimpunctata	Slovakia	Sampor	2017	W00176a	MH020507	
Uleiota planata	Poland	Szyk	2014	W00133a	MH115565	
Uleiota planata	Poland	Szyk	2014	W00133b	+	
Uleiota planata	Poland	Ciechocinek	2016	W00133c	+	
Uloma culinaris	Poland	Rogalin	2014	W00154a	MH020548	MG987810
Uloma culinaris	Poland	Ispina	2014	W00154b		MG987811
Uloma culinaris	Poland	Ispina	2014	W00154c	MH020549	MG987812
Valgus hemipterus	Poland	Alwernia	2015	W00076a	+	
Valgus hemipterus	Bulgaria	Brezovo Mt.	2015	W00076b	MH020440	
Valgus hemipterus	Bulgaria	Gintsi	2015	W00076c	MH020441	

Supplementary Table S5. Taxonomy and trait characteristics and *Wolbachia* infection status of examined beetle species.

Phyto – phytophagous, Myceto – mycetophagous, Pred – predators, Sapro – saprophagous, Wood – Cambioxylophagous, Forest – forest-dwellers, Open – open land-dwellers, Ubiq – ubiquitous, Copro – coprophilous, Epig – epigeic, Herb – herbophilous, Hydro – hydrophilous, Fungi – mycetophilous, Necro – necrophilous, Xylo – cambioxylophilous, Cont – continental , Medit – mediterranean, Mount – mountainous, Temp – temperate, Fragm – fragmented, Wide –widespread, Neut – neutral temperature, Warm – warm temperature, Parth – parthenogenetic, Sex – (bi)sexual, Large – large size, Medium – medium size, Small – small size, Mobile – mobile, Seden – sedentary.

No.	species	family	superfamily	infraorder	suborder	trophism	habitat preferences	micro-habitats	distribution	range	thermal preferences	reproduction mode	body size	mobility	infection	supergroup A	supergroup B
1	*Abax parallelepipedus*	Carabidae	Caraboidea	Carabiformia	Adephaga	Pred	Forest	Epig	Temp	Wide	Neut	Sex	Large	Mobil	0	0	0
2	*Abax parallelus*	Carabidae	Caraboidea	Carabiformia	Adephaga	Pred	Forest	Epig	Temp	Wide	Neut	Sex	Med	Mobil	1	1	0
3	*Adalia bipunctata*	Coccinellidae	Cucujoidea	Cucujiformia	Polyphaga	Pred	Ubiq	Herb	Temp	Wide	Neut	Sex	Med	Mobil	0	0	0
4	*Agapanthia villosoviridescens*	Cerambycidae	Chrysomeloidea	Cucujiformia	Polyphaga	Phyto	Ubiq	Herb	Temp	Wide	Neut	Sex	Small	Mobil	0	0	0
5	*Agonum marginatum*	Carabidae	Caraboidea	Carabiformia	Adephaga	Pred	Open	Hydro	Temp	Wide	Neut	Sex	Med	Mobil	1	1	1
6	*Agrilus derasofasciatus*	Buprestidae	Buprestoidea	Elateriformia	Polyphaga	Wood	Forest	Xylo	Medit	Wide	Warm	Sex	Small	Mobil	0	0	0
7	*Agrilus suvorovi*	Buprestidae	Buprestoidea	Elateriformia	Polyphaga	Wood	Forest	Xylo	Temp	Wide	Neut	Sex	Med	Mobil	0	0	0
8	*Agrypnus murinus*	Elateridae	Elateroidea	Elateriformia	Polyphaga	Pred	Open	Epig	Temp	Wide	Neut	Sex	Med	Mobil	0	0	0
9	*Alosterna tabacicolor*	Cerambycidae	Chrysomeloidea	Cucujiformia	Polyphaga	Wood	Forest	Xylo	Temp	Wide	Neut	Sex	Med	Mobil	0	0	0
10	*Altica oleracea*	Chrysomelidae	Chrysomeloidea	Cucujiformia	Polyphaga	Phyto	Ubiq	Herb	Temp	Wide	Neut	Sex	Small	Mobil	0	0	0
11	*Amara similata*	Carabidae	Caraboidea	Carabiformia	Adephaga	Pred	Open	Epig	Temp	Wide	Warm	Sex	Med	Mobil	1	1	0
12	*Amphimallon solstitialis*	Scarabaeidae	Scarabaeoidea	Scarabaeiformia	Polyphaga	Phyto	Ubiq	Herb	Temp	Wide	Neut	Sex	Med	Mobil	0	0	0
13	*Anaspis brunnipes*	Scraptiidae	Tenebrionoidea	Cucujiformia	Polyphaga	Wood	Open	Xylo	Temp	Wide	Warm	Sex	Small	Mobil	1	1	0
14	*Anaspis frontalis*	Scraptiidae	Tenebrionoidea	Cucujiformia	Polyphaga	Wood	Forest	Xylo	Temp	Wide	Neut	Sex	Small	Mobil	1	1	0
15	*Anastrangalia reyi*	Cerambycidae	Chrysomeloidea	Cucujiformia	Polyphaga	Wood	Forest	Xylo	Temp	Wide	Neut	Sex	Med	Mobil	1	1	0
16	*Anastrangalia sanguinolenta*	Cerambycidae	Chrysomeloidea	Cucujiformia	Polyphaga	Wood	Forest	Xylo	Temp	Wide	Neut	Sex	Med	Mobil	0	0	0
17	*Anatis ocellata*	Coccinellidae	Cucujoidea	Cucujiformia	Polyphaga	Pred	Forest	Herb	Temp	Wide	Neut	Sex	Med	Mobil	0	0	0
18	*Anomala dubia*	Scarabaeidae	Scarabaeoidea	Scarabaeiformia	Polyphaga	Phyto	Ubiq	Herb	Temp	Wide	Neut	Sex	Med	Mobil	0	0	0
19	*Anoplodera sexguttata*	Cerambycidae	Chrysomeloidea	Cucujiformia	Polyphaga	Wood	Forest	Xylo	Temp	Fragm	Neut	Sex	Med	Mobil	0	0	0
20	*Anoplotrupes stercorosus*	Geotrupidae	Scarabaeoidea	Scarabaeiformia	Polyphaga	Sapro	Forest	Epig	Temp	Wide	Neut	Sex	Med	Mobil	0	0	0
21	*Anostirus castaneus*	Elateridae	Elateroidea	Elateriformia	Polyphaga	Pred	Forest	Xylo	Temp	Wide	Neut	Sex	Med	Mobil	0	0	0

22	*Anthaxia nitidula*	Buprestidae	Buprestoidea	Elateriformia	Polyphaga	Wood	Forest	Xylo	Medit	Fragm	Warm	Sex	Med	Mobil	0	0	0
23	*Anthaxia quadripunctata*	Buprestidae	Buprestoidea	Elateriformia	Polyphaga	Wood	Forest	Xylo	Temp	Wide	Neut	Sex	Med	Mobil	0	0	0
24	*Anthonomus rubi*	Curculionidae	Curculionoidea	Cucujiformia	Polyphaga	Phyto	Ubiq	Herb	Temp	Wide	Neut	Sex	Small	Mobil	1	1	1
25	*Aphodius ater*	Scarabaeidae	Scarabaeoidea	Scarabaeiformia	Polyphaga	Sapro	Open	Copro	Temp	Wide	Neut	Sex	Med	Mobil	0	0	1
26	*Aphodius depressus*	Scarabaeidae	Scarabaeoidea	Scarabaeiformia	Polyphaga	Sapro	Open	Copro	Temp	Wide	Neut	Sex	Med	Mobil	0	0	0
27	*Aphodius erraticus*	Scarabaeidae	Scarabaeoidea	Scarabaeiformia	Polyphaga	Sapro	Open	Copro	Temp	Wide	Neut	Sex	Med	Mobil	0	0	0
28	*Aphodius fossor*	Scarabaeidae	Scarabaeoidea	Scarabaeiformia	Polyphaga	Sapro	Ubiq	Copro	Temp	Wide	Neut	Sex	Med	Mobil	0	0	0
29	*Aphodius granarius*	Scarabaeidae	Scarabaeoidea	Scarabaeiformia	Polyphaga	Sapro	Open	Copro	Temp	Wide	Neut	Sex	Med	Mobil	1	0	1
30	*Aphodius haemorrhoidalis*	Scarabaeidae	Scarabaeoidea	Scarabaeiformia	Polyphaga	Sapro	Open	Copro	Temp	Wide	Neut	Sex	Med	Mobil	1	0	1
31	*Aphodius luridus*	Scarabaeidae	Scarabaeoidea	Scarabaeiformia	Polyphaga	Sapro	Open	Copro	Temp	Wide	Warm	Sex	Med	Mobil	0	0	0
32	*Aphodius pedellus*	Scarabaeidae	Scarabaeoidea	Scarabaeiformia	Polyphaga	Sapro	Open	Copro	Temp	Wide	Neut	Sex	Med	Mobil	0	0	0
33	*Aphodius pusillus*	Scarabaeidae	Scarabaeoidea	Scarabaeiformia	Polyphaga	Sapro	Open	Copro	Temp	Wide	Neut	Sex	Med	Mobil	0	0	0
34	*Aphodius scrutator*	Scarabaeidae	Scarabaeoidea	Scarabaeiformia	Polyphaga	Sapro	Open	Copro	Cont	Fragm	Warm	Sex	Med	Mobil	0	0	0
35	*Aphodius sphacelatus*	Scarabaeidae	Scarabaeoidea	Scarabaeiformia	Polyphaga	Sapro	Open	Copro	Temp	Wide	Neut	Sex	Med	Mobil	0	0	0
36	*Aphodius sticticus*	Scarabaeidae	Scarabaeoidea	Scarabaeiformia	Polyphaga	Sapro	Forest	Copro	Temp	Wide	Neut	Sex	Med	Mobil	1	0	1
37	*Aphthona venustula*	Chrysomelidae	Chrysomeloidea	Cucujiformia	Polyphaga	Phyto	Open	Herb	Medit	Fragm	Warm	Sex	Small	Mobil	1	0	1
38	*Arhopalus rusticus*	Cerambycidae	Chrysomeloidea	Cucujiformia	Polyphaga	Wood	Forest	Xylo	Temp	Wide	Neut	Sex	Large	Mobil	1	1	0
39	*Aromia moschata*	Cerambycidae	Chrysomeloidea	Cucujiformia	Polyphaga	Wood	Forest	Xylo	Temp	Wide	Neut	Sex	Med	Mobil	0	0	0
40	*Athous subfuscus*	Elateridae	Elateroidea	Elateriformia	Polyphaga	Pred	Ubiq	Epig	Temp	Wide	Neut	Sex	Med	Mobil	0	0	0
41	*Attagenus pellio*	Dermestidae	Bostrichoidea	Bostrichiformia	Polyphaga	Sapro	Ubiq	Necro	Temp	Wide	Neut	Sex	Med	Mobil	0	0	0
42	*Batophila rubi*	Chrysomelidae	Chrysomeloidea	Cucujiformia	Polyphaga	Phyto	Ubiq	Herb	Temp	Wide	Neut	Sex	Small	Mobil	1	1	0
43	*Bembidion articulatum*	Carabidae	Caraboidea	Carabiformia	Adephaga	Pred	Open	Hydro	Temp	Wide	Neut	Sex	Med	Mobil	0	0	0
44	*Bembidion decorum*	Carabidae	Caraboidea	Carabiformia	Adephaga	Pred	Open	Hydro	Mount	Fragm	Neut	Sex	Med	Mobil	0	0	0
45	*Bembidion modestum*	Carabidae	Caraboidea	Carabiformia	Adephaga	Pred	Open	Hydro	Mount	Fragm	Neut	Sex	Med	Mobil	0	0	0
46	*Bembidion punctulatum*	Carabidae	Caraboidea	Carabiformia	Adephaga	Pred	Open	Hydro	Temp	Wide	Neut	Sex	Med	Mobil	1	0	1
47	*Bembidion varicolor*	Carabidae	Caraboidea	Carabiformia	Adephaga	Pred	Open	Hydro	Mount	Fragm	Neut	Sex	Med	Mobil	1	1	1
48	*Bembidion varium*	Carabidae	Caraboidea	Carabiformia	Adephaga	Pred	Open	Hydro	Temp	Wide	Neut	Sex	Med	Mobil	1	0	1
49	*Bitoma crenata*	Zopheridae	Tenebrionoidea	Cucujiformia	Polyphaga	Sapro	Forest	Xylo	Temp	Wide	Neut	Sex	Small	Seden	0	0	0
50	*Blitopertha lineolata*	Scarabaeidae	Scarabaeoidea	Scarabaeiformia	Polyphaga	Phyto	Open	Herb	Cont	Fragm	Warm	Sex	Med	Mobil	0	0	0
51	*Bostrichus capucinus*	Bostrichidae	Bostrichoidea	Bostrichiformia	Polyphaga	Wood	Forest	Xylo	Temp	Wide	Neut	Sex	Med	Mobil	0	0	0
52	*Brachyleptura maculicornis*	Cerambycidae	Chrysomeloidea	Cucujiformia	Polyphaga	Wood	Forest	Xylo	Temp	Wide	Neut	Sex	Med	Mobil	0	0	0
53	*Broscus cephalotes*	Carabidae	Caraboidea	Carabiformia	Adephaga	Pred	Open	Epig	Temp	Wide	Neut	Sex	Med	Mobil	0	0	0
54	*Bruchidius cf. varius*	Chrysomelidae	Chrysomeloidea	Cucujiformia	Polyphaga	Phyto	Open	Herb	Medit	Wide	Warm	Sex	Small	Mobil	0	0	0
55	*Bruchus affinis*	Chrysomelidae	Chrysomeloidea	Cucujiformia	Polyphaga	Phyto	Open	Herb	Cont	Wide	Warm	Sex	Small	Mobil	0	0	0
56	*Bruchus atomarius*	Chrysomelidae	Chrysomeloidea	Cucujiformia	Polyphaga	Phyto	Open	Herb	Temp	Wide	Neut	Sex	Small	Mobil	0	0	0

57	*Bruchus brachialis*	Chrysomelidae	Chrysomeloidea	Cucujiformia	Polyphaga	Phyto	Open	Herb	Medit	Fragm	Warm	Sex	Small	Mobil	0	0	0
58	*Bruchus loti*	Chrysomelidae	Chrysomeloidea	Cucujiformia	Polyphaga	Phyto	Open	Herb	Cont	Wide	Neut	Sex	Small	Mobil	0	0	0
59	*Bruchus viciae*	Chrysomelidae	Chrysomeloidea	Cucujiformia	Polyphaga	Phyto	Open	Herb	Medit	Wide	Warm	Sex	Small	Mobil	0	0	0
60	*Caccobius schreberi*	Scarabaeidae	Scarabaeoidea	Scarabaeiformia	Polyphaga	Sapro	Open	Copro	Temp	Fragm	Warm	Sex	Med	Mobil	0	0	0
61	*Calathus fuscipes*	Carabidae	Caraboidea	Carabiformia	Adephaga	Pred	Ubiq	Epig	Temp	Wide	Neut	Sex	Med	Mobil	0	0	0
62	*Calvia quatuordecimpunctata*	Coccinellidae	Cucujoidea	Cucujiformia	Polyphaga	Pred	Forest	Herb	Temp	Wide	Neut	Sex	Med	Mobil	0	0	0
63	*Cantharis livida*	Cantharidae	Elateroidea	Elateriformia	Polyphaga	Pred	Ubiq	Herb	Temp	Wide	Neut	Sex	Med	Mobil	0	0	0
64	*Cantharis nigricans*	Cantharidae	Elateroidea	Elateriformia	Polyphaga	Pred	Forest	Herb	Temp	Wide	Neut	Sex	Med	Mobil	0	0	0
65	*Cantharis rustica*	Cantharidae	Elateroidea	Elateriformia	Polyphaga	Pred	Ubiq	Herb	Temp	Wide	Neut	Sex	Med	Mobil	0	0	0
66	*Carabus cancellatus*	Carabidae	Caraboidea	Carabiformia	Adephaga	Pred	Open	Epig	Temp	Wide	Neut	Sex	Large	Seden	0	0	0
67	*Carabus coriaceus*	Carabidae	Caraboidea	Carabiformia	Adephaga	Pred	Forest	Epig	Temp	Wide	Neut	Sex	Large	Seden	0	0	0
68	*Carabus glabratus*	Carabidae	Caraboidea	Carabiformia	Adephaga	Pred	Forest	Epig	Temp	Wide	Neut	Sex	Large	Seden	1	0	1
69	*Carabus granulatus*	Carabidae	Caraboidea	Carabiformia	Adephaga	Pred	Forest	Epig	Temp	Wide	Neut	Sex	Large	Seden	0	0	0
70	*Carabus hortensis*	Carabidae	Caraboidea	Carabiformia	Adephaga	Pred	Forest	Epig	Temp	Wide	Neut	Sex	Large	Seden	0	0	0
71	*Carabus nemoralis*	Carabidae	Caraboidea	Carabiformia	Adephaga	Pred	Forest	Epig	Temp	Wide	Neut	Sex	Large	Seden	0	0	0
72	*Carabus violaceus*	Carabidae	Caraboidea	Carabiformia	Adephaga	Pred	Forest	Epig	Temp	Wide	Neut	Sex	Large	Seden	0	0	0
73	*Cassida viridis*	Chrysomelidae	Chrysomeloidea	Cucujiformia	Polyphaga	Phyto	Open	Herb	Temp	Wide	Neut	Sex	Small	Mobil	1	1	0
74	*Catapion jaffense*	Apionidae	Curculionoidea	Cucujiformia	Polyphaga	Phyto	Open	Herb	Temp	Wide	Neut	Sex	Small	Mobil	1	1	1
75	*Catapion koestlini*	Apionidae	Curculionoidea	Cucujiformia	Polyphaga	Phyto	Open	Herb	Temp	Fragm	Neut	Sex	Small	Mobil	0	0	0
76	*Catapion meieri*	Apionidae	Curculionoidea	Cucujiformia	Polyphaga	Phyto	Open	Herb	Temp	Wide	Neut	Sex	Small	Mobil	1	0	1
77	*Catapion pubescens*	Apionidae	Curculionoidea	Cucujiformia	Polyphaga	Phyto	Open	Herb	Temp	Wide	Neut	Sex	Small	Mobil	0	0	0
78	*Catapion seniculus*	Apionidae	Curculionoidea	Cucujiformia	Polyphaga	Phyto	Open	Herb	Temp	Wide	Neut	Sex	Small	Mobil	1	0	1
79	*Centricnemus leucogrammus*	Curculionidae	Curculionoidea	Cucujiformia	Polyphaga	Phyto	Open	Herb	Cont	Fragm	Warm	Sex	Small	Seden	0	0	0
80	*Cetonia aurata*	Scarabaeidae	Scarabaeoidea	Scarabaeiformia	Polyphaga	Phyto	Ubiq	Xylo	Temp	Wide	Warm	Sex	Med	Mobil	0	0	0
81	*Ceutorhynchus obstrictus*	Curculionidae	Curculionoidea	Cucujiformia	Polyphaga	Phyto	Open	Herb	Temp	Wide	Neut	Sex	Small	Mobil	1	1	0
82	*Ceutorhynchus typhae*	Curculionidae	Curculionoidea	Cucujiformia	Polyphaga	Phyto	Ubiq	Herb	Temp	Wide	Neut	Sex	Small	Mobil	1	1	0
83	*Cheilotoma musciformis*	Chrysomelidae	Chrysomeloidea	Cucujiformia	Polyphaga	Phyto	Open	Herb	Cont	Fragm	Warm	Sex	Small	Mobil	0	0	0
84	*Chilocorus renipustulatus*	Coccinellidae	Cucujoidea	Cucujiformia	Polyphaga	Pred	Forest	Herb	Temp	Wide	Neut	Sex	Med	Mobil	0	0	0
85	*Chlaenius nitidulus*	Carabidae	Caraboidea	Carabiformia	Adephaga	Pred	Open	Hydro	Temp	Wide	Neut	Sex	Med	Mobil	0	0	0
86	*Chlaenius tibialis*	Carabidae	Caraboidea	Carabiformia	Adephaga	Pred	Open	Hydro	Temp	Wide	Neut	Sex	Med	Mobil	0	0	0
87	*Chrysanthia geniculata*	Oedemeridae	Tenebrionoidea	Cucujiformia	Polyphaga	Wood	Forest	Xylo	Temp	Fragm	Neut	Sex	Med	Mobil	0	0	0
88	*Chrysolina herbacea*	Chrysomelidae	Chrysomeloidea	Cucujiformia	Polyphaga	Phyto	Open	Herb	Medit	Wide	Neut	Sex	Med	Mobil	1	1	0
89	*Chrysolina polita*	Chrysomelidae	Chrysomeloidea	Cucujiformia	Polyphaga	Phyto	Open	Herb	Temp	Wide	Neut	Sex	Med	Mobil	0	0	0
90	*Chrysolina varians*	Chrysomelidae	Chrysomeloidea	Cucujiformia	Polyphaga	Phyto	Open	Herb	Temp	Wide	Neut	Sex	Med	Mobil	0	0	0
91	*Cicindela hybrida*	Carabidae	Caraboidea	Carabiformia	Adephaga	Pred	Open	Epig	Temp	Wide	Warm	Sex	Med	Mobil	0	0	0

92	*Clerus mutillarius*	Cleridae	Cleroidea	Cucujiformia	Polyphaga	Pred	Ubiq	Xylo	Medit	Wide	Warm	Sex	Med	Mobil	0	0	0
93	*Clivina collaris*	Carabidae	Caraboidea	Carabiformia	Adephaga	Pred	Open	Hydro	Temp	Wide	Neut	Sex	Med	Mobil	0	0	0
94	*Coccinella quinquepunctata*	Coccinellidae	Cucujoidea	Cucujiformia	Polyphaga	Pred	Ubiq	Herb	Temp	Wide	Neut	Sex	Med	Mobil	0	0	0
95	*Coccinella septempunctata*	Coccinellidae	Cucujoidea	Cucujiformia	Polyphaga	Pred	Ubiq	Herb	Temp	Wide	Neut	Sex	Med	Mobil	0	0	0
96	*Coccinula quatuordecimpustulata*	Coccinellidae	Cucujoidea	Cucujiformia	Polyphaga	Pred	Open	Herb	Temp	Wide	Warm	Sex	Small	Mobil	1	1	0
97	*Combocerus glaber*	Erotylidae	Cucujoidea	Cucujiformia	Polyphaga	Myceto	Forest	Fungi	Temp	Wide	Neut	Sex	Small	Mobil	1	0	1
98	*Coraebus elatus*	Buprestidae	Buprestoidea	Elateriformia	Polyphaga	Phyto	Open	Herb	Cont	Fragm	Warm	Sex	Small	Mobil	0	0	0
99	*Crepidodera aurata*	Chrysomelidae	Chrysomeloidea	Cucujiformia	Polyphaga	Phyto	Forest	Herb	Temp	Wide	Neut	Sex	Small	Mobil	1	1	0
100	*Crioceris asparagi*	Chrysomelidae	Chrysomeloidea	Cucujiformia	Polyphaga	Phyto	Open	Herb	Temp	Wide	Neut	Sex	Med	Mobil	0	0	0
101	*Crioceris quatuordecimpunctata*	Chrysomelidae	Chrysomeloidea	Cucujiformia	Polyphaga	Phyto	Open	Herb	Cont	Fragm	Warm	Sex	Med	Mobil	1	1	1
102	*Crioceris quinquepunctata*	Chrysomelidae	Chrysomeloidea	Cucujiformia	Polyphaga	Phyto	Open	Herb	Cont	Fragm	Warm	Sex	Med	Mobil	1	1	1
103	*Cryptocephalus bameuli*	Chrysomelidae	Chrysomeloidea	Cucujiformia	Polyphaga	Phyto	Open	Herb	Cont	Wide	Neut	Sex	Small	Mobil	0	0	0
104	*Cryptocephalus bipunctatus*	Chrysomelidae	Chrysomeloidea	Cucujiformia	Polyphaga	Phyto	Ubiq	Herb	Temp	Wide	Neut	Sex	Med	Mobil	0	0	0
105	*Cryptocephalus flavipes*	Chrysomelidae	Chrysomeloidea	Cucujiformia	Polyphaga	Phyto	Ubiq	Herb	Cont	Wide	Warm	Sex	Small	Mobil	0	0	0
106	*Cryptocephalus hypochoeridis*	Chrysomelidae	Chrysomeloidea	Cucujiformia	Polyphaga	Phyto	Open	Herb	Temp	Wide	Neut	Sex	Med	Mobil	1	1	0
107	*Cryptocephalus moraei*	Chrysomelidae	Chrysomeloidea	Cucujiformia	Polyphaga	Phyto	Open	Herb	Temp	Wide	Neut	Sex	Small	Mobil	0	0	0
108	*Cryptocephalus quadripustulatus*	Chrysomelidae	Chrysomeloidea	Cucujiformia	Polyphaga	Phyto	Forest	Herb	Temp	Wide	Neut	Sex	Small	Mobil	0	0	0
109	*Cryptocephalus sericeus*	Chrysomelidae	Chrysomeloidea	Cucujiformia	Polyphaga	Phyto	Open	Herb	Cont	Wide	Neut	Sex	Med	Mobil	0	0	0
110	*Cryptocephalus signatifrons*	Chrysomelidae	Chrysomeloidea	Cucujiformia	Polyphaga	Phyto	Ubiq	Herb	Medit	Wide	Warm	Sex	Small	Mobil	0	0	0
111	*Cryptocephalus violaceus*	Chrysomelidae	Chrysomeloidea	Cucujiformia	Polyphaga	Phyto	Ubiq	Herb	Cont	Wide	Warm	Sex	Med	Mobil	0	0	0
112	*Cyanapion afer*	Apionidae	Curculionoidea	Cucujiformia	Polyphaga	Phyto	Open	Herb	Temp	Fragm	Neut	Sex	Small	Mobil	1	1	1
113	*Cyanapion gnarum*	Apionidae	Curculionoidea	Cucujiformia	Polyphaga	Phyto	Open	Herb	Temp	Fragm	Neut	Sex	Small	Mobil	0	0	0
114	*Cyanapion gyllenhalii*	Apionidae	Curculionoidea	Cucujiformia	Polyphaga	Phyto	Open	Herb	Temp	Wide	Neut	Sex	Small	Mobil	1	1	1
115	*Cyanapion platalea*	Apionidae	Curculionoidea	Cucujiformia	Polyphaga	Phyto	Open	Herb	Temp	Wide	Neut	Sex	Small	Mobil	0	0	0
116	*Cyanapion columbinum*	Apionidae	Curculionoidea	Cucujiformia	Polyphaga	Phyto	Open	Herb	Temp	Wide	Neut	Sex	Small	Mobil	0	0	0
117	*Cyanapion spencii*	Apionidae	Curculionoidea	Cucujiformia	Polyphaga	Phyto	Open	Herb	Temp	Wide	Neut	Sex	Small	Mobil	1	1	1
118	*Dacne bipustulata*	Erotylidae	Cucujoidea	Cucujiformia	Polyphaga	Myceto	Forest	Fungi	Temp	Wide	Neut	Sex	Small	Mobil	0	0	0
119	*Dacne rufifrons*	Erotylidae	Cucujoidea	Cucujiformia	Polyphaga	Myceto	Forest	Fungi	Temp	Wide	Neut	Sex	Small	Mobil	1	1	0
120	*Dalopius marginatus*	Elateridae	Elateroidea	Elateriformia	Polyphaga	Pred	Ubiq	Epig	Temp	Wide	Neut	Sex	Med	Mobil	0	0	0
121	*Dascillus cervinus*	Dascillidae	Dascilloidea	Elateriformia	Polyphaga	Phyto	Open	Herb	Mount	Wide	Neut	Sex	Med	Mobil	0	0	0
122	*Dasytes plumbeus*	Dasytidae	Cleroidea	Cucujiformia	Polyphaga	Pred	Ubiq	Xylo	Temp	Wide	Neut	Sex	Med	Mobil	0	0	0
123	*Deporaus betulae*	Rhynchitidae	Curculionoidea	Cucujiformia	Polyphaga	Phyto	Ubiq	Herb	Temp	Wide	Neut	Sex	Small	Mobil	0	0	0
124	*Dermestes lardarius*	Dermestidae	Bostrichoidea	Bostrichiformia	Polyphaga	Sapro	Ubiq	Necro	Temp	Wide	Neut	Sex	Med	Mobil	0	0	0
125	*Derocrepis rufipes*	Chrysomelidae	Chrysomeloidea	Cucujiformia	Polyphaga	Phyto	Open	Herb	Temp	Wide	Neut	Sex	Small	Mobil	0	0	0
126	*Dieperis boleti*	Tenebrionidae	Tenebrionoidea	Cucujiformia	Polyphaga	Myceto	Forest	Fungi	Temp	Wide	Neut	Sex	Med	Mobil	0	0	0

127	*Dinoptera collaris*	Cerambycidae	Chrysomeloidea	Cucujiformia	Polyphaga	Wood	Forest	Xylo	Temp	Wide	Neut	Sex	Med	Mobil	0	0	0
128	*Dolichosoma lineare*	Dasytidae	Cleroidea	Cucujiformia	Polyphaga	Pred	Open	Herb	Cont	Wide	Warm	Sex	Med	Mobil	0	0	0
129	*Dorcus parallelipipedus*	Lucanidae	Scarabaeoidea	Scarabaeiformia	Polyphaga	Wood	Forest	Xylo	Medit	Wide	Warm	Sex	Large	Mobil	0	0	0
130	*Dromius agilis*	Carabidae	Caraboidea	Carabiformia	Adephaga	Pred	Forest	Xylo	Temp	Wide	Neut	Sex	Med	Mobil	1	1	0
131	*Dryocoetes autographus*	Curculionidae	Curculionoidea	Cucujiformia	Polyphaga	Wood	Forest	Xylo	Temp	Wide	Neut	Sex	Small	Mobil	0	0	0
132	*Drypta dentata*	Carabidae	Caraboidea	Carabiformia	Adephaga	Pred	Open	Epig	Medit	Fragm	Warm	Sex	Med	Mobil	0	0	0
133	*Dyschirius digitatus*	Carabidae	Caraboidea	Carabiformia	Adephaga	Pred	Open	Hydro	Temp	Fragm	Neut	Sex	Small	Mobil	0	0	0
134	*Elaphropus quadrisignatus*	Carabidae	Caraboidea	Carabiformia	Adephaga	Pred	Open	Hydro	Medit	Wide	Warm	Sex	Small	Mobil	0	0	0
135	*Elaphrus aureus*	Carabidae	Caraboidea	Carabiformia	Adephaga	Pred	Open	Hydro	Temp	Wide	Neut	Sex	Med	Mobil	0	0	0
136	*Elaphrus riparius*	Carabidae	Caraboidea	Carabiformia	Adephaga	Pred	Open	Hydro	Temp	Wide	Neut	Sex	Med	Mobil	0	0	0
137	*Etorofus pubescens*	Cerambycidae	Chrysomeloidea	Cucujiformia	Polyphaga	Wood	Forest	Xylo	Temp	Wide	Neut	Sex	Med	Mobil	1	1	0
138	*Euoniticellus fulvus*	Scarabaeidae	Scarabaeoidea	Scarabaeiformia	Polyphaga	Sapro	Open	Copro	Cont	Fragm	Warm	Sex	Med	Mobil	0	0	0
139	*Eusomus ovulum*	Curculionidae	Curculionoidea	Cucujiformia	Polyphaga	Phyto	Open	Herb	Cont	Wide	Warm	Parth	Med	Seden	1	1	0
140	*Eutrichapion melancholicum*	Apionidae	Curculionoidea	Cucujiformia	Polyphaga	Phyto	Open	Herb	Temp	Wide	Neut	Sex	Small	Mobil	0	0	0
141	*Eutrichapion viciae*	Apionidae	Curculionoidea	Cucujiformia	Polyphaga	Phyto	Open	Herb	Temp	Wide	Neut	Sex	Small	Mobil	0	0	0
142	*Exochomus quadripustulatus*	Coccinellidae	Cucujoidea	Cucujiformia	Polyphaga	Pred	Forest	Herb	Temp	Wide	Neut	Sex	Med	Mobil	0	0	0
143	*Galeruca tanaceti*	Chrysomelidae	Chrysomeloidea	Cucujiformia	Polyphaga	Phyto	Open	Herb	Temp	Wide	Neut	Sex	Med	Mobil	0	0	0
144	*Galerucella tenella*	Chrysomelidae	Chrysomeloidea	Cucujiformia	Polyphaga	Phyto	Open	Herb	Temp	Wide	Neut	Sex	Small	Mobil	0	0	0
145	*Gnaptor spinimanus*	Tenebrionidae	Tenebrionoidea	Cucujiformia	Polyphaga	Phyto	Open	Epig	Cont	Fragm	Warm	Sex	Large	Seden	0	0	0
146	*Harpalus affinis*	Carabidae	Caraboidea	Carabiformia	Adephaga	Pred	Open	Epig	Temp	Wide	Neut	Sex	Med	Mobil	0	0	0
147	*Harpalus rufipes*	Carabidae	Caraboidea	Carabiformia	Adephaga	Pred	Open	Epig	Temp	Wide	Neut	Sex	Med	Mobil	0	0	0
148	*Hippodamia variegata*	Coccinellidae	Cucujoidea	Cucujiformia	Polyphaga	Pred	Open	Herb	Temp	Wide	Neut	Sex	Med	Mobil	0	0	0
149	*Hylobius abietis*	Curculionidae	Curculionoidea	Cucujiformia	Polyphaga	Wood	Forest	Herb	Temp	Wide	Neut	Sex	Med	Mobil	1	1	0
150	*Hylotrupes bajulus*	Cerambycidae	Chrysomeloidea	Cucujiformia	Polyphaga	Wood	Ubiq	Xylo	Temp	Wide	Neut	Sex	Med	Mobil	0	0	0
151	*Ips typographus*	Curculionidae	Curculionoidea	Cucujiformia	Polyphaga	Wood	Forest	Xylo	Temp	Wide	Neut	Sex	Small	Mobil	0	0	0
152	*Kateretes pedicularius*	Nitidulidae	Cucujoidea	Cucujiformia	Polyphaga	Phyto	Open	Herb	Temp	Wide	Neut	Sex	Small	Mobil	0	0	0
153	*Lagria hirta*	Tenebrionidae	Tenebrionoidea	Cucujiformia	Polyphaga	Phyto	Ubiq	Herb	Temp	Wide	Neut	Sex	Med	Mobil	1	1	0
154	*Litargus connexus*	Mycetophagidae	Tenebrionoidea	Cucujiformia	Polyphaga	Myceto	Forest	Fungi	Temp	Wide	Neut	Sex	Small	Mobil	0	0	0
155	*Lixus filiformis*	Curculionidae	Curculionoidea	Cucujiformia	Polyphaga	Phyto	Open	Herb	Temp	Fragm	Warm	Sex	Med	Seden	0	0	0
156	*Lochmaea caprea*	Chrysomelidae	Chrysomeloidea	Cucujiformia	Polyphaga	Phyto	Ubiq	Herb	Temp	Wide	Neut	Sex	Med	Mobil	0	0	0
157	*Longitarsus exsoletus*	Chrysomelidae	Chrysomeloidea	Cucujiformia	Polyphaga	Phyto	Ubiq	Herb	Temp	Wide	Neut	Sex	Small	Mobil	0	0	0
158	*Longitarsus nasturtii*	Chrysomelidae	Chrysomeloidea	Cucujiformia	Polyphaga	Phyto	Ubiq	Herb	Temp	Wide	Neut	Sex	Small	Mobil	0	0	0
159	*Loricera pilicornis*	Carabidae	Caraboidea	Carabiformia	Adephaga	Pred	Open	Hydro	Temp	Wide	Neut	Sex	Med	Mobil	0	0	0
160	*Luperus flavipes*	Chrysomelidae	Chrysomeloidea	Cucujiformia	Polyphaga	Phyto	Forest	Herb	Temp	Wide	Neut	Sex	Small	Mobil	1	1	0
161	*Luperus luperus*	Chrysomelidae	Chrysomeloidea	Cucujiformia	Polyphaga	Phyto	Forest	Herb	Temp	Wide	Neut	Sex	Small	Mobil	1	1	0

162	*Lygistopterus sanguineus*	Lycidae	Elateroidea	Elateriformia	Polyphaga	Sapro	Forest	Xylo	Temp	Wide	Neut	Sex	Med	Mobil	0	0	0
163	*Mecinus pascuorum*	Curculionidae	Curculionoidea	Cucujiformia	Polyphaga	Phyto	Open	Herb	Temp	Wide	Neut	Sex	Small	Mobil	1	0	1
164	*Melolontha melolontha*	Scarabaeidae	Scarabaeoidea	Scarabaeiformia	Polyphaga	Phyto	Ubiq	Herb	Temp	Wide	Neut	Sex	Large	Mobil	0	0	0
165	*Metacantharis discoidea*	Cantharidae	Elateroidea	Elateriformia	Polyphaga	Pred	Forest	Herb	Temp	Wide	Neut	Sex	Med	Mobil	0	0	0
166	*Monochamus galloprovincialis*	Cerambycidae	Chrysomeloidea	Cucujiformia	Polyphaga	Wood	Forest	Xylo	Temp	Wide	Neut	Sex	Large	Mobil	0	0	0
167	*Monochamus sartor*	Cerambycidae	Chrysomeloidea	Cucujiformia	Polyphaga	Wood	Forest	Xylo	Mount	Wide	Neut	Sex	Large	Mobil	1	1	1
168	*Monochamus sutor*	Cerambycidae	Chrysomeloidea	Cucujiformia	Polyphaga	Wood	Forest	Xylo	Mount	Wide	Neut	Sex	Large	Mobil	0	0	0
169	*Mordella brachyura*	Mordellidae	Tenebrionoidea	Cucujiformia	Polyphaga	Wood	Ubiq	Xylo	Temp	Wide	Neut	Sex	Med	Mobil	0	0	0
170	*Mordella holomelaena*	Mordellidae	Tenebrionoidea	Cucujiformia	Polyphaga	Wood	Forest	Xylo	Temp	Wide	Neut	Sex	Med	Mobil	0	0	0
171	*Musaria affinis*	Cerambycidae	Chrysomeloidea	Cucujiformia	Polyphaga	Phyto	Ubiq	Herb	Temp	Wide	Neut	Sex	Med	Mobil	0	0	0
172	*Mycetophagus ater*	Mycetophagidae	Tenebrionoidea	Cucujiformia	Polyphaga	Myceto	Forest	Fungi	Temp	Wide	Neut	Sex	Med	Mobil	0	0	0
173	*Mycetophagus multipunctatus*	Mycetophagidae	Tenebrionoidea	Cucujiformia	Polyphaga	Myceto	Forest	Fungi	Temp	Wide	Neut	Sex	Med	Mobil	0	0	0
174	*Mycetophagus piceus*	Mycetophagidae	Tenebrionoidea	Cucujiformia	Polyphaga	Myceto	Forest	Fungi	Temp	Wide	Neut	Sex	Med	Mobil	0	0	0
175	*Mycetophagus quadripustulatus*	Mycetophagidae	Tenebrionoidea	Cucujiformia	Polyphaga	Myceto	Forest	Fungi	Temp	Wide	Neut	Sex	Med	Mobil	0	0	0
176	*Myrrha octodecimguttata*	Coccinellidae	Cucujoidea	Cucujiformia	Polyphaga	Pred	Forest	Herb	Temp	Wide	Neut	Sex	Med	Mobil	1	1	0
177	*Neatus picipes*	Tenebrionidae	Tenebrionoidea	Cucujiformia	Polyphaga	Sapro	Forest	Xylo	Temp	Wide	Neut	Sex	Med	Mobil	0	0	0
178	*Nebria picicornis*	Carabidae	Caraboidea	Carabiformia	Adephaga	Pred	Open	Hydro	Mount	Fragm	Neut	Sex	Med	Mobil	1	1	0
179	*Neocoenorhinus germanicus*	Rhynchitidae	Curculionoidea	Cucujiformia	Polyphaga	Phyto	Open	Herb	Temp	Wide	Neut	Sex	Small	Mobil	1	0	1
180	*Nedyus quadrimaculatus*	Curculionidae	Curculionoidea	Cucujiformia	Polyphaga	Phyto	Ubiq	Herb	Temp	Wide	Neut	Sex	Small	Mobil	1	1	0
181	*Nicrophorus vespilloides*	Silphidae	Staphylinoidea	Staphyliniformia	Polyphaga	Sapro	Ubiq	Necro	Temp	Wide	Neut	Sex	Med	Mobil	1	1	0
182	*Nivellia sanguinosa*	Cerambycidae	Chrysomeloidea	Cucujiformia	Polyphaga	Wood	Forest	Xylo	Mount	Fragm	Neut	Sex	Med	Mobil	0	0	0
183	*Notiophilus biguttatus*	Carabidae	Caraboidea	Carabiformia	Adephaga	Pred	Forest	Epig	Temp	Wide	Neut	Sex	Small	Mobil	0	0	0
184	*Notoxus monoceros*	Anthicidae	Tenebrionoidea	Cucujiformia	Polyphaga	Sapro	Ubiq	Herb	Temp	Wide	Neut	Sex	Small	Mobil	1	1	1
185	*Obrium brunneum*	Cerambycidae	Chrysomeloidea	Cucujiformia	Polyphaga	Wood	Forest	Xylo	Temp	Wide	Neut	Sex	Small	Mobil	0	0	0
186	*Ocypus olens*	Staphylinidae	Staphylinoidea	Staphyliniformia	Polyphaga	Pred	Forest	Epig	Temp	Wide	Neut	Sex	Large	Mobil	0	0	0
187	*Oedemera femorata*	Oedemeridae	Tenebrionoidea	Cucujiformia	Polyphaga	Phyto	Open	Herb	Temp	Wide	Neut	Sex	Med	Mobil	0	0	0
188	*Oedemera flavipes*	Oedemeridae	Tenebrionoidea	Cucujiformia	Polyphaga	Phyto	Open	Herb	Medit	Wide	Warm	Sex	Med	Mobil	0	0	0
189	*Oenopia conglobata*	Coccinellidae	Cucujoidea	Cucujiformia	Polyphaga	Pred	Forest	Herb	Temp	Wide	Neut	Sex	Small	Mobil	0	0	0
190	*Oiceoptoma thoracica*	Silphidae	Staphylinoidea	Staphyliniformia	Polyphaga	Sapro	Forest	Necro	Temp	Wide	Neut	Sex	Med	Mobil	1	1	0
191	*Omalisus fontisbellaquaei*	Omalisidae	Elateroidea	Elateriformia	Polyphaga	Pred	Open	Herb	Temp	Wide	Neut	Sex	Med	Mobil	0	0	0
192	*Omonadus floralis*	Anthicidae	Tenebrionoidea	Cucujiformia	Polyphaga	Sapro	Open	Herb	Temp	Wide	Neut	Sex	Small	Mobil	0	0	0
193	*Omophron limbatum*	Carabidae	Caraboidea	Carabiformia	Adephaga	Pred	Open	Hydro	Temp	Wide	Neut	Sex	Med	Mobil	0	0	0
194	*Onthophagus fracticornis*	Scarabaeidae	Scarabaeoidea	Scarabaeiformia	Polyphaga	Sapro	Open	Copro	Temp	Wide	Neut	Sex	Med	Mobil	1	1	0
195	*Onthophagus ovatus*	Scarabaeidae	Scarabaeoidea	Scarabaeiformia	Polyphaga	Sapro	Open	Copro	Temp	Wide	Warm	Sex	Med	Mobil	0	0	0
196	*Onthophagus ruficapillus*	Scarabaeidae	Scarabaeoidea	Scarabaeiformia	Polyphaga	Sapro	Open	Copro	Temp	Wide	Neut	Sex	Med	Mobil	1	1	0

197	*Onthophagus similis*	Scarabaeidae	Scarabaeoidea	Scarabaeiformia	Polyphaga	Sapro	Open	Copro	Cont	Fragm	Warm	Sex	Med	Mobil	1	1	0
198	*Onthophagus taurus*	Scarabaeidae	Scarabaeoidea	Scarabaeiformia	Polyphaga	Sapro	Open	Copro	Cont	Fragm	Warm	Sex	Med	Mobil	1	1	0
199	*Ophonus laticollis*	Carabidae	Caraboidea	Carabiformia	Adephaga	Pred	Open	Epig	Temp	Wide	Neut	Sex	Med	Mobil	0	0	0
200	*Otiorhynchus perdix*	Curculionidae	Curculionoidea	Cucujiformia	Polyphaga	Phyto	Forest	Herb	Mount	Fragm	Neut	Parth	Med	Seden	0	0	0
201	*Otiorhynchus riessi*	Curculionidae	Curculionoidea	Cucujiformia	Polyphaga	Phyto	Forest	Herb	Mount	Fragm	Neut	Sex	Med	Seden	0	0	0
202	*Oulema gallaeciana*	Chrysomelidae	Chrysomeloidea	Cucujiformia	Polyphaga	Phyto	Open	Herb	Temp	Wide	Neut	Sex	Small	Mobil	0	0	0
203	*Oxymirus cursor*	Cerambycidae	Chrysomeloidea	Cucujiformia	Polyphaga	Wood	Forest	Xylo	Temp	Wide	Neut	Sex	Large	Mobil	0	0	0
204	*Oxythyrea funesta*	Scarabaeidae	Scarabaeoidea	Scarabaeiformia	Polyphaga	Phyto	Open	Herb	Cont	Fragm	Warm	Sex	Med	Mobil	0	0	0
205	*Pachyta quadrimaculata*	Cerambycidae	Chrysomeloidea	Cucujiformia	Polyphaga	Wood	Forest	Epig	Temp	Wide	Neut	Sex	Med	Mobil	1	1	0
206	*Pachytodes cerambyciformis*	Cerambycidae	Chrysomeloidea	Cucujiformia	Polyphaga	Wood	Forest	Xylo	Temp	Wide	Neut	Sex	Med	Mobil	0	0	0
207	*Paederidus rubrothoracicus*	Staphylinidae	Staphylinoidea	Staphyliniformia	Polyphaga	Pred	Open	Hydro	Temp	Wide	Neut	Sex	Med	Mobil	0	0	0
208	*Paederidus ruficollis*	Staphylinidae	Staphylinoidea	Staphyliniformia	Polyphaga	Pred	Open	Hydro	Temp	Wide	Neut	Sex	Med	Mobil	1	1	0
209	*Paederus caligatus*	Staphylinidae	Staphylinoidea	Staphyliniformia	Polyphaga	Pred	Open	Hydro	Temp	Wide	Neut	Sex	Med	Mobil	0	0	0
210	*Paederus limnophilus*	Staphylinidae	Staphylinoidea	Staphyliniformia	Polyphaga	Pred	Open	Hydro	Temp	Wide	Neut	Sex	Med	Mobil	1	1	0
211	*Paederus litoralis*	Staphylinidae	Staphylinoidea	Staphyliniformia	Polyphaga	Pred	Open	Hydro	Temp	Wide	Neut	Sex	Med	Mobil	1	1	0
212	*Paederus riparius*	Staphylinidae	Staphylinoidea	Staphyliniformia	Polyphaga	Pred	Open	Hydro	Temp	Wide	Neut	Sex	Med	Mobil	1	1	1
213	*Paophilus afflatus*	Curculionidae	Curculionoidea	Cucujiformia	Polyphaga	Phyto	Open	Herb	Temp	Fragm	Warm	Sex	Small	Seden	1	1	0
214	*Paradromius linearis*	Carabidae	Caraboidea	Carabiformia	Adephaga	Pred	Open	Epig	Cont	Wide	Warm	Sex	Med	Mobil	0	0	0
215	*Parafoucartia squamulata*	Curculionidae	Curculionoidea	Cucujiformia	Polyphaga	Phyto	Open	Herb	Temp	Wide	Warm	Sex	Small	Seden	1	1	0
216	*Paranchus albipes*	Carabidae	Caraboidea	Carabiformia	Adephaga	Pred	Open	Hydro	Temp	Wide	Neut	Sex	Med	Mobil	0	0	0
217	*Perileptus areolatus*	Carabidae	Caraboidea	Carabiformia	Adephaga	Pred	Open	Hydro	Temp	Wide	Neut	Sex	Small	Mobil	0	0	0
218	*Phosphuga atrata*	Silphidae	Staphylinoidea	Staphyliniformia	Polyphaga	Pred	Ubiq	Epig	Temp	Wide	Neut	Sex	Med	Mobil	0	0	0
219	*Phyllobius brevis*	Curculionidae	Curculionoidea	Cucujiformia	Polyphaga	Phyto	Open	Herb	Temp	Wide	Neut	Sex	Small	Mobil	0	0	0
220	*Phyllobius glaucus*	Curculionidae	Curculionoidea	Cucujiformia	Polyphaga	Phyto	Forest	Herb	Temp	Wide	Neut	Sex	Med	Mobil	1	1	1
221	*Phyllobius maculicornis*	Curculionidae	Curculionoidea	Cucujiformia	Polyphaga	Phyto	Ubiq	Herb	Temp	Wide	Neut	Sex	Small	Mobil	1	1	1
222	*Phyllopertha horticola*	Scarabaeidae	Scarabaeoidea	Scarabaeiformia	Polyphaga	Phyto	Ubiq	Herb	Temp	Wide	Neut	Sex	Med	Mobil	0	0	0
223	*Phyllotreta christinae*	Chrysomelidae	Chrysomeloidea	Cucujiformia	Polyphaga	Phyto	Open	Herb	Medit	Fragm	Neut	Sex	Small	Mobil	0	0	0
224	*Phymatodes testaceus*	Cerambycidae	Chrysomeloidea	Cucujiformia	Polyphaga	Wood	Forest	Xylo	Temp	Wide	Neut	Sex	Med	Mobil	0	0	0
225	*Pidonia lurida*	Cerambycidae	Chrysomeloidea	Cucujiformia	Polyphaga	Wood	Forest	Xylo	Mount	Wide	Neut	Sex	Med	Mobil	0	0	0
226	*Pityogenes chalcographus*	Curculionidae	Curculionoidea	Cucujiformia	Polyphaga	Wood	Forest	Xylo	Temp	Wide	Neut	Sex	Small	Mobil	1	1	0
227	*Plagionotus detritus*	Cerambycidae	Chrysomeloidea	Cucujiformia	Polyphaga	Wood	Forest	Xylo	Temp	Wide	Neut	Sex	Med	Mobil	0	0	0
228	*Platynus assimilis*	Carabidae	Caraboidea	Carabiformia	Adephaga	Pred	Forest	Epig	Temp	Wide	Neut	Sex	Med	Mobil	0	0	0
229	*Poecilus versicolor*	Carabidae	Caraboidea	Carabiformia	Adephaga	Pred	Open	Epig	Temp	Wide	Neut	Sex	Med	Mobil	0	0	0
230	*Polydrusus mollis*	Curculionidae	Curculionoidea	Cucujiformia	Polyphaga	Phyto	Ubiq	Herb	Temp	Wide	Neut	Parth	Med	Mobil	1	1	1
231	*Polydrusus inustus*	Curculionidae	Curculionoidea	Cucujiformia	Polyphaga	Phyto	Open	Herb	Cont	Fragm	Warm	Parth	Small	Seden	1	1	0

232	*Prionus coriarius*	Cerambycidae	Chrysomeloidea	Cucujiformia	Polyphaga	Wood	Forest	Epig	Temp	Wide	Neut	Sex	Med	Mobil	0	0	0
233	*Propylea quatuordecimpunctata*	Coccinellidae	Cucujoidea	Cucujiformia	Polyphaga	Pred	Ubiq	Herb	Temp	Wide	Neut	Sex	Small	Mobil	0	0	0
234	*Pseudomechoris aethiops*	Rhynchitidae	Curculionoidea	Cucujiformia	Polyphaga	Phyto	Open	Herb	Medit	Fragm	Warm	Sex	Small	Mobil	0	0	0
235	*Pseudoperapion ergenense*	Apionidae	Curculionoidea	Cucujiformia	Polyphaga	Phyto	Open	Herb	Temp	Wide	Neut	Sex	Small	Mobil	1	0	1
236	*Pseudovadonia livida*	Cerambycidae	Chrysomeloidea	Cucujiformia	Polyphaga	Myceto	Open	Fungi	Temp	Wide	Neut	Sex	Med	Mobil	0	0	0
237	*Psyllobora vigintiduopunctata*	Coccinellidae	Cucujoidea	Cucujiformia	Polyphaga	Myceto	Ubiq	Fungi	Temp	Wide	Neut	Sex	Small	Mobil	1	1	0
238	*Pterostichus melanarius*	Carabidae	Caraboidea	Carabiformia	Adephaga	Pred	Ubiq	Epig	Temp	Wide	Neut	Sex	Med	Mobil	0	0	0
239	*Pterostichus niger*	Carabidae	Caraboidea	Carabiformia	Adephaga	Pred	Forest	Epig	Temp	Wide	Neut	Sex	Large	Mobil	0	0	0
240	*Pterostichus oblongopunctatus*	Carabidae	Caraboidea	Carabiformia	Adephaga	Pred	Forest	Epig	Temp	Wide	Neut	Sex	Med	Mobil	0	0	0
241	*Pyrochroa coccinea*	Pyrochroidae	Tenebrionoidea	Cucujiformia	Polyphaga	Pred	Forest	Xylo	Temp	Wide	Neut	Sex	Med	Mobil	0	0	0
242	*Pyrrhidium sanguineum*	Cerambycidae	Chrysomeloidea	Cucujiformia	Polyphaga	Wood	Forest	Xylo	Temp	Wide	Neut	Sex	Med	Mobil	0	0	0
243	*Pytho depressus*	Pythidae	Tenebrionoidea	Cucujiformia	Polyphaga	Wood	Forest	Xylo	Temp	Wide	Neut	Sex	Med	Mobil	0	0	0
244	*Rhagium inquisitor*	Cerambycidae	Chrysomeloidea	Cucujiformia	Polyphaga	Wood	Forest	Xylo	Temp	Wide	Neut	Sex	Med	Mobil	0	0	0
245	*Rhagium mordax*	Cerambycidae	Chrysomeloidea	Cucujiformia	Polyphaga	Wood	Forest	Xylo	Temp	Wide	Neut	Sex	Large	Mobil	0	0	0
246	*Rhagium sycophanta*	Cerambycidae	Chrysomeloidea	Cucujiformia	Polyphaga	Wood	Forest	Xylo	Temp	Wide	Warm	Sex	Med	Mobil	0	0	0
247	*Rhagonycha fulva*	Cantharidae	Elateroidea	Elateriformia	Polyphaga	Pred	Ubiq	Herb	Temp	Wide	Neut	Sex	Med	Mobil	0	0	0
248	*Rhagonycha lignosa*	Cantharidae	Elateroidea	Elateriformia	Polyphaga	Pred	Forest	Herb	Temp	Wide	Neut	Sex	Med	Mobil	0	0	0
249	*Rhagonycha limbata*	Cantharidae	Elateroidea	Elateriformia	Polyphaga	Pred	Ubiq	Herb	Temp	Wide	Neut	Sex	Med	Mobil	0	0	0
250	*Rhinusa tetra*	Curculionidae	Curculionoidea	Cucujiformia	Polyphaga	Phyto	Open	Herb	Temp	Wide	Neut	Sex	Small	Mobil	1	0	1
251	*Rhyzobius chrysomeloides*	Coccinellidae	Cucujoidea	Cucujiformia	Polyphaga	Pred	Forest	Herb	Temp	Wide	Neut	Sex	Small	Mobil	0	0	0
252	*Rusticoclytus rusticus*	Cerambycidae	Chrysomeloidea	Cucujiformia	Polyphaga	Wood	Forest	Xylo	Temp	Wide	Neut	Sex	Med	Mobil	0	0	0
253	*Rutpela maculata*	Cerambycidae	Chrysomeloidea	Cucujiformia	Polyphaga	Wood	Forest	Xylo	Temp	Wide	Neut	Sex	Large	Mobil	0	0	0
254	*Salpingus ruficollis*	Salpingidae	Tenebrionoidea	Cucujiformia	Polyphaga	Pred	Forest	Xylo	Temp	Wide	Neut	Sex	Small	Mobil	0	0	0
255	*Saperda scalaris*	Cerambycidae	Chrysomeloidea	Cucujiformia	Polyphaga	Wood	Forest	Xylo	Temp	Wide	Neut	Sex	Med	Mobil	0	0	0
256	*Schizotus pectinicornis*	Pyrochroidae	Tenebrionoidea	Cucujiformia	Polyphaga	Pred	Forest	Xylo	Temp	Wide	Neut	Sex	Med	Mobil	0	0	0
257	*Sciaphobus rubi*	Curculionidae	Curculionoidea	Cucujiformia	Polyphaga	Phyto	Open	Herb	Temp	Wide	Warm	Sex	Small	Seden	1	0	1
258	*Scymnus nigrinus*	Coccinellidae	Cucujoidea	Cucujiformia	Polyphaga	Pred	Forest	Herb	Temp	Wide	Neut	Sex	Small	Mobil	0	0	0
259	*Serropalpus barbatus*	Melandryidae	Tenebrionoidea	Cucujiformia	Polyphaga	Wood	Forest	Xylo	Temp	Wide	Neut	Sex	Large	Mobil	0	0	0
260	*Sibinia pellucens*	Curculionidae	Curculionoidea	Cucujiformia	Polyphaga	Phyto	Open	Herb	Temp	Wide	Neut	Sex	Small	Mobil	1	1	0
261	*Silis nitidula*	Cantharidae	Elateroidea	Elateriformia	Polyphaga	Pred	Ubiq	Herb	Temp	Wide	Neut	Sex	Med	Mobil	0	0	0
262	*Sitona suturalis*	Curculionidae	Curculionoidea	Cucujiformia	Polyphaga	Phyto	Open	Herb	Temp	Wide	Neut	Sex	Small	Mobil	1	0	1
263	*Sitona sulcifrons*	Curculionidae	Curculionoidea	Cucujiformia	Polyphaga	Phyto	Open	Herb	Temp	Wide	Neut	Sex	Small	Mobil	0	0	0
264	*Smaragdina affinis*	Chrysomelidae	Chrysomeloidea	Cucujiformia	Polyphaga	Phyto	Open	Herb	Temp	Wide	Neut	Sex	Small	Mobil	0	0	0
265	*Spermophagus sericeus*	Chrysomelidae	Chrysomeloidea	Cucujiformia	Polyphaga	Phyto	Open	Herb	Temp	Wide	Neut	Sex	Small	Mobil	0	0	0
266	*Sphidus dubius*	Sphindidae	Cucujoidea	Cucujiformia	Polyphaga	Myceto	Forest	Fungi	Temp	Wide	Neut	Sex	Med	Mobil	0	0	0

267	*Spondylis buprestoides*	Cerambycidae	Chrysomeloidea	Cucujiformia	Polyphaga	Wood	Forest	Epig	Temp	Wide	Neut	Sex	Large	Mobil	0	0	0
268	*Staphylinus erythropterus*	Staphylinidae	Staphylinoidea	Staphyliniformia	Polyphaga	Pred	Forest	Epig	Temp	Wide	Neut	Sex	Med	Mobil	1	1	0
269	*Stenolophus teutonus*	Carabidae	Caraboidea	Carabiformia	Adephaga	Pred	Open	Epig	Temp	Wide	Neut	Sex	Med	Mobil	0	0	0
270	*Stenomax aeneus*	Tenebrionidae	Tenebrionoidea	Cucujiformia	Polyphaga	Sapro	Forest	Xylo	Temp	Fragm	Neut	Sex	Med	Seden	1	1	0
271	*Stenurella melanura*	Cerambycidae	Chrysomeloidea	Cucujiformia	Polyphaga	Wood	Forest	Xylo	Temp	Wide	Neut	Sex	Med	Mobil	0	0	0
272	*Stenurella nigra*	Cerambycidae	Chrysomeloidea	Cucujiformia	Polyphaga	Wood	Forest	Xylo	Temp	Wide	Neut	Sex	Med	Mobil	0	0	0
273	*Strangalia attenuata*	Cerambycidae	Chrysomeloidea	Cucujiformia	Polyphaga	Wood	Forest	Xylo	Temp	Wide	Neut	Sex	Med	Mobil	0	0	0
274	*Strophosoma capitatum*	Curculionidae	Curculionoidea	Cucujiformia	Polyphaga	Phyto	Ubiq	Herb	Cont	Wide	Neut	Sex	Small	Seden	1	1	1
275	*Strophosoma faber*	Curculionidae	Curculionoidea	Cucujiformia	Polyphaga	Phyto	Open	Herb	Cont	Wide	Neut	Sex	Small	Seden	0	0	0
276	*Strophosoma melanogrammum*	Curculionidae	Curculionoidea	Cucujiformia	Polyphaga	Phyto	Ubiq	Herb	Cont	Wide	Neut	Parth	Small	Seden	1	0	1
277	*Synapion ebeninum*	Apionidae	Curculionoidea	Cucujiformia	Polyphaga	Phyto	Open	Herb	Temp	Wide	Neut	Sex	Small	Mobil	0	0	0
278	*Synuchus vivalis*	Carabidae	Caraboidea	Carabiformia	Adephaga	Pred	Ubiq	Epig	Temp	Wide	Neut	Sex	Med	Mobil	0	0	0
279	*Tachyta nana*	Carabidae	Caraboidea	Carabiformia	Adephaga	Pred	Forest	Xylo	Temp	Wide	Neut	Sex	Small	Mobil	0	0	0
280	*Tanymecus palliatus*	Curculionidae	Curculionoidea	Cucujiformia	Polyphaga	Phyto	Open	Herb	Temp	Wide	Neut	Sex	Small	Mobil	1	1	0
281	*Tenebrio molitor*	Tenebrionidae	Tenebrionoidea	Cucujiformia	Polyphaga	Sapro	Ubiq	Xylo	Temp	Wide	Neut	Sex	Med	Mobil	0	0	0
282	*Tetropium castaneum*	Cerambycidae	Chrysomeloidea	Cucujiformia	Polyphaga	Wood	Forest	Xylo	Temp	Wide	Neut	Sex	Small	Mobil	0	0	0
283	*Tetropium fuscum*	Cerambycidae	Chrysomeloidea	Cucujiformia	Polyphaga	Wood	Forest	Xylo	Temp	Wide	Neut	Sex	Med	Mobil	1	1	0
284	*Thanasimus formicarius*	Cleridae	Cleroidea	Cucujiformia	Polyphaga	Pred	Forest	Xylo	Temp	Wide	Neut	Sex	Med	Mobil	0	0	0
285	*Thanatophilus sinuatus*	Silphidae	Staphylinoidea	Staphyliniformia	Polyphaga	Sapro	Ubiq	Necro	Temp	Wide	Neut	Sex	Med	Mobil	0	0	0
286	*Trachys minuta*	Buprestidae	Buprestoidea	Elateriformia	Polyphaga	Phyto	Forest	Herb	Temp	Wide	Neut	Sex	Small	Mobil	1	1	0
287	*Trichodes apiarius*	Cleridae	Cleroidea	Cucujiformia	Polyphaga	Pred	Open	Epig	Medit	Wide	Warm	Sex	Med	Mobil	0	0	0
288	*Triplax aenea*	Erotylidae	Cucujoidea	Cucujiformia	Polyphaga	Myceto	Forest	Fungi	Temp	Wide	Neut	Sex	Small	Mobil	1	1	0
289	*Triplax russica*	Erotylidae	Cucujoidea	Cucujiformia	Polyphaga	Myceto	Forest	Fungi	Temp	Wide	Neut	Sex	Med	Mobil	0	0	0
290	*Tritoma bipustulata*	Erotylidae	Cucujoidea	Cucujiformia	Polyphaga	Myceto	Forest	Fungi	Temp	Wide	Neut	Sex	Small	Mobil	0	0	0
291	*Tropinota hirta*	Scarabaeidae	Scarabaeoidea	Scarabaeiformia	Polyphaga	Phyto	Open	Herb	Temp	Wide	Warm	Sex	Med	Mobil	0	0	0
292	*Trypocopris vernalis*	Geotrupidae	Scarabaeoidea	Scarabaeiformia	Polyphaga	Sapro	Ubiq	Copro	Temp	Wide	Neut	Sex	Large	Mobil	0	0	0
293	*Trypodendron lineatum*	Curculionidae	Curculionoidea	Cucujiformia	Polyphaga	Myceto	Forest	Xylo	Temp	Wide	Neut	Sex	Small	Mobil	0	0	0
294	*Tytthaspis sedecimpunctata*	Coccinellidae	Cucujoidea	Cucujiformia	Polyphaga	Myceto	Open	Herb	Medit	Wide	Neut	Sex	Small	Mobil	0	0	0
295	*Uleiota planata*	Silvanidae	Staphylinoidea	Staphyliniformia	Polyphaga	Sapro	Forest	Xylo	Temp	Wide	Neut	Sex	Med	Mobil	0	0	0
296	*Uloma culinaris*	Tenebrionidae	Tenebrionoidea	Cucujiformia	Polyphaga	Sapro	Forest	Xylo	Temp	Wide	Neut	Sex	Med	Seden	1	1	0
297	*Valgus hemipterus*	Scarabaeidae	Scarabaeoidea	Scarabaeiformia	Polyphaga	Wood	Ubiq	Herb	Temp	Wide	Neut	Sex	Med	Mobil	0	0	0

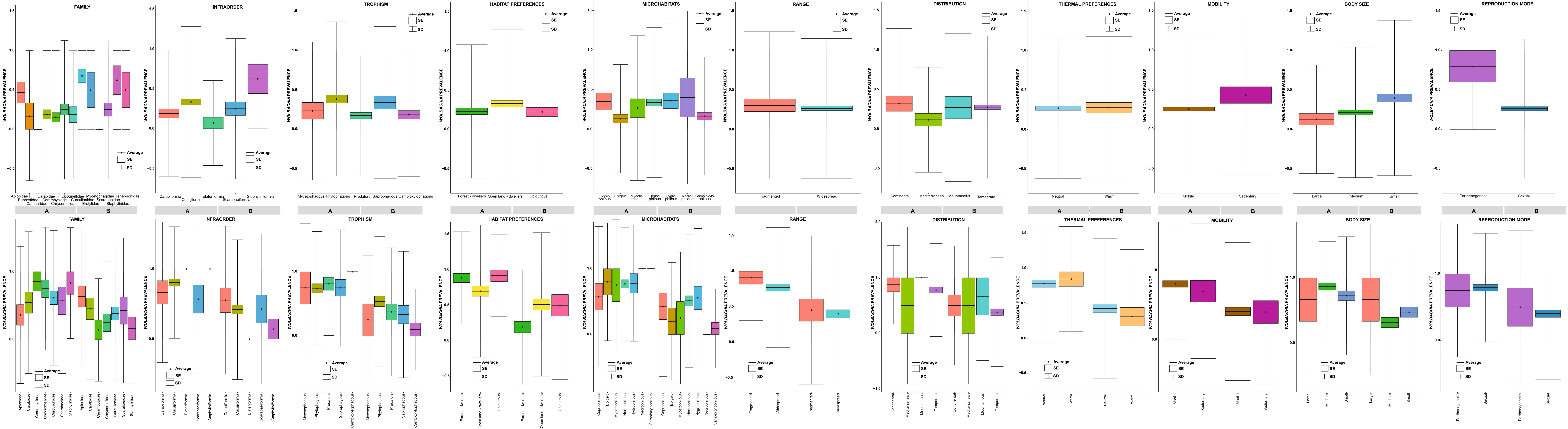

Supplementary Figure S1. Box plots illustrating differences in *Wolbachia* infection (upper panels) and prevalence of supegroups (lower panels) across taxonomic units of beetles (A) and ecological/biological traits of beetles (B).

Supplementary Figure S2. *Wolbachia* phylogenetic tree (reconstructed on the basis of *ftsZ* sequences obtained from infected beetle hosts from this study and all available data from GenBank and MLST databases).

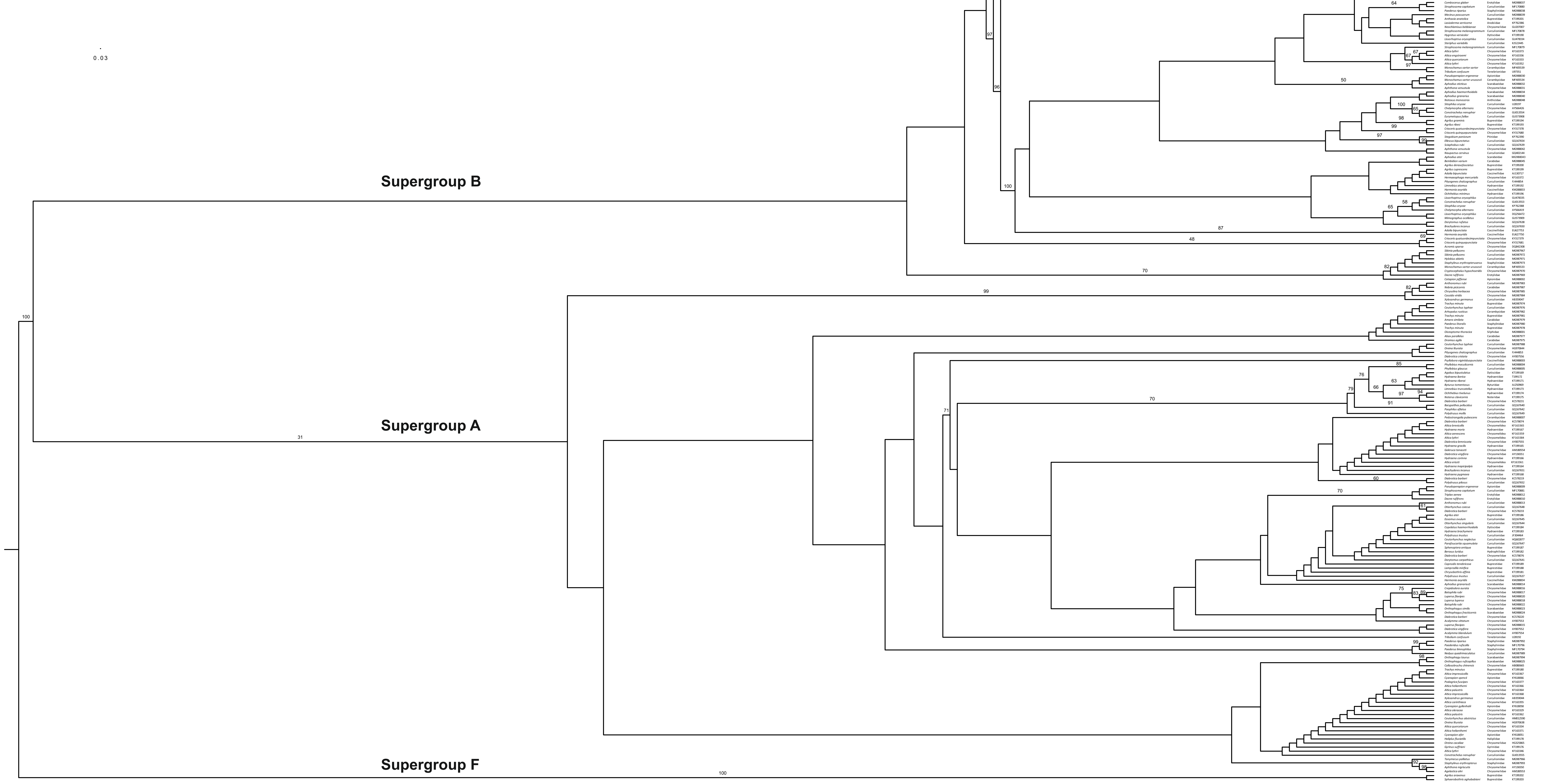

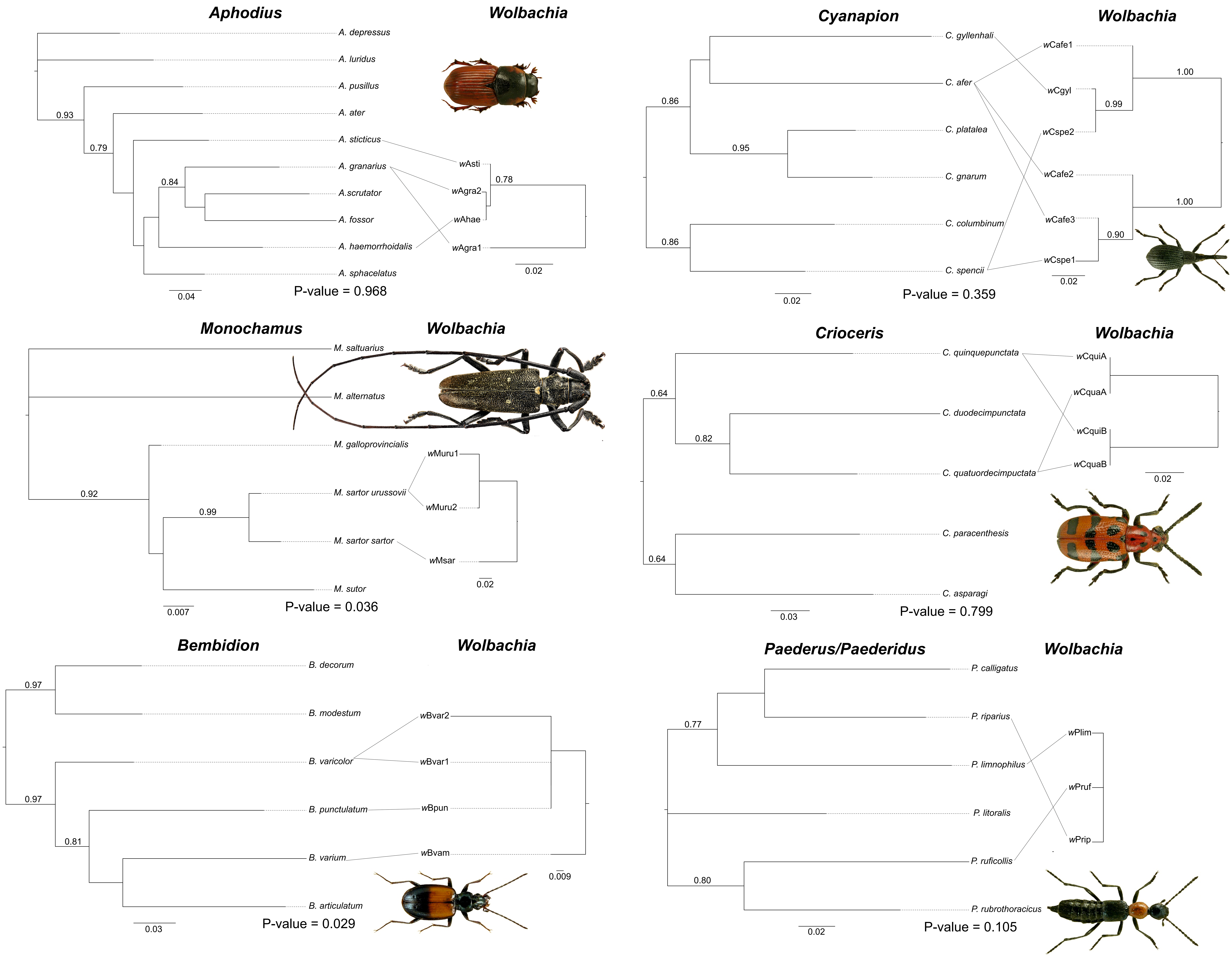

Supplementary Figure S3. Phylogenetic trees of selected groups of beetles (reconstructed on the basis of coxI sequences) and *Wolbachia* strains (reconstructed on the basis of selected housekeeping genes from multilocus sequence typing system) with results of cospeciation tests (performed in Procrustean Approach to Cophylogeny). Photographs are reprinted from ICONOGRAPHIA COLEOPTERORUM POLONIAE under a CC BY license, with permission (© Copyright by Prof. Lech Borowiec, Wrocław 2007–2018, Department of Biodiversity and Evolutionary Taxonomy, University of Wroclaw, Poland)).

Supplementary File 1. Google file (kmz) with localization of sampling sites listed in Supplementary Table 4.

Data to map ©2018 GeoBasis-DE/BKG (© 2009) Google, Inst. Geogr.National.

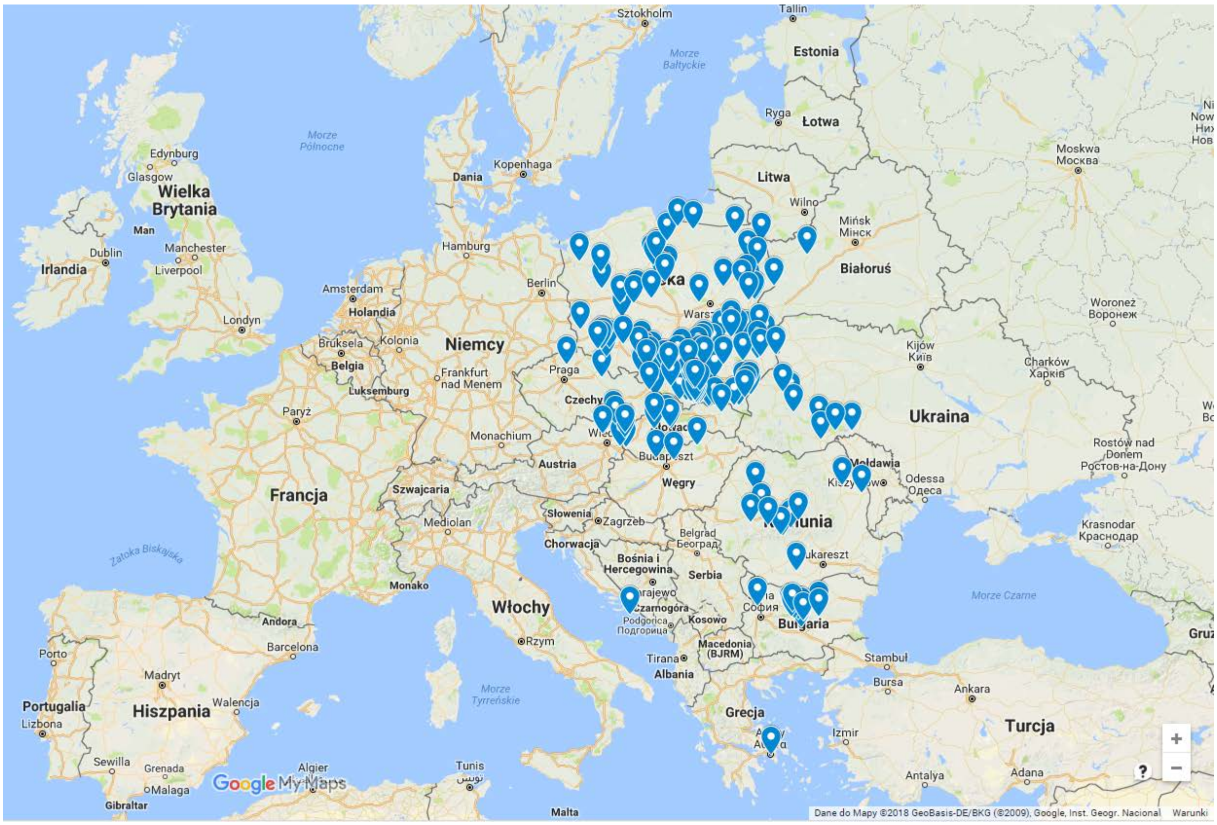

Original kmz file available under following link:

https://drive.google.com/open?id=15yhgviGlAikY0EAkCeDbY_PdDlbKMdQs&usp=sharing

Supplementary File 2. List of references to keys and handbooks used for beetle species identification and for collection of their ecological and biological traits used for statistical analyses.

Burakowski B., Mroczkowski M., Stefańska J. *Chrząszcze Coleoptera, Biegaczowate - Carabidae, część 1. Kat. Fauny Pol., Warszawa*, XXIII, **2**: 1-232 (1973).

Burakowski B., Mroczkowski M., Stefańska J. *Chrząszcze Coleoptera – Biegczowate – Carabidae, część 2. Katalog Fauny Polski,* Warszawa, XXIII, **3**: 1-430 (1974).

Burakowski B., Mroczkowski M., Stefańska J. *Chrząszcze Coleoptera – Adephaga prócz Carabidae, Myxophaga, Polyphaga: Hydrophiloidea. Katalog Fauny Polski,* Warszawa, XXIII, **4**: 1-307 (1976).

Burakowski B., Mroczkowski M., Stefańska J. *Chrząszcze Coleoptera, Histeroidea i Staphylinoidea prócz Staphylinidae. Katalog Fauny Polski*, PWN, Warszawa, XXIII, **5**: 1-356 (1978).

Burakowski B., Mroczkowski M., Stefańska J. *Chrząszcze Coleoptera, Kusakowate – Staphylinidae, cz. 1. Katalog Fauny Polski*, PWN, Warszawa, XXIII, **6**: 1-310 (1979).

Burakowski B., Mroczkowski M., Stefańska J. *Chrząszcze Coleoptera, Kusakowate – Staphylinidae, cz. 2. Katalog Fauny Polski, PWN*, Warszawa, XXIII, **7**: 1-272 (1980).

Burakowski B., Mroczkowski M., Stefańska J. *Chrząszcze Coleoptera, Kusakowate – Staphylinidae, cz. 3: Aleocharinae. Katalog Fauny Polski*, PWN, Warszawa, XXIII, **8**: 1-330 (1981).

Burakowski B., Mroczkowski M., Stefańska J. *Chrząszcze Coleoptera. Scarabaeoidea, Dascilloidea, Byrrhoidea i Parnoidea. Katalog Fauny Polski*, Warszawa, XXIII, **9**: 1-294 (1983).

Burakowski B., Mroczkowski M., Stefańska J. *Chrząszcze Coleoptera. Buprestoidea, Elateroidea i Cantharoidea. Katalog Fauny Polski,* Warszawa, XXIII, **10**: 1-401 (1985).

Burakowski B., Mroczkowski M., Stefańska J. *Chrząszcze Coleoptera – Dermestoidea, Bostrichoidea, Cleroidea i Lymexyloidea. Katalog Fauny Polski,* Warszawa, XXIII, **11**: 1-243 (1986a).

Burakowski B., Mroczkowski M., Stefańska J. *Chrząszcze Coleoptera – Cucujoidea, część 1. Katalog Fauny Polski,* Warszawa, XXIII, **12**: 1-266 (1986b).

Burakowski B., Mroczkowski M., Stefańska J. *Chrząszcze Coleoptera – Cucujoidea, część 3. Katalog Fauny Polski*, Warszawa, XXIII, **14**: 1-309 (1987).

Burakowski B., Mroczkowski M., Stefańska J. 1990. Chrząszcze Coleoptera. Cerambycidae i Bruchidae. Katalog fauny Polski, Warszawa, XXIII, **15**: 1-312 (1990).

Burakowski B., Mroczkowski M., Stefańska J. *Chrząszcze Coleoptera. Stonkowate – Chrysomelidae, część 2. Katalog Fauny Polski,* Warszawa, XXIII, **17**: 1-227 (1991).

Burakowski B., Mroczkowski M., Stefańska J. *Chrząszcze – Coleoptera. Ryjkowcowate prócz ryjkowców – Curculionioidea prócz Curculionidae. Katalog Fauny Polski,* Warszawa, XXIII, **18**: 1-324 (1992).

Burakowski B., Mroczkowski M., Stefańska J. *Chrząszcze – Coleoptera, Ryjkowce – Curculionidae część 2. Katalog Fauny Polski*. MiIZ PAN, Warszawa, XXIII, **20**: 1-310 (1995).

Burakowski B., Mroczkowski M., Stefańska J. *Chrząszcze – Coleoptera, Ryjkowce – Curculionidae część 3. Katalog Fauny Polski*. MiIZ PAN, Warszawa, XXIII, **21**: 1-307 (1997).

Burakowski B., Mroczkowski M., Stefańska J. *Chrząszcze Coleoptera. Uzupełnienia tomów 2-21. Katalog Fauny Polski*. MiIZ PAN, Warszawa, XXIII, **22**: 1-252 (2000).

Koch, K., 1989a: Die Käfer Mitteleuropas. Ökologie. Band 1. Goecke & Evers, Krefeld, 440 pp (1989a).

Koch, K., 1989b: Die Käfer Mitteleuropas. Ökologie. Band 2. Goecke & Evers, Krefeld, 382 pp (1989b).

Koch, K., 1992: Die Käfer Mitteleuropas. Ökologie. Band 3. Goecke & Evers, Krefeld, 389 pp (1992).

Löbl I., Smetana A. (eds.). *Catalogue of Palaearctic Coleoptera. Vol. 1. Archostemata – Myxophaga – Adephaga.* Brill, Leiden Boston, 819 pp (2003).

Löbl I., Smetana A. (eds.). *Catalogue of Palaearctic Coleoptera. Vol. 4. Elateroidea – Derodontoidea – Bostrichoidea – Lymexyloidea – Cleroidea – Cucujoidea*. Apollo Books, Stenstrup, 935 pp (2007).

Löbl I., Smetana A. (eds.). *Catalogue of Palaearctic Coleoptera. Vol. 5. Tenebrionoidea.* Apollo Books, Stenstrup, 670 pp (2008).

Löbl I., Smetana A. (eds.). *Catalogue of Palaearctic Coleoptera. Vol. 6. Chrysomeloidea*. Apollo Books, Stenstrup, 924 pp (2010).

Löbl I., Smetana A. (eds.). *Catalogue of Palaearctic Coleoptera. Vol. 7. Curculionoidea I.* Apollo Books, Stenstrup, 373 pp (2011).

Löbl I., Smetana A. (eds.) *Catalogue of Palaearctic Coleoptera. Vol. 8. Curculionoidea II.* Leiden, Brill, 700 pp (2013).
